# The new Lyon ARTbrace versus the historical Lyon brace: a prospective case series of 148 consecutive scoliosis with short time results after 1 year compared with a historical retrospective case series of 100 consecutive scoliosis; SOSORT award 2015 winner

**DOI:** 10.1186/s13013-015-0047-6

**Published:** 2015-08-19

**Authors:** Jean Claude de Mauroy, Alexandre Journe, Fabio Gagaliano, Cyril Lecante, Frederic Barral, Sophie Pourret

**Affiliations:** Clinique du Parc, 155, boulevard Stalingrad, Lyon, 69006 France; Orten 125 Rue Bataille, Lyon, 69008 France

## Background

Adolescent idiopathic scoliosis (AIS) is a four dimensional deformity of the spine arising in otherwise healthy children during puberty. The fourth dimension is time. This dimension is the characteristic of our database created in 1998 with systematic reconvening of our patients at regular intervals which increases the level of scientific evidence [1]. The use of a brace in the conservative treatment for AIS plays an important role and has the aim to stop the evolution of the deformity in immature adolescents in order to prevent problems during adulthood [2, 3]. Long-term follow-ups indicate that patients with scoliosis may have a higher prevalence of back pain and of worsening pulmonary function if the curve becomes extremely severe [4]. A randomized control trial BRAIST study conducted by Weinstein showed that bracing is significantly effective in reducing the progression of AIS [5]. Previously, a Cochrane review [6] also demonstrated the effectiveness of bracing in the treatment of AIS.

To measure the effectiveness of a brace two main factors can be involved: 1. the immediate in-brace reduction depending how to get the three-dimensional correction and its reproducibility; 2. the patient’s adherence which depends on aesthetics and tolerance [7, 8]. Different types of braces are used in the treatment of AIS but almost all are created on the multiple three points system principle of applying external corrective forces across the curve in order to stop deformity progression, produce an acceptable sagittal and coronal contour, and delay or avoid surgical treatment [9–12]. The main biomechanical concepts are based on: elongation along the vertical axis, lateral inflexion in the frontal plane and derotation of the spine in order to obtain a correction of the scoliotic curve. Derotation is the main movement along the vertical axis. The correction in the sagittal plane is problematic because many scoliosis are accompanied by a change in the sagittal plane with a flat back in half of the cases. All of the above mechanisms are going in the direction of accentuation of the flat back and require significant and uncertain changes during the manufacture of the brace. This problem has now been finally solved thanks to segmental moulding.

### Elongation

Historically, in the early twentieth century, in the United States, Sayre [13] was the first to make a plaster cast in a standing posture using this biomechanical concept, even if the first modern brace can be considered the Milwaukee brace, created in 1940 by Blount, which was a brace based on axial elongation between the pelvis and the cervical collar.

**Fig. 1:**
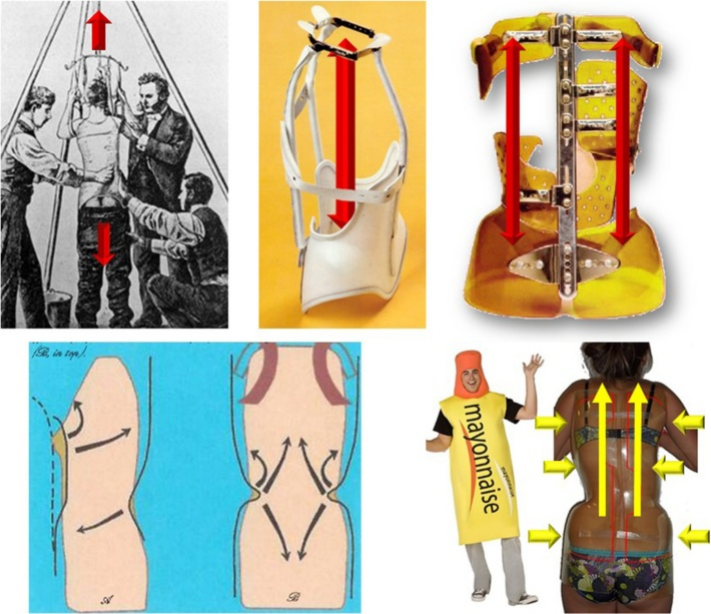
Evolution of elongation concepts along the vertical axis. At the time of the first Sayre’s plaster cast, cervical suspension and body weight realize a bipolar overall elongation. With the Milwaukee brace, the elongation is internal between pelvic girdle and cervical collar. With the Lyon brace, elongation occurs between the pelvic and shoulder girdle with equal distribution of forces on the right and on the left. The elongation requires precise adjustment of the brace during the growth of the child. Other TLSO braces introduce a new concept described by Chêneau as “cherry stone effect” with stretching upwards between pelvis and rib cage. The existence of windows in the brace do not affect elongation. In contrast, with the new Lyon brace, axial elongation type “mayonnaise tube” is achieved by the simultaneous clamp of the two hemi polycarbonate pieces and requires the integrity of the external tube wall

In France, the Lyon brace, created in 1947 by Pierre Stagnara, was the first 3D adjustable contention brace used after a plaster cast. With the Lyon brace, elongation occurs between the pelvic and shoulder girdle with equal distribution of forces on the right and on the left. The elongation requires precise adjustment of the brace during the growth of the child [14]. Other TLSO braces introduce a new concept described by Chêneau as the “cherry stone effect” with stretching upwards between pelvis and rib cage. The existence of windows in the brace do not affect elongation. In contrast, with the new Lyon brace, axial elongation type “mayonnaise tube” is achieved by the simultaneous clamp of the two hemi polycarbonate pieces and requires the integrity of the outer tube wall (Fig. 1).

### Derotation and Detorsion or Untwisting

**Fig. 2:**
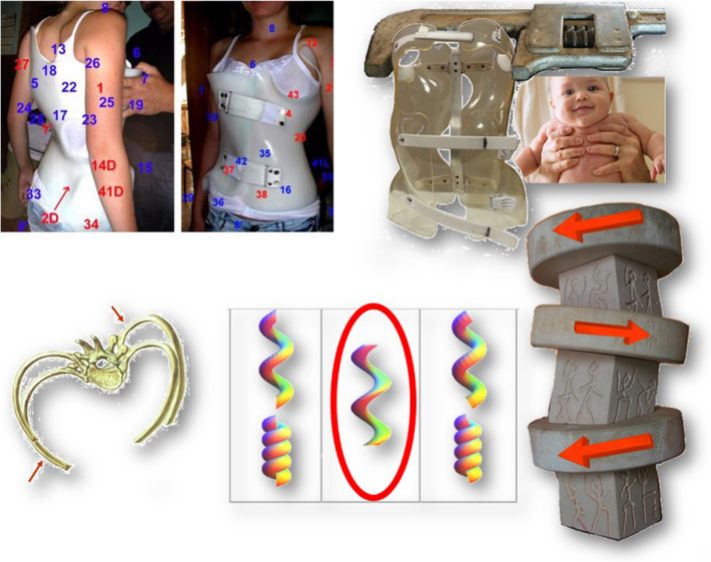
From segmental derotation to global detorsion or untwisting. The segmental derotation is difficult to achieve because it is done via the ribs and could increase flat back. It is impossible to obtain when the rib hump is angular. The mathematical basis of the twisted column is the circled helicoid with horizontal generating circle. The overall untwisting occurs between the axillary and pelvic clamps and the thoracolumbar horizontal plane

The segmental derotation is difficult to achieve because it is done through the ribs and could lead to an increase in a flat back. It is impossible to achieve derotation when the rib hump is angular. The mathematical basis of the twisted column is the circled helicoid with horizontal generating circle. The overall untwisting occurs between the axillary and pelvic clamps and the thoracolumbar horizontal plane (Fig. 2).

Cotrel added a fundamental component: the flexion in the frontal plane [14, 15]. The acronym ‘EDF’ stands for: Elongation, Derotation, Flexion. He created a framework for three-dimensional scoliosis correction in the supine position with spine untwisting. At the end of plaster cast weaning, the plaster mould to build the Lyon brace reproduces the overcorrection obtained [14, 16].

**Fig. 3:**
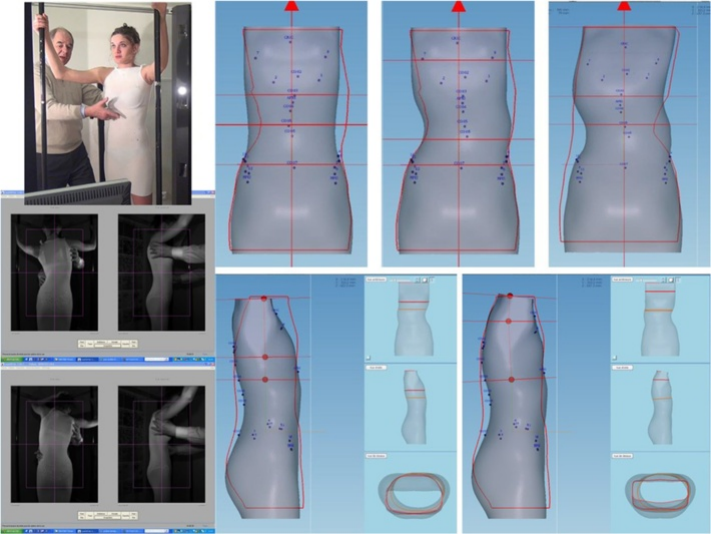
Segmental moulding. Segmental molding is one of the fundamental innovations of ART brace. The overcorrection is performed in the frontal plane and the sagittal plane precisely and individually for each child. The detorsion is obtained by untwisting coupled movements. Chêneau brace is also a night & day overcorrecting brace, but the overcorrection is only made by the CPO. ARTbrace is a custom overcorrecting brace. It is the patient himself who will determine the overcorrection

Many previous studies support the positive results associated with the casting and Lyon braces [14, 16, 17] but the difficulty and cost of making the plaster cast, administrative economical questions and low compliance, can also explain the reasons which ultimately have prompted the development of new design concepts with immediate in-brace correction. It was only in 2013 that advances in computer technology with the latest generation software (OrtenShape) allowed the superposition of different CAD/CAM moulds and a segmental 3D reconstruction [18, 19]. The aim was to use this new software to replace the plaster cast with a new Lyon brace: the ARTbrace. Segmental moulding is one of the fundamental innovations of the ART brace. The overcorrection is performed in the frontal plane and the sagittal plane precisely and individually for each child at three levels: pelvis, lumbar spine and thoracic spine. The detorsion is obtained by untwisting coupled movements. The Chêneau brace is also a night and day overcorrecting brace, but the overcorrection is only made by the CPO. The ARTbrace is a custom night and day overcorrecting brace. It is the patient himself who will determine the overcorrection (Fig. 3).

#### High Rigidity

Even if the old Lyon brace in polymethacrylate was very rigid, the credit for HIGH RIGIDITY goes to the Italian team of ISICO with the Sforzesco brace, which has proven to be effective by avoiding plaster casts for scoliosis over 45° [20]. The acronym ARTbrace (Asymmetrical Rigid Torsion brace) was created by Stefano Negrini. The merit of the ARTbrace is the addition of overcorrection to the high rigidity with a global detorsion. It is this overcorrection for small curvatures which explains the average improvement of the in-brace correction.

Since May 2013, all patients of JCdM were treated with the ARTbrace instead of a plaster cast which showed good initial results. Indeed, the first immediate results of the ARTbrace have demonstrated that the in-brace correction of the Cobb angle in the first 225 cohort of patients was 70 %, a correction which is 40 % higher than with the former Lyon brace or historical Lyon brace. The value of this correction was even higher than for other braces published in the literature, including retrospective studies [21, 22, 8, 23, 24].

Like the historical Lyon brace, the ARTbrace is ADJUSTABLE. Both axillary and pelvic clamps are adjustable with a precise wrench and a bolt system and an anterior ratcheting buckle.

Like the historical Lyon brace, the SAGITTAL PLANE is fixed by the posterior bar. But the sagittal plane is determined by the segmental mould and the superposition of the mouldings. In additional it is the lack of support at the sterno-clavicular level and at the abdominal level that avoids lumbar delordosis and thoracic flat back.

In this study, early results of 148 first consecutive scoliosis treated with the ART-brace after 1-year are reported in correlation with a matched pair control of the last 100 patients treated with the old Lyon Brace.

## Material and Methods

### Study design

We performed a prospective case series of 148 scoliosis with short time results after 1 year compared with a historical retrospective case series of 100 scoliosis. Consecutive cases are recruited in both groups. Randomization was not possible due to the administrative impossibility to perform plaster casts after May 2013. All lumbar scoliosis Lenke 5 were eliminated in the two groups as they continue to be treated by the GTB short brace [25].

### Population

Since May 2013, we treated more than 400 patients at the “Clinique du Parc – Lyon” with the new Lyon brace (ARTbrace) instead of the classical EDF plaster cast followed by the historical Lyon brace. The initial aim was to avoid a plaster cast, but very quickly, the ARTbrace appeared to be a much more effective solution compared to the former plaster casts and it was even better tolerated. Following the early successes the whole treatment was continued with the same brace. In this prospective study, only the first 148 of all patients, 17 % of males and 83 % of females with an average age of 13.37, with a follow-up at 1 year, have been included. The patients of this main group presented 35 thoracic primary curves and 28 lumbar or thoraco-lumbar primary curves and 37 double major curves with a Cobb angle ranging from 20° to 53° (average 29.23° and Standard Deviation: 8.14°). These 148 patients are group A. The second matched pair control group consisted of a consecutive series of 100 patients (22 % males 78 % Females) and an average age of 13.6, treated with a plaster cast and the historical Lyon brace, and controlled 1-year after brace fitting, with 41 thoracic primary curves, 23 primary lumbar or thoraco-lumbar curves and 36 double major curves with a Cobb angle ranging from 20° to 52° (average angle Cobb 30.4° and Standard Deviation: 9.61°). These 100 patients are group B.

All treatment parameters like indications, physiotherapy, full or part-time bracing were identical for both groups, according to the experience of Lyon management [14, 16, 17]. The plaster cast time was replaced by an equivalent time of “full time” ARTbrace.

The study of dropouts is fundamental and we expected a high rate because the realization of the plaster cast was a barrier that only 2/3 of children were crossing. Lyon bracing management has always been considered as an elitist treatment. After 1 year the number of dropouts is 14 (162-148) about 10 %. Some patients referred by colleagues from other countries and controlled by them are not considered as dropouts.

#### Method

All patients were evaluated radiologically before treatment (T0), in-brace (T1), at 6 months without brace (T2) and at 1 year without brace (T3) during treatment. Clinical evaluation at T1 is performed at the end of full time wearing. The clinical parameters were identical for both groups and consisted of measure rib hump in millimetres, and Bunnel ATR by Adam’s posture.

**Fig. 4:**
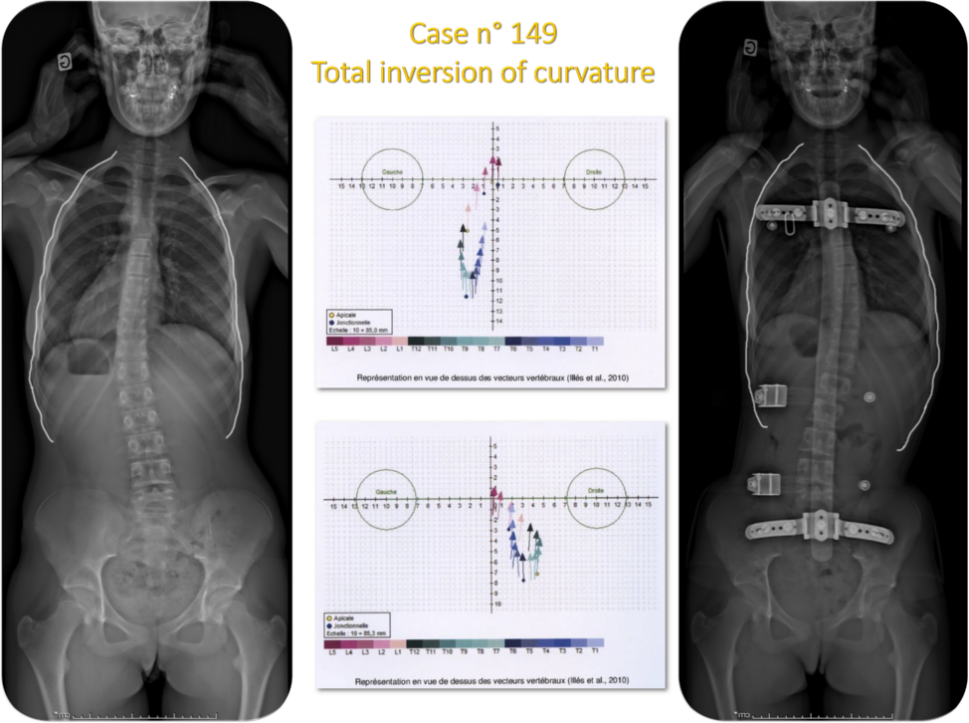
Overcorrection of a long lateral thoraco-lumbar curve. When the deviation is accompanied by very little deformation of the vertebral body, it is possible to completely reverse the curvature with an overcorrecting brace. Stereos shows the complete translation mirrored. There is also a realignment of the curves in the sagittal plane of the spine with recentering on the gravity line. Such result can be expected with many other asymmetrical braces but there is only one asymmetrical high rigidity brace

The radiological examination of ARTbrace group A was performed with an EOS micro dose radiological System, an ultra-low dose radiation imaging system that provides simultaneous AP and Lateral views in the standing position with 25 times less radiation than traditional X-ray, equivalent to one week of natural Earth radiation [26–28]. The standing frontal Cobb angle was always measured by the first author. Automatic measurement EOS cannot be used due to some inversion of curves (Fig. 4).

An automated management system control facilitates regularity in follow up meetings. A 3D sterEOS study was carried out every time we had the EOS radiography at T0.

The radiological follow-up of control group B patients was performed without a sagittal view due to radiation saving habits with traditional radiology and probably also because the correction in the sagittal plane was not perfect. The problem of radiation in scoliosis was discussed in the consensus session of the SOSORT 2011 meeting [29]. In fact to avoid excessive radiation, exposition lateral view X-ray was not systematically executed for most patients. On the contrary, thanks to using ultra-low dose EOS system, a systematically sagittal analysis of spine was possible for the patients in the main group A. The sagittal parameters like Sacral Slope (SS), Lumbar Lordosis (LL) and Thoracic Kyphosis (CT) were automatically measured by EOS system.

All the data are recorded immediately into a database and a serial number is automatically assigned at T1 about 3 days after bracing. All other statistical tests are done with the package SPSS v20. The first step is to confirm the normality of distribution (Kolmogorov-Smirnov & Shapiro-Wilk) and then use an independent-samples T test to compare Cobb angles T0 (before brace), T1 (in-brace),T2 (at 6 months) and T3 (at 1 year). A p value of less than 0.05 was considered to be significant. A copy of the Excel database can be downloaded to allow any comparisons (Additional file 1).

## Results

### Clinical findings

We present the very short results at 6 months in both groups, demonstrating the superiority of the new Lyon brace. The main results on Rib hump (RH) and Bunnel’s ATR (Bu) are shown in Table 1.

**Table 1:** Average and Standard Deviations of Rib hump and Bunnel ATR before bracing and at 6 months for group A (ARTbrace) and Group B (old Lyon brace)

	RH T0	Bu T0	RH T2	Bu T2
T – ARTbrace (A)	23.44 ± 9.4	9.75 ± 4.1	10.33 ± 6.6	5.14 ± 3.3
T - Old Lyon (B)	23.56 ± 8.6	10.55 ± 3.9	16.7 ± 8.5	7.95 ± 4.0
L – ARTbrace (A)	17.21 ± 7.8	7.51 ± 3.6	4.65 ± 4.5	2.06 ± 2.4
L – Old Lyon (B)	16.41 ± 7.4	7.47 ± 3.4	9.41 ± 6.3	4.51 ± 3.2

There was not a significant difference in the score of Thoracic rib hump before brace for control group with the old Lyon brace (M = 23.56, SD = 8.61) and the ARTbrace group (M = 23.44, SD = 9.43, t(176) = 0.089, *p* = 0.929.

There was not a significant difference in the score of Thoracic Bunnel ATR before brace for control group with the old Lyon brace (M = 10.55, SD = 3.85) and the ARTbrace group (M = 9.75, SD = 4.10, t(176) = 1.307, *p* = 0.193.

There was not also a significant difference in the score of Lumbar rib hump before brace for control group with the old Lyon brace (M = 16.41, SD = 7.36) and the ARTbrace group (M = 17.21, SD = 7.76, t(154) = -0.612, *p* = 0.541.

There was not also a significant difference in the score of Lumbar Bunnel ATR before brace for control group with the old Lyon brace (M = 7.47, SD = 3.378) and the ARTbrace group (M = 7.51, SD = 3.63, t(154) = -0.072, *p* = 0.943.

There was a significant difference in the scores for thoracic rib hump and Bunnel ATR and for lumbar rib hump and Bunnel ATR, at 6 months between the two groups.

Thoracic rib hump: t(176) = 5.651, *p* = 0.00

Thoracic Bunnel ATR: t(176) = 5.104, *p* = 0.00

Lumbar rib hump: t(155) = 5.459, *p* = 0.00

Lumbar Bunnel ATR: t(155) = 5.304, *p* = 0.00

Group A (ARTbrace)

At the thoracic level the percentage improvement is: 57 % for rib hump and 51 % for ATR

At the lumbar level the percentage improvement is: 79 % for rib hump and 86 % for ATR

Group B (Historical Lyon brace)

At the thoracic level the percentage improvement is: 27 % for rib hump and 25 % for ATR

At the lumbar level the percentage improvement is: 53 % for rib hump and 49 % for ATR

The percentage improvement between the old and the new Lyon brace is near 30 % for both rib hump and ATR. It is better for the lumbar area compared with the thoracic one.

### Radiological findings

#### Frontal correction

The main group A of 148 patients (ARTbrace) had 195 primary curves from 20° to 55°: 63 curve Thoracic, 42 curve lumbar with 45 double major curves. Only primary curves were selected.

The control group B of 100 patients (Historical Lyon brace) had 136 curves from 20° to 50°: 41 Thoracic curves 23 lumbar curves with 36 double major curves. Only primary curves were selected (Table 2).

**Table 2:** Average and Standard Deviation of Cobb angle at T0 (before bracing), T1 (in-brace), T2 (6 months), T3 (1 year), for group A (ARTbrace) and Group B (old Lyon brace)

*Cobb angle*	T0 Initial	T1 In-brace	T2 6 months	T3 1 year
A-Tho ART (*n* = 108)	30.03 ± 9.6	11.26 ± 8.65	20.25 ± 11	21.47 ± 11
A-Lumb ART (*n* = 87)	27.83 ± 7.5	6.64 ± 8.8	15.50 ± 9.1	16.40 ± 9.38
B-Tho Hist (*n* = 76)	31.14 ± 9.6	16.96 ± 9.3	23.96 ± 11	26.95 ± 11.9
B-Lumb Hist (*n* = 59)	29.69 ± 7.7	12.32 ± 7.9	18.81 ± 9.4	20.41 ± 11

The percentage of improvement was calculated using the following formula: (average T0 – average T1)/average T0 and so on for T2 and T3 (Table 3).

**Table 3:** Percentage improvement relative to the initial angle at T1 (in-brace), T2 (6 months), T3 (1 year), for group A (ARTbrace) and Group B (old Lyon brace)

*Percentage improvement*	(T0-T1)/T0	(T0-T2)/T0	(T0-T3)/T0
A-Tho ART (*n* = 108)	62.5 %	32.6 %	28.5 %
A-Lumb ART (*n* = 87)	76.1 %	44.3 %	41.1 %
B-Tho Hist (*n* = 76)	45.5 %	23.0 %	13.5 %
B-Lumb Hist (*n* = 59)	58.5 %	26.5 %	31.2 %

To compare the progression between the two groups the differential was calculated using the following formula: (percentage A – percentage B)/percentage B for T1, T2 and T3 (Table 4).

**Table 4:** Differential percentage between group A (ARTbrace) and Group B (old Lyon brace) at T1 (in-brace), T2 (6 months), T3 (1 year)

*Differential Percentage*	(%A-%B)/%B at T1	(%A-%B)/%B at T2	(%A-%B)/%B at T3
Differential Tho (*n* = 184)	0.37	0.417	0.715
Differential Lumb (*n* = 146)	0.301	0.672	0.317

**Fig. 5:**
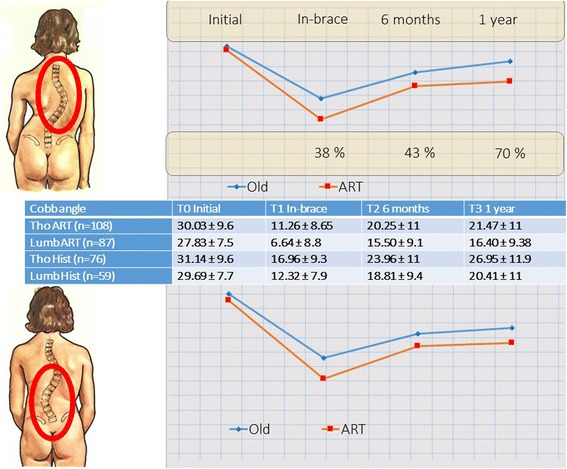
Evolution of Cobb’s angle average in the frontal plane during the follow up of 1 year. Between T0 and T1, we see both for lumbar and thoracic curves a large divergence. The new Lyon brace is much more corrective than the former plaster cast. This difference decreases when checking at 6 months (T2), but there is still a significant difference between the two groups. Between 6 months (T2) and one year (T3), there is again a divergence between the two curves, especially at the thoracic level, which means that the corrective effect continues and modifies the final result. A difference of 5° between the two groups is highly significant

The results were reported for the thoracic and lumbar curves. We find that the extra in-brace correction obtained persists at 6 months with even a tendency to improve after 1 year (Fig. 5).

With SPSS we can confirm with two tests: Shapiro-Wilk, and Kolmogorov-Smirnov, that the data comes from a normal distribution (Additional file 2).

We also use SPSS comparison of means tests to compare the two independent groups and answer the following questions (Additional file 3).

There was not a significant difference in the score of Thoracic Cobb angles before brace for control group with the old Lyon brace (M = 31.14, SD = 9.62) and the ARTbrace group (M = 30.03, SD = 8.30, t(182) = 0.834, *p* = 0.405.

There was not also a significant difference in the score of Lumbar Cobb before brace for control group with the old Lyon brace (M = 26.69, SD = 7.72) and the ARTbrace group (M = 27.82, SD = 7.51, t(144) = -0.884, *p* = 0.378.

There was a significant difference in the scores for thoracic and lumbar curves: in brace, at 6 months and after 1 year.

Thoracic in-brace: t(182) = 4.254, *p* = 0.00

Lumbar in-brace: t(144) = 3.993, *p* = 0.00

Thoracic at 6 months: t(182) = 2.284, *p* = 0.023

Lumbar at 6 months: t(144) = 2.131, *p* = 0.035

Thoracic at 1 year: t(182) = 3.205, *p* = 0.02

Lumbar at 1 year: t(134) = 2.463, *p* = 0.015

**Fig. 6:**
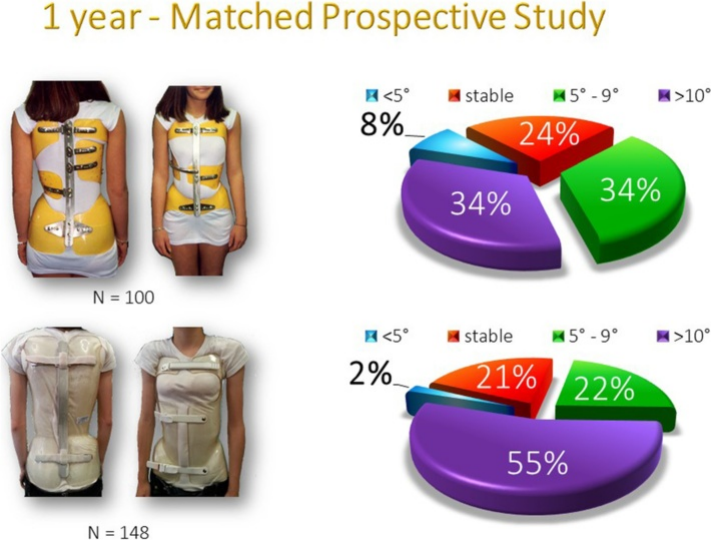
Angular distribution of improvements at 1 year. The one-year results were initially grouped according to the criteria of the SRS. But he scoliosis group whose angulation improved by more than 5° was too large with bad readability, so we have created a 4th group of angular correction of 10° or more. The 10° and more group is highly improved by the new Lyon brace

The distribution of improvements at the 1 year follow-up was first studied with standardized SRS criteria [30] with a worsening of more than 5°, stability at ± 5° and improvement of more than 5°. But it seems more pertinent to create a new class of improvement of 10° and more to improve the readability of the chart (Fig. 6).

#### Sagittal correction

The radiological follow-up of control group patients was performed without lateral X-ray and therefore it is not possible to make a statistical comparison. But thanks to the use of the micro dose EOS system a systematic sagittal analysis was possible for the main group of patients (ARTbrace).

In a previous study, we showed that the average thoracic kyphosis angle with the upper limit T4 was 37° [31]. For this study, we set the cut off at 30° for hypokyphosis or flat back.

The results are the subject of a separate presentation we can summarize.

**Fig. 7:**
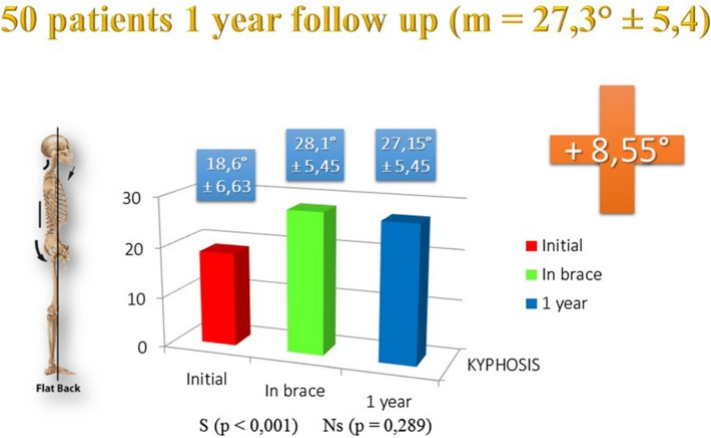
Improved initial flat back in-brace and at 1 year follow-up. Nearly half of scoliosis have a flat back with thoracic kyphosis angulation of less than 30°. In ARTbrace, back flat improved significantly of more than 8° and this improvement was maintained at 1 year follow-up without brace. There is no significant difference between the in-brace angle of thoracic kyphosis and at 1 year follow-up without brace

1. 73/148 patients (i.e. 49.4 %) had initial thoracic kyphosis below 30° (m = 19.6°, SD = 6.77)
2. In-brace angulation (m = 28.45°, SD = 5.84°) improvement in ARTbrace is 8.84°, significant (p = 0.000)
3. 50 patients were monitored with sagittal EOS without brace at the 1 year follow-up (m = 27.3°, SD = 5.40). For this specific group: initial kyphosis (m = 18.58°, SD = 6.63), in-brace kyphosis (m = 28.06°, SD = 5.45) and last follow-up without brace (m = 27.15°, SD = 5.45) were analysed (Fig. 7)

  The in-brace improvement rate is 50 % and very significant (p = 0.000), and without brace at the last follow up, the improvement rate is 46 % (p = 0.000). There was no statistical difference between the in-brace group and 1 year after when not wearing a brace (p = 0.289). The in-brace improvement is therefore maintained at the 1 year follow up when the brace is off.
  38 % of patients showed an improvement of 10° or more, 36 % of patients showed an improvement between 5° and 9°, 26 % of cases with stability, no back flat worsening.

##### Comparing plaster cast and ARTbrace

**Fig. 8:**
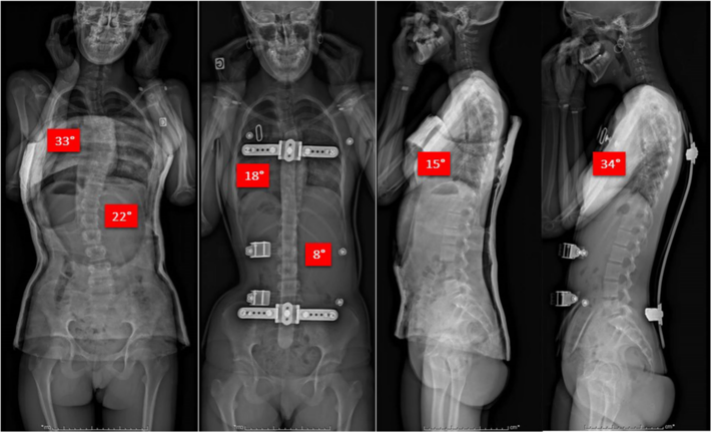
Comparing EDF plaster cast and ARTbrace. In the sagittal plane, the main difference with the EDF plaster cast and the former Lyon brace is that there are now two expansions in the sagittal plane. One at the sternoclavicular level for high thoracic kyphosis and the other at the abdominal level facilitating lumbar lordosis

Treatment was started with the EDF plaster cast and former Lyon brace. The translation along the vertical axis {Elongation) takes place to the detriment of the lumbar lordosis and the thoracic kyphosis. In ARTbrace, the sagittal plane is determined by the posterior metal bar and ahead, expansions at both ends allow active 4D correction (Fig. 8).

**Fig. 9:**
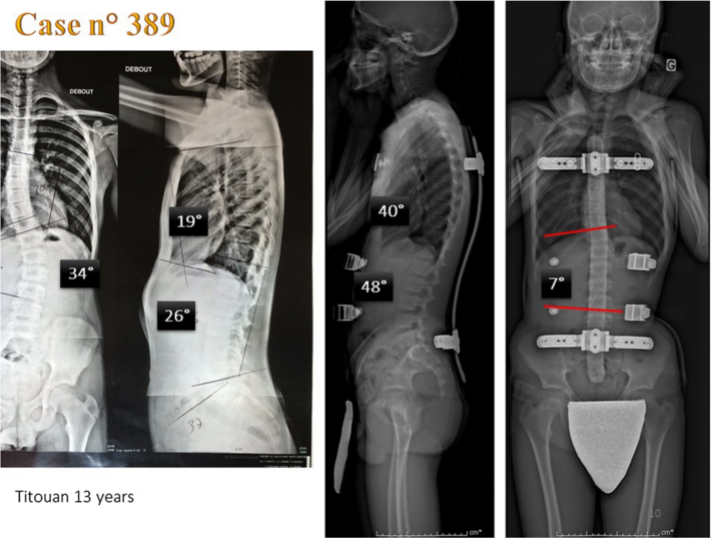
Case n° 389. Before bracing, there is an overall decrease in sagittal curvatures. In ARTbrace, it is possible to restore physiological sagittal curvatures

In this case n° 389, the improvement in the sagittal plane is important and harmonious. The spine follows the curve imposed by the posterior metallic bar (Fig. 9).

### Horizontal plane in-brace correction

**Fig. 10:**
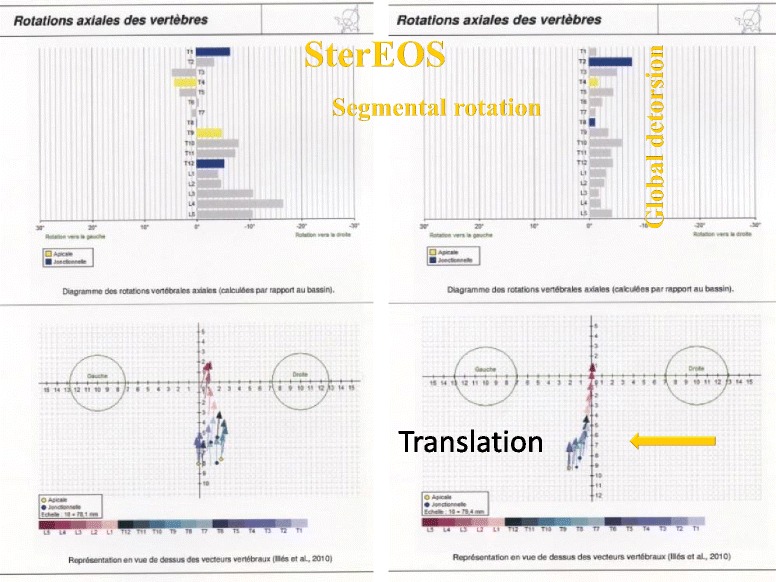
Improvement of segmental rotation and global detorsion. The sum of initial segmental rotations is 92° i.e. an average overall torsion of 5.4°. In-brace, the arithmetic sum (negative sign if the rotation is inverted) is 15° i.e. an overall torsion of less than 1°. The overall untwisting in this case exceeds 80 %. Qualitative vectorial representation confirms the translation of the vertebral bodies along the midline with overcorrection

Thanks to the use of the EOS system and reconstruction in 3D, in 15 characteristic cases, the effect of the ARTbrace in the horizontal plane could be studied. SterEOS gives us the position of each vertebra in the horizontal plane thanks to an upper view. The first visible effect of the brace is the translation of the spine towards the vertical axis due to the “mayonnaise tube” effect characteristic of high rigidity braces. The segmental rotation is automatically calculated and we define a global torsion index which is the average of all 17 segmental rotations (Fig. 10).

#### Case n° 401

**Fig. 11:**
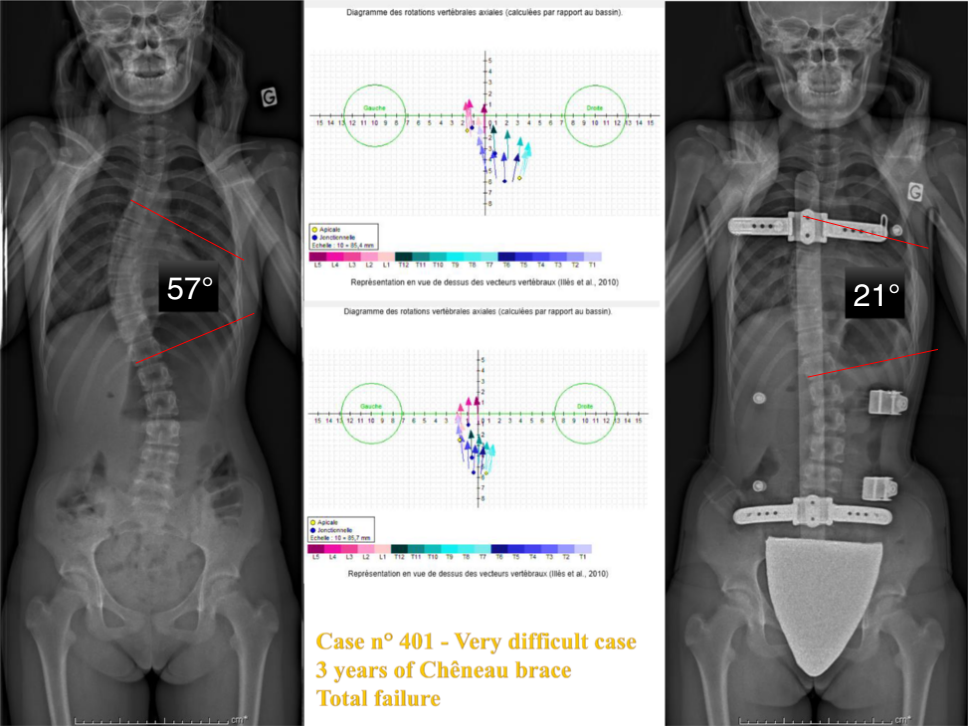
Case n° 401. In-brace correction after failure of 3 years Chêneau brace. Despite surgical indication, the parents refuse surgery and ask us to continue treatment with the new ARTbrace. The angular correction in the frontal plane remains above 50 %. The geometrical detorsion is satisfactory

This patient of 13 years has a very significant worsening of a thoracic scoliosis of 57° despite a Chêneau brace well executed and worn appropriately during three years. Parents wanted to wait before surgery and her surgeon asked us to try out the new Lyon brace on her. The immediate in-brace reduction obtained was 21°, and with sterEOS we see a good translation and reharmonisation of the spine (Fig. 11).

**Fig. 12:**
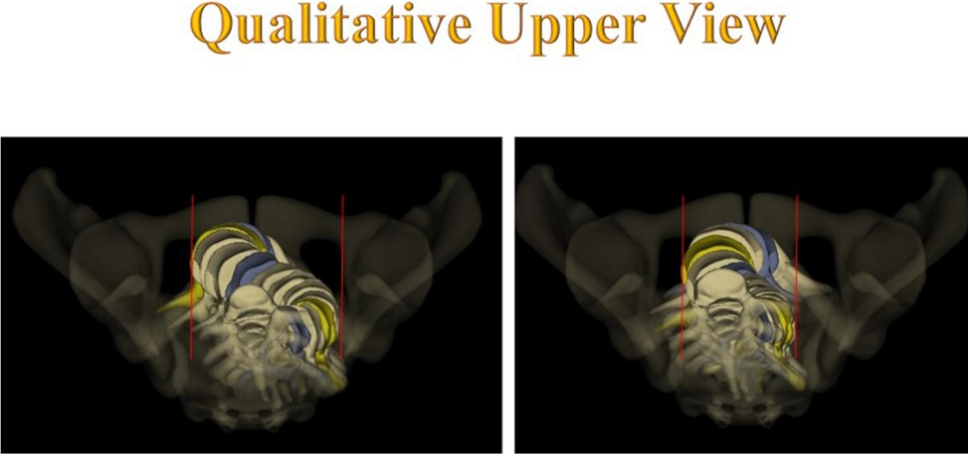
Da Vinci view of Case n° 401. The Da Vinci view confirms the geometric detorsion with recentering of the vertebral bodies along the midline and improved segmental derotation

The top view confirms the vertebral vector projection (Fig. 12).

**Fig. 13:**
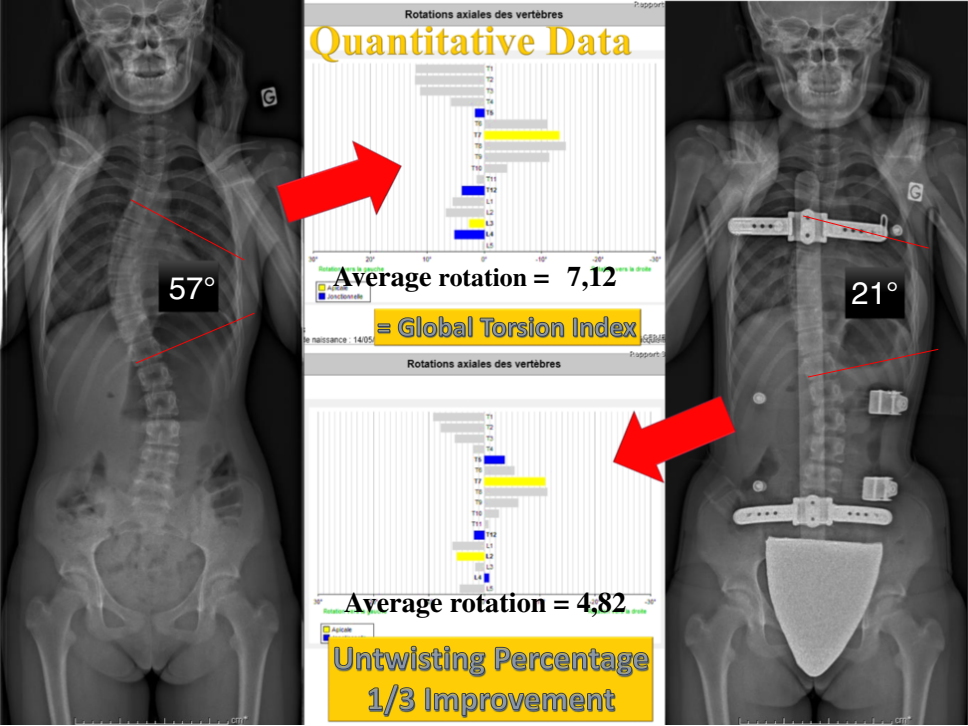
Global Torsion Index and percentage detorsion of case n° 401. The overall detorsion index has improved of more than one third in this case

The calculated overall untwisting which is the difference of both arithmetic average and all of the segmental rotations before and in-brace is 33 % (Fig. 13).

The average for the 15 patients studied was 37 %.

## Discussion

Adolescent idiopathic scoliosis (AIS) is a structural three-dimensional deformity of the spine arising in otherwise normal children during puberty. The use of brace in the conservative treatment for AIS [32] plays an important role and is meant to stop the evolution of the deformity in immature adolescents in order to prevent problems during adulthood. Different types of braces have been used in the treatment of AIS. The Lyon brace, created in 1947 by Pierre Stagnara, has been the first 3-points system adjustable brace, used after a plaster cast reduction [14, 16, 17].

Many previous studies support the positive results with the casting and Lyon braces [14, 16, 17]. The serial derotational plaster cast is commonly used for early onset scoliosis to create asymmetrical growth and remodelling [33]. The idea is to recreate, with a removable brace, the same derotational forces. In this study we report short time prospective results after 1 year of 148 scoliosis cases treated with the new Lyon ARTbrace, in correlation with a matched-pair control with the old Lyon Brace.

For ethical reasons, comparative studies of braces are exceptional. The originality of this study is the instantaneous transition from the old to the new Lyon brace in one step, for administrative reasons. Since May 2013 all patients (450) of JCdM at the ‘Clinique du Parc – Lyon’ were treated with the new Lyon brace (ARTbrace) instead of the classical EDF plaster cast. In fact from the first patient being treated with the new brace, the ARTbrace appeared to be a much more effective solution compared to the former plaster cast and it was even better tolerated. JCdM [7] has reported recently the early radiological results of 225 scoliosis cases treated with new Lyon brace (ARTbrace), matched with group control SRS & SOSORT criteria. This first immediate results have demonstrated that the in-brace correction of patients’ Cobb angle was 70 % with a correction 40 % higher than the former Lyon brace or historical Lyon brace and the other braces published in the literature, including retrospective studies. These results can be explained by new biomechanical concepts based on scoliosis detorsion such as:

- Segmental moulding with individual correction of the frontal and sagittal plane,
- Individual shape superposition
- Fixed sagittal plane with simultaneous correction of flat back or hyperkyphosis
- Night and day overcorrecting brace
- Axilla baby lift concept
- Coupled movement with biomechanical helicoidally detorsion of spine,
- High rigidity asymmetric polycarbonate with “mayonnaise tube” elongation effect
- Soft contact to increase tolerance and compliance
- 4D action with breathing toward the lateral expansions

One limit of this study was to only value the results of the immediate in-brace reduction of scoliotic curves by the ARTbrace, so in a limited time frame (3 day follow-up). In our study, instead, we report the early clinical and radiological results of group A (of 148 patients) with a follow-up after 1 year, so over a longer period of time, in correlation with a matched-pair control old Lyon brace group B.

In frontal radiological outcomes of this study, significant differences in the scores for Thoracic and Lumbar curves at T0, T1, T2, T3 are reported.

1. T1 initial in-brace correction
  For thoracic curves, the percentage improvement between the two groups was 37 %, confirming the initial results.
2. T2 at 6 months
  X-rays at 6 months are conducted without the brace. In both groups we observe an angular recurrence linked to the elasticity of the scoliotic curvature with and without brace, which is normal. Overall, this elasticity is statistically lower for group A, as if a better in-brace correction decreased elasticity of scoliosis i.e. the difference between in-brace angulation without brace. These results confirm the BRAIST study that retains the importance of in-brace correction brace as a fundamental criterion of the final outcome treatment. If we compare the two groups, the percentage of improvement is even slightly higher.
3. T3 after 1 year
  Many authors consider the results at 6 months as an excellent point in time to predict the final outcome [8, 34, 21, 35]. But above all, it is interesting to assess the evolution of the 2 curves that can be parallel, convergent or divergent, which has never been described to date. In our study, we find a divergence between both thoracic curves and lumbar curves in favor of group A, which would tend to prove that the initial efficiency of the brace is continuing with time. The average angular improvement is 5° between the two groups.

Certainly angular reduction after 1 year is not the final treatment outcome (2 years after weaning of brace), but we can identify some trends.

In the sagittal plane, this is the first brace to significantly improve the flat back and delordosis tendency.

This improvement with the ARTbrace can be related to segmental moulding with fixed sagittal correction but above all to the unscrewing or untwisting effect of the spine with translation of the vertebral bodies near the midline. In the literature, studies on improvement to the sagittal plane due to brace effect do not exist. Instead, many authors report accentuating brace effect on the flat back, probably related to axial stretching due to the overcorrection in the frontal plane [36, 37]. Analysing the effect of a brace on 38 patients treated with Chêneau using MR animation, shows a significant reduction of the mean Cobb angle of thoracic curves in-brace in MR animation coronal 0° projection (simulating A-P view in X-ray) but in -90° projection, simulating a lateral X-ray view, reported a reduction Kyphosis Cobb angle in 33/38 patients. So the MRI animation analysis confirms the straightening effect of the brace leading to the flattening of the sagittal spinal profile.

In the horizontal plane, this is the first time we no longer speak of segmental derotation but overall untwisting of scoliosis measured automatically by SterEOS (index of global detorsion).

As scoliosis is a structural three-dimensional deformity, the development of the EOS-system has allowed us to study better transverse plane analysis. It is a concept initiated by [38] his “torsiometer” is still considered the most accurate method of measurement of axial vertebral rotation on 2D A-P radiographs [39, 40]. Then MRI and CT have improved accuracy of vertebral rotations measurements but their clinical relevance is limited by the supine position of the patient for MRI and radiation exposition for CT. Today the development of the EOS-system has allowed us to study better transverse plane analysis and improve our knowledge. Courvoisier, analysing the transverse plane pattern of 111 patients with mild scoliosis in 3D by the EOS-system, combining apical axial rotation, the intervertebral axial rotation at junctions and the torsion index, has demonstrated that it is independent of the scoliotic curve location but above all significant in the determination of the progression risk of mild scoliosis [26].

### Simplicity of classification

The former Lyon brace requires the use of Lenke classification adapted to bracing. The segmental moulding of the new Lyon brace is much simpler and requires only two classes: C and S shaped scoliosis. The overall alignment is provided by the first moulding. For C shaped scoliosis, the thoracic and lumbar shifts will be realized in the same direction, while it will be carried out in opposite directions for the S curves. The lumbar oblique tilt and high curvatures are also much easier to manage.

### Overcorrection

The interest of overcorrection is not obvious especially for a specialist symmetrical brace such as the historical Lyon brace. However, we were using the night overcorrection for small thoracolumbar curves with good results. The Chêneau brace experience also goes in the direction of overcorrection. The ability to manually perform that overcorrection directly on the child is an advantage. Toru Maruyama showed us the interest of the shift in the scoliosis correction [41, 42] and the patient’s posture during the segmental moulding is very close to some of Schroth’s specific exercises [43].

### Lyon brace Management

So far we have not changed the overall management of the Lyon bracing treatment following the guidelines set by the SOSORT in 2011 [44].

- The indications are the same with wear time in the day depending on the initial angulation.
- The management is the same during treatment with adaptation of wearing time according to the elasticity of the scoliosis (X-ray without brace compared to in-brace X-Ray). Indeed, we believe that the spine is not made to grow properly under a rigid brace, and a very effective brace worn for a shorter period during the day is better than a less effective brace worn 23h/24. Although the consequences of the brace on bone mass is not obvious, the precautionary principle is required [45].
- Physiotherapy has remained the same, but the brace asymmetry and preparation for segmental moulding are closer to asymmetric methods and probably the current protocol will change in the future.
- The continuation of sports during treatment is a characteristic of the Lyon bracing treatment because the plaster cast causes a realignment of tension along the spine. The results with the historical Lyon brace were better when the children were practicing at least 5 h of sport each week. The initial full time wearing has the same creep effect. There is also another advantage with increase of skin tolerance (watch effect of Manuel Rigo). However, tolerance is worse in part time wearing.

### Compliance

Compliance is a key element of the final results of the treatment with immediate in-brace reduction. Compliance depends on the child and the family, but also on the brace which should be light, aesthetics and the allowance of normal breathing by expansions. The low dropout rate may also be due to changes in the brace.

## Conclusion

This study demonstrates not only good results of the ARTbrace about immediate in-brace reducibility of scoliosis described in previous studies published by the same author, but this trend is maintained further at 6 months and at 1 year. So the new concepts and first results of ARTbrace, defined as a modified or “new” Lyon brace, confirm that it can completely replace the casting and old Lyon brace process.

Finally, angular reduction at 1 year is not certainly the final treatment outcome (2 years after weaning of brace) even if some authors are using the reduction at this point in time as a predictive criterion. So, future studies could confirm if this criterion is valid and consequently the effectiveness of the ARTbrace.

## Availability of supporting data

The data set supporting the results and all SPSS statistics results are included within the article.

## Consent

All patients were informed and have given their consent for this work. This monocentric study was the object of a declaration to the CNIL under the number 1831534 in France and the procedure accepted.

## Additional files

Additional file 1:
**Excel worksheet using data from the prospective database.** (XLSX 139 kb) Additional file 2:
**SPSS v 20: descriptive statistics and distribution plot of all parameters. **(PDF 145 kb) Additional file 3:
**SPSS v 20: t-test comparing means of old Lyon and new Lyon ARTbrace.** (PDF 398 kb)

## Abreviations

AIS: Adolescent idiopathic scoliosis
RH: Rib hump
BU: Bunnel’s Angle of Trunk Rotation
SRS: Scoliosis research society
SOSORT: Society on scoliosis orthopedic and rehabilitation treatment
