# Supplementary material for: The new Lyon ARTbrace versus the historical Lyon brace: a prospective case series of 148 consecutive scoliosis with short time results after 1 year compared with a historical retrospective case series of 100 consecutive scoliosis; SOSORT award 2015 winner
Source: Scoliosis. 2015 Aug 19;10:26. doi: 10.1186/s13013-015-0047-6 (PMC4545553; doi:10.1186/s13013-015-0047-6)
Supplement: Additional file 2: — SPSS v 20: descriptive statistics and distribution plot of all parameters. (PDF 145 kb) [file 13013_2015_47_MOESM2_ESM.pdf]

```
GET
  FILE='D:\Bibliothèques\Documents\ARTbrace matched pair control.sav'.
DATASET NAME Ensemble_de_données1 WINDOW=FRONT.
EXAMINE VARIABLES=VAR00005 VAR00008 VAR00009 VAR00010 VAR00013 VAR00014 VA
R00017 VAR00018 BY VAR00021
  /PLOT BOXPLOT STEMLEAF NPPLOT
  /COMPARE GROUPS
  /STATISTICS DESCRIPTIVES
  /CINTERVAL 95
  /MISSING LISTWISE
  /NOTOTAL.
```

Explore

```
[Ensemble_de_données1] D:\Bibliothèques\Documents\ARTbrace matched pair co
ntrol.sav
```

Categorie

Case Processing Summary

		Cases					
		Valid		Missing		Total	
	Categorie	N	Percent	N	Percent	N	Percent
T init	1,00	36	36,4%	63	63,6%	99	100,0%
	2,00	46	16,0%	242	84,0%	288	100,0%
L init	1,00	36	36,4%	63	63,6%	99	100,0%
	2,00	46	16,0%	242	84,0%	288	100,0%
T in-brace	1,00	36	36,4%	63	63,6%	99	100,0%
	2,00	46	16,0%	242	84,0%	288	100,0%
Lin-brace	1,00	36	36,4%	63	63,6%	99	100,0%
	2,00	46	16,0%	242	84,0%	288	100,0%
T 6 months	1,00	36	36,4%	63	63,6%	99	100,0%
	2,00	46	16,0%	242	84,0%	288	100,0%
L 6 months	1,00	36	36,4%	63	63,6%	99	100,0%
	2,00	46	16,0%	242	84,0%	288	100,0%
T 1 year	1,00	36	36,4%	63	63,6%	99	100,0%
	2,00	46	16,0%	242	84,0%	288	100,0%
L 1 year	1,00	36	36,4%	63	63,6%	99	100,0%
	2,00	46	16,0%	242	84,0%	288	100,0%

Descriptives

Categorie				Statistic	Std. Error
T init	1,00	Mean		31,1111	1,53260
		95% Confidence Interval for Mean	Lower Bound	27,9998	
			Upper Bound	34,2224	
		5% Trimmed Mean		30,5556	
		Median		30,0000	
		Variance		84,559	
		Std. Deviation		9,19558	
		Minimum		20,00	
		Maximum		54,00	
		Range		34,00	
		Interquartile Range		12,50	
		Skewness		,770	,393
		Kurtosis		-,157	,768
	2,00	Mean		28,9348	1,09562
		95% Confidence Interval for Mean	Lower Bound	26,7281	
			Upper Bound	31,1415	
		5% Trimmed Mean		28,4155	
		Median		27,0000	
		Variance		55,218	
		Std. Deviation		7,43087	
		Minimum		19,00	
		Maximum		50,00	
		Range		31,00	
		Interquartile Range		10,25	
		Skewness		,951	,350
		Kurtosis		,545	,688
L init	1,00	Mean		26,1389	1,17186
		95% Confidence Interval for Mean	Lower Bound	23,7599	
			Upper Bound	28,5179	
		5% Trimmed Mean		25,7531	
		Median		24,5000	
		Variance		49,437	
		Std. Deviation		7,03117	
		Minimum		16,00	
		Maximum		45,00	
		Range		29,00	
		Interquartile Range		9,00	
		Skewness		1,021	,393
		Kurtosis		,468	,768
	2,00	Mean		29,1087	1,22740
		95% Confidence Interval for Mean	Lower Bound	26,6366	
			Upper Bound	31,5808	
		5% Trimmed Mean		28,6256	

Descriptives

Categorie				Statistic	Std. Error
		Median		28,0000	
		Variance		69,299	
		Std. Deviation		8,32460	
		Minimum		16,00	
		Maximum		53,00	
		Range		37,00	
		Interquartile Range		11,00	
		Skewness		,857	,350
		Kurtosis		,700	,688
T in-brace	1,00	Mean		18,4444	1,54172
		95% Confidence Interval for Mean	Lower Bound	15,3146	
			Upper Bound	21,5743	
		5% Trimmed Mean		17,8457	
		Median		18,0000	
		Variance		85,568	
		Std. Deviation		9,25031	
		Minimum		4,00	
		Maximum		49,00	
		Range		45,00	
		Interquartile Range		8,50	
		Skewness		1,206	,393
		Kurtosis		2,280	,768
	2,00	Mean		12,3043	1,18520
		95% Confidence Interval for Mean	Lower Bound	9,9172	
			Upper Bound	14,6915	
		5% Trimmed Mean		11,7319	
		Median		11,0000	
		Variance		64,616	
		Std. Deviation		8,03843	
		Minimum		,00	
		Maximum		34,00	
		Range		34,00	
		Interquartile Range		12,00	
		Skewness		,963	,350
		Kurtosis		,731	,688
Lin-brace	1,00	Mean		14,0556	1,18853
		95% Confidence Interval for Mean	Lower Bound	11,6427	
			Upper Bound	16,4684	
		5% Trimmed Mean		13,5741	
		Median		13,5000	
		Variance		50,854	
		Std. Deviation		7,13120	
		Minimum		4,00	

Descriptives

Categorie				Statistic	Std. Error
		Maximum		35,00	
		Range		31,00	
		Interquartile Range		10,50	
		Skewness		,894	,393
		Kurtosis		,934	,768
	2,00	Mean		10,8478	1,22098
		95% Confidence Interval for Mean	Lower Bound	8,3886	
			Upper Bound	13,3070	
		5% Trimmed Mean		10,4952	
		Median		9,0000	
		Variance		68,576	
		Std. Deviation		8,28108	
		Minimum		,00	
		Maximum		30,00	
		Range		30,00	
		Interquartile Range		12,25	
		Skewness		,567	,350
		Kurtosis		-,699	,688
T 6 months	1,00	Mean		25,4444	1,84711
		95% Confidence Interval for Mean	Lower Bound	21,6946	
			Upper Bound	29,1943	
		5% Trimmed Mean		24,7346	
		Median		24,0000	
		Variance		122,825	
		Std. Deviation		11,08266	
		Minimum		10,00	
		Maximum		55,00	
		Range		45,00	
		Interquartile Range		11,50	
		Skewness		1,152	,393
		Kurtosis		1,290	,768
	2,00	Mean		21,2826	1,58698
		95% Confidence Interval for Mean	Lower Bound	18,0863	
			Upper Bound	24,4790	
		5% Trimmed Mean		20,7319	
		Median		19,0000	
		Variance		115,852	
		Std. Deviation		10,76344	
		Minimum		2,00	
		Maximum		52,00	
		Range		50,00	
		Interquartile Range		14,25	
		Skewness		,911	,350

Descriptives

Categorie				Statistic	Std. Error
		Kurtosis		,679	,688
L 6 months	1,00	Mean		20,2222	1,38307
		95% Confidence Interval for Mean	Lower Bound	17,4144	
			Upper Bound	23,0300	
		5% Trimmed Mean		19,5926	
		Median		19,0000	
		Variance		68,863	
		Std. Deviation		8,29840	
		Minimum		9,00	
		Maximum		45,00	
		Range		36,00	
		Interquartile Range		9,50	
		Skewness		1,079	,393
		Kurtosis		1,466	,768
	2,00	Mean		19,3913	1,32915
		95% Confidence Interval for Mean	Lower Bound	16,7143	
			Upper Bound	22,0684	
		5% Trimmed Mean		19,0942	
		Median		18,0000	
		Variance		81,266	
		Std. Deviation		9,01475	
		Minimum		3,00	
		Maximum		40,00	
		Range		37,00	
		Interquartile Range		12,50	
		Skewness		,605	,350
		Kurtosis		-,354	,688
T 1 year	1,00	Mean		28,2222	1,88735
		95% Confidence Interval for Mean	Lower Bound	24,3907	
			Upper Bound	32,0537	
		5% Trimmed Mean		27,5247	
		Median		27,0000	
		Variance		128,235	
		Std. Deviation		11,32409	
		Minimum		14,00	
		Maximum		55,00	
		Range		41,00	
		Interquartile Range		13,25	
		Skewness		,979	,393
		Kurtosis		,215	,768
	2,00	Mean		22,1957	1,41647
		95% Confidence Interval for Mean	Lower Bound	19,3427	
			Upper Bound	25,0486	

Descriptives

Categorie				Statistic	Std. Error
		5% Trimmed Mean		22,0097	
		Median		20,0000	
		Variance		92,294	
		Std. Deviation		9,60699	
		Minimum		5,00	
		Maximum		43,00	
		Range		38,00	
		Interquartile Range		13,75	
		Skewness		,346	,350
		Kurtosis		-,491	,688
L 1 year	1,00	Mean		21,9167	1,38379
		95% Confidence Interval for Mean	Lower Bound	19,1074	
			Upper Bound	24,7259	
		5% Trimmed Mean		21,1543	
		Median		20,5000	
		Variance		68,936	
		Std. Deviation		8,30275	
		Minimum		10,00	
		Maximum		48,00	
		Range		38,00	
		Interquartile Range		10,50	
		Skewness		1,411	,393
		Kurtosis		2,674	,768
	2,00	Mean		20,2174	1,35487
		95% Confidence Interval for Mean	Lower Bound	17,4885	
			Upper Bound	22,9462	
		5% Trimmed Mean		20,0266	
		Median		18,0000	
		Variance		84,441	
		Std. Deviation		9,18916	
		Minimum		2,00	
		Maximum		40,00	
		Range		38,00	
		Interquartile Range		13,25	
		Skewness		,443	,350
		Kurtosis		-,472	,688

Tests of Normality

	Categorie	Kolmogorov-Smirnov[a]			Shapiro-Wilk		
		Statistic	df	Sig.	Statistic	df	Sig.
T init	1,00	,128	36	,143	,923	36	,016
	2,00	,159	46	,005	,921	46	,004
L init	1,00	,175	36	,007	,907	36	,006
	2,00	,131	46	,045	,941	46	,022
T in-brace	1,00	,183	36	,004	,920	36	,013
	2,00	,138	46	,029	,918	46	,003
Lin-brace	1,00	,114	36	,200*	,938	36	,044
	2,00	,113	46	,180	,934	46	,012
T 6 months	1,00	,131	36	,122	,902	36	,004
	2,00	,163	46	,004	,936	46	,014
L 6 months	1,00	,119	36	,200*	,926	36	,019
	2,00	,148	46	,013	,950	46	,046
T 1 year	1,00	,153	36	,032	,900	36	,003
	2,00	,139	46	,027	,965	46	,183
L 1 year	1,00	,123	36	,185	,889	36	,002
	2,00	,140	46	,025	,958	46	,095

*. This is a lower bound of the true significance.

a. Lilliefors Significance Correction

T init

Stem-and-Leaf Plots

```
T init Stem-and-Leaf Plot for
VAR00021= 1,00

 Frequency    Stem &  Leaf

    10,00        2 .  0000112334
     6,00        2 .  555688
    10,00        3 .  0000222234
     2,00        3 .  56
     4,00        4 .  0134
     2,00        4 .  56
     1,00        5 .  0
     1,00 Extremes    (>=54)

 Stem width:     10,00
 Each leaf:       1 case(s)
```

```
T init Stem-and-Leaf Plot for
VAR00021= 2,00

 Frequency    Stem &  Leaf

     1,00        1 .  9
     6,00        2 .  000000
     2,00        2 .  22
     9,00        2 .  444455555
     6,00        2 .  667777
     5,00        2 .  88889
     3,00        3 .  000
     2,00        3 .  22
     4,00        3 .  4555
     1,00        3 .  7
     3,00        3 .  889
      ,00        4 .
     2,00        4 .  22
      ,00        4 .
     1,00        4 .  7
     1,00 Extremes    (>=50)

 Stem width:     10,00
 Each leaf:       1 case(s)
```

Normal Q-Q Plots

Normal Q-Q Plot of T init

for VAR00021= 1,00

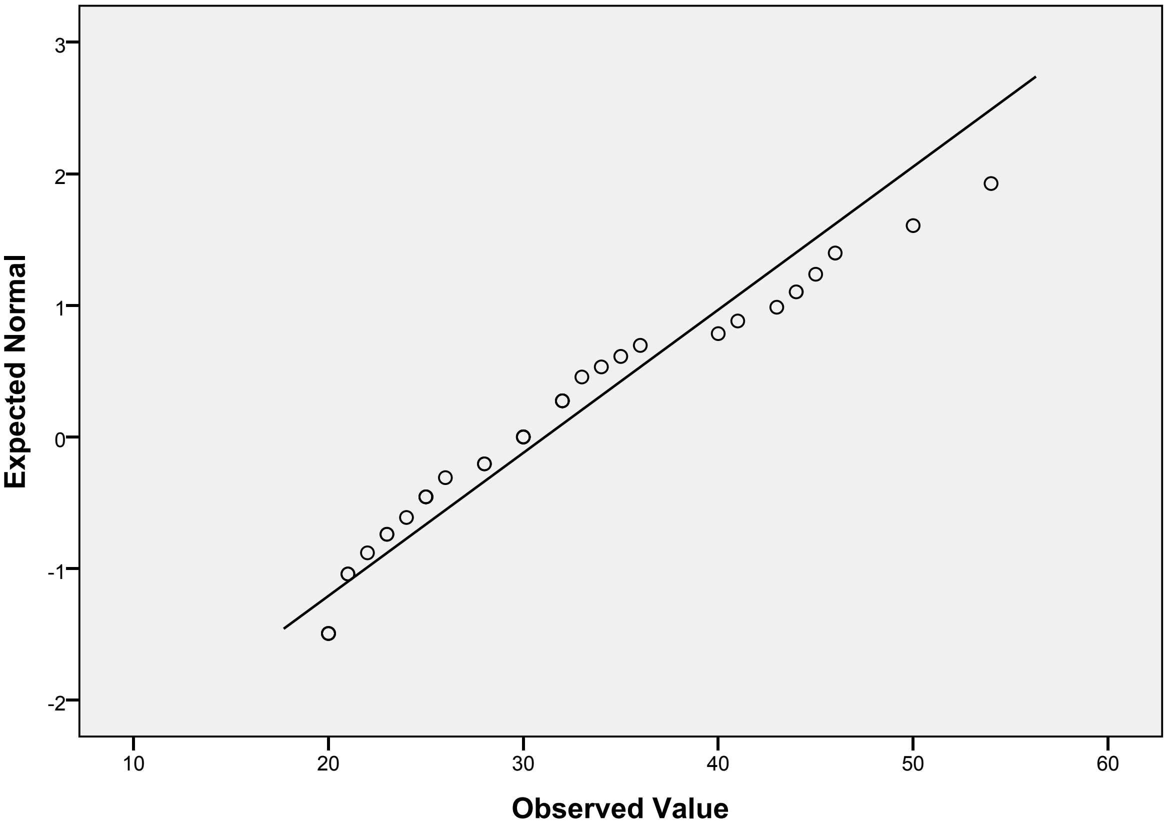

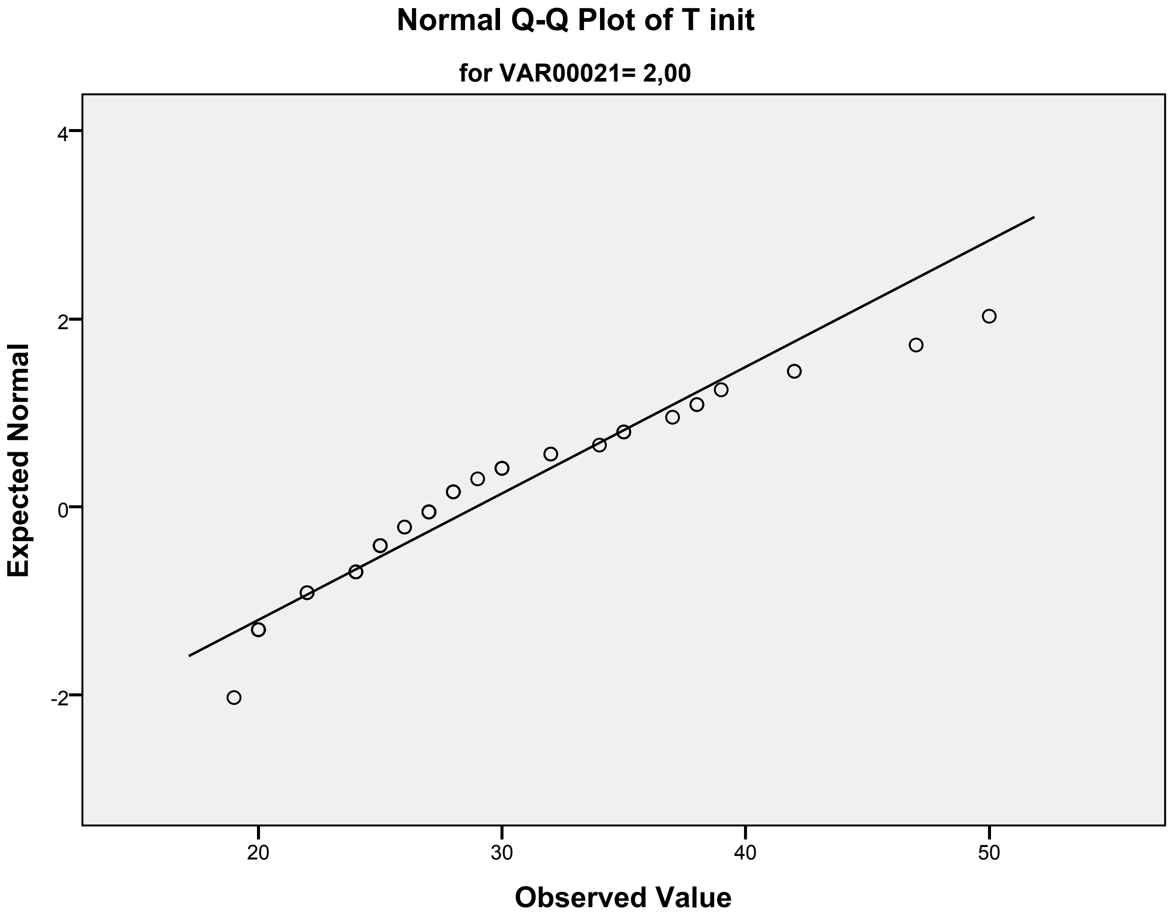

Detrended Normal Q-Q Plots

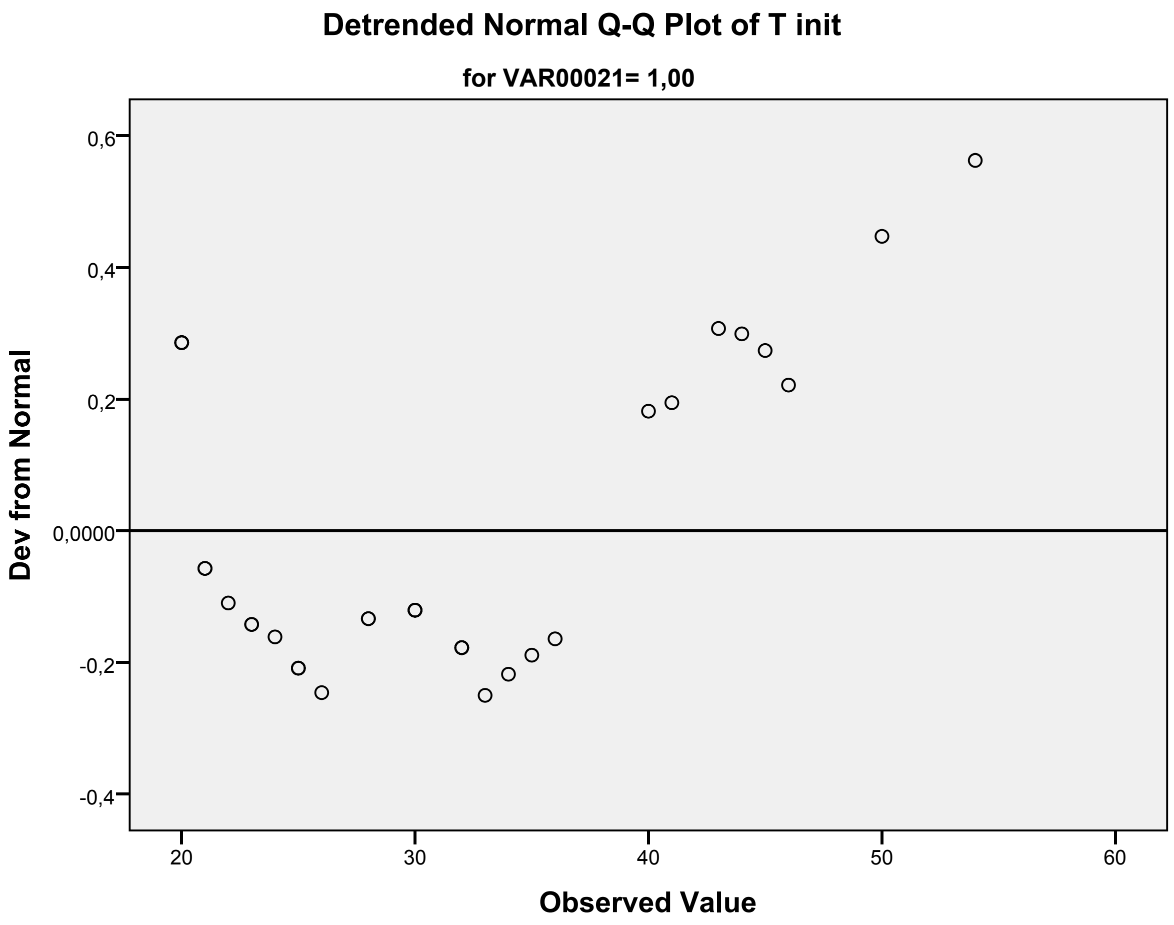
Detrended Normal Q-Q Plot of T init
for VAR00021= 1,00
Dev from Normal
0,6
0,4
0,2
0,0000
-0,2
-0,4
20
30
40
50
60
Observed Value

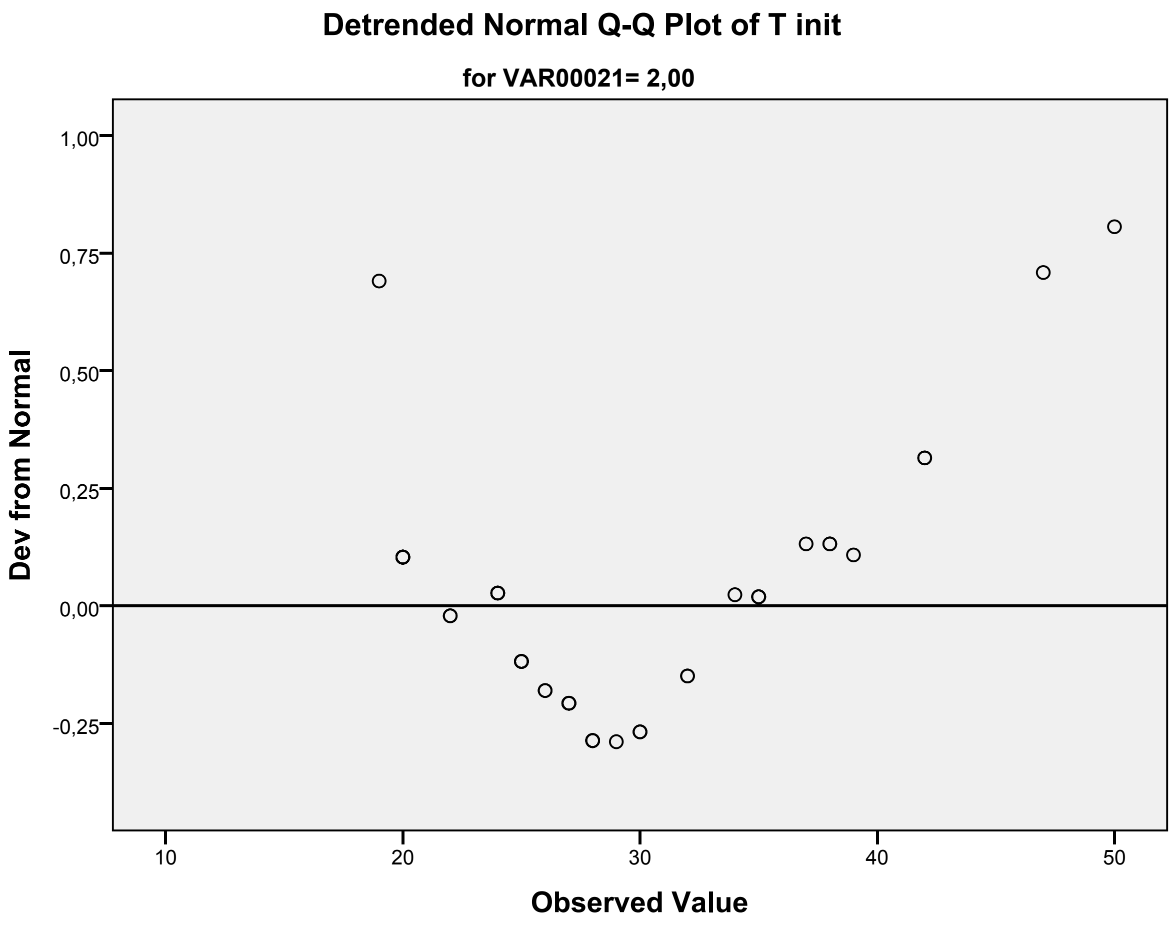
Detrended Normal Q-Q Plot of T init
for VAR00021= 2,00
Dev from Normal
1,00
0,75
0,50
0,25
0,00
-0,25
10
20
30
40
50
Observed Value

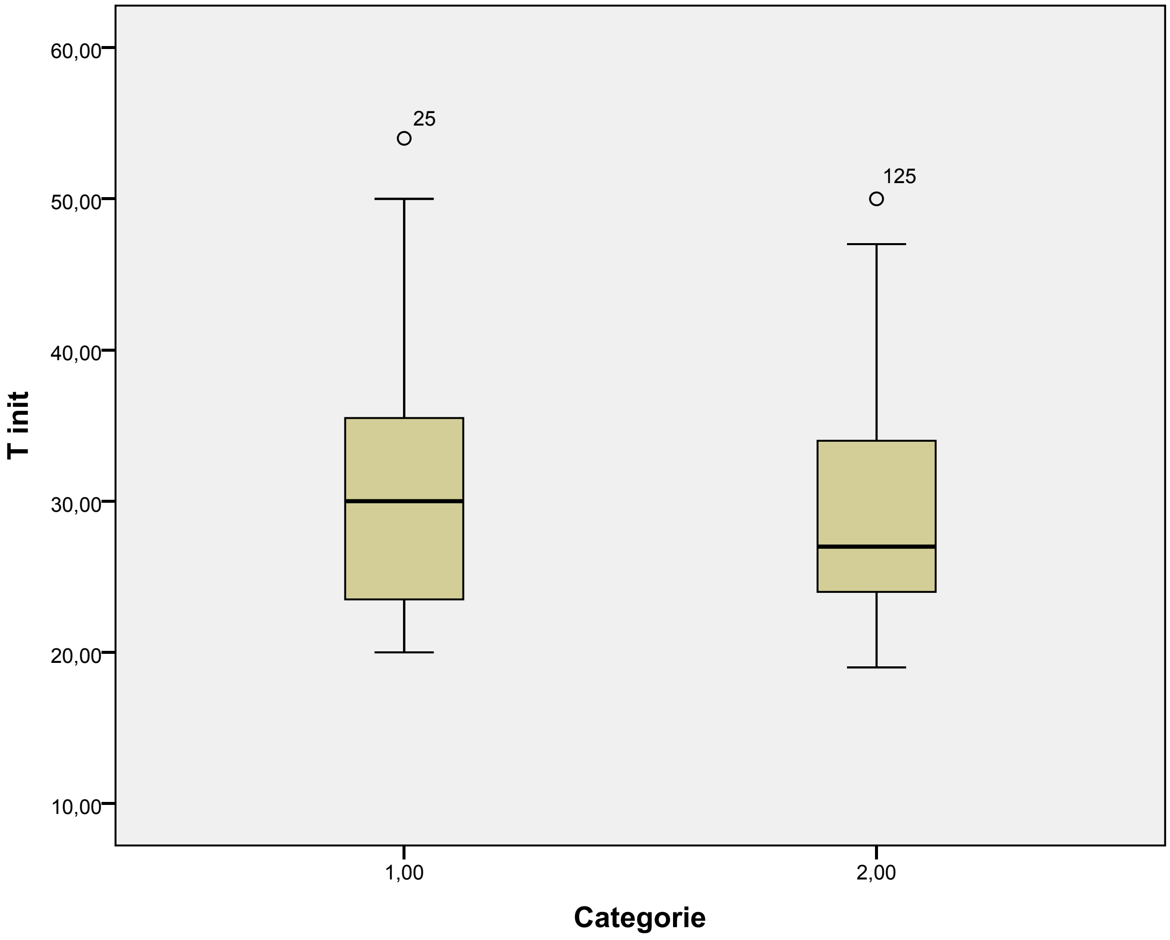

L init

Stem-and-Leaf Plots

```
L init Stem-and-Leaf Plot for
VAR00021= 1,00

 Frequency    Stem &  Leaf

     3,00        1 .  678
    15,00        2 .  000011122233334
     8,00        2 .  55556679
     6,00        3 .  003334
     1,00        3 .  9
     2,00        4 .  00
     1,00 Extremes    (>=45)

 Stem width:     10,00
 Each leaf:       1 case(s)
```

```
L init Stem-and-Leaf Plot for
VAR00021= 2,00

 Frequency    Stem &  Leaf

      ,00        1 .
     1,00        1 .  6
     4,00        1 .  8889
     5,00        2 .  11111
     3,00        2 .  222
     3,00        2 .  555
     5,00        2 .  77777
     4,00        2 .  8889
     6,00        3 .  000000
     5,00        3 .  22233
     2,00        3 .  55
     2,00        3 .  67
     1,00        3 .  8
      ,00        4 .
     1,00        4 .  2
     2,00        4 .  55
      ,00        4 .
     1,00        4 .  8
     1,00 Extremes    (>=53)

 Stem width:     10,00
 Each leaf:       1 case(s)
```

Normal Q-Q Plots

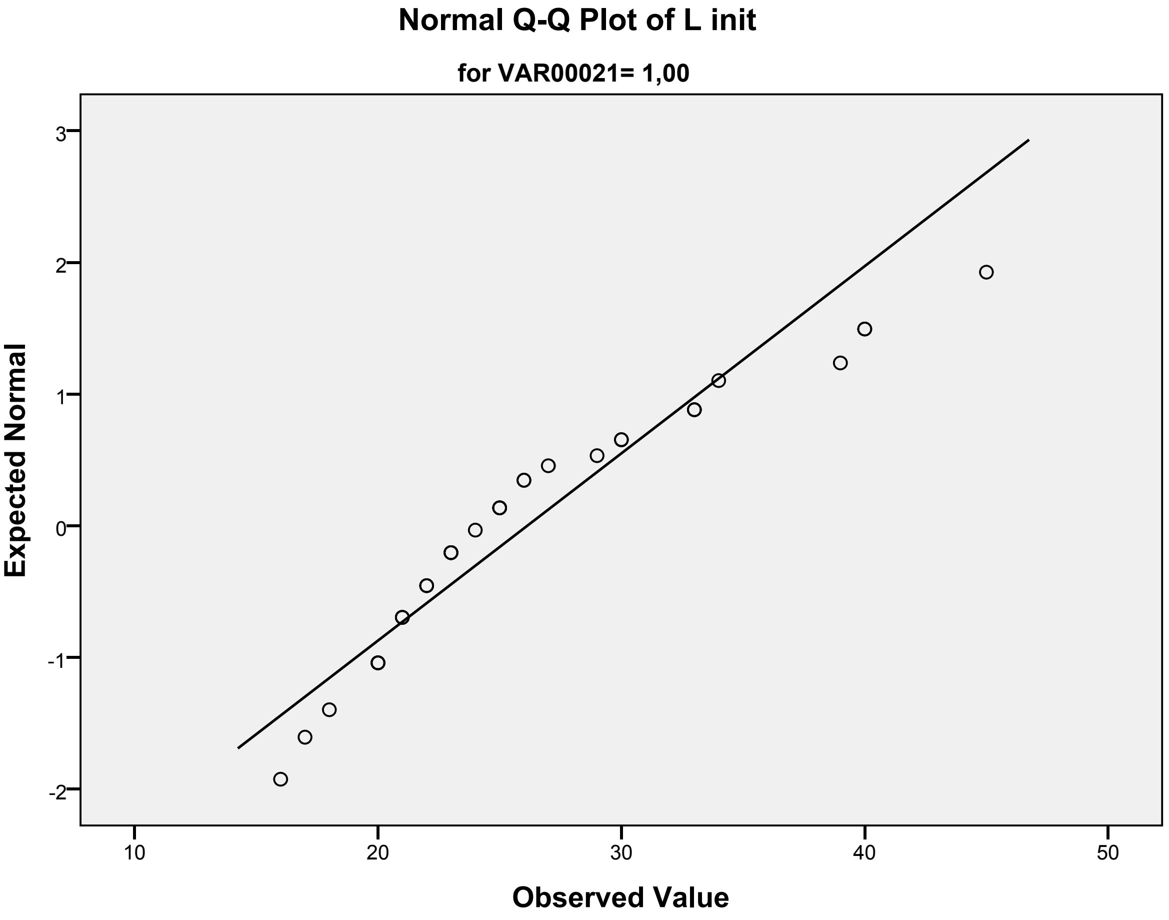
Normal Q-Q Plot of L init
for VAR00021= 1,00
Expected Normal
Observed Value
3
2
1
0
-1
-2
10
20
30
40
50

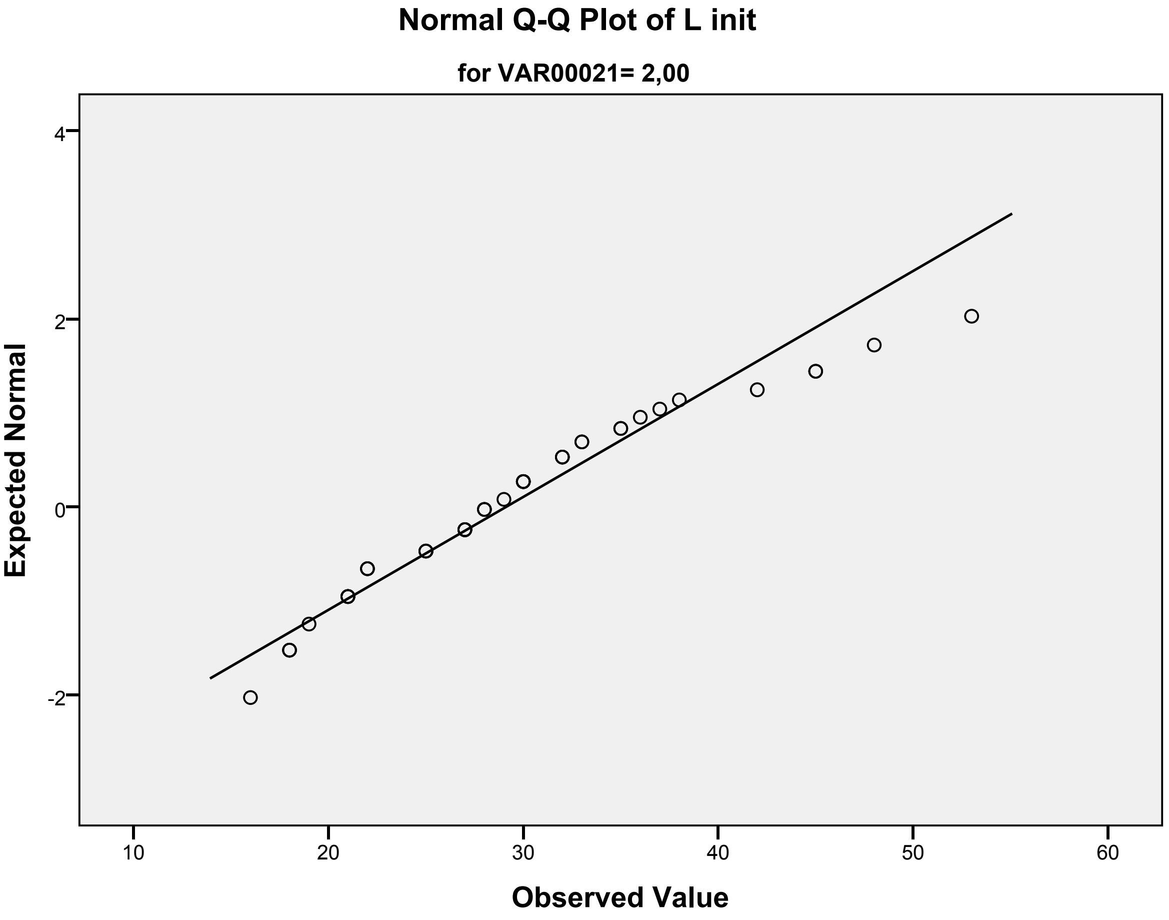

Detrended Normal Q-Q Plots

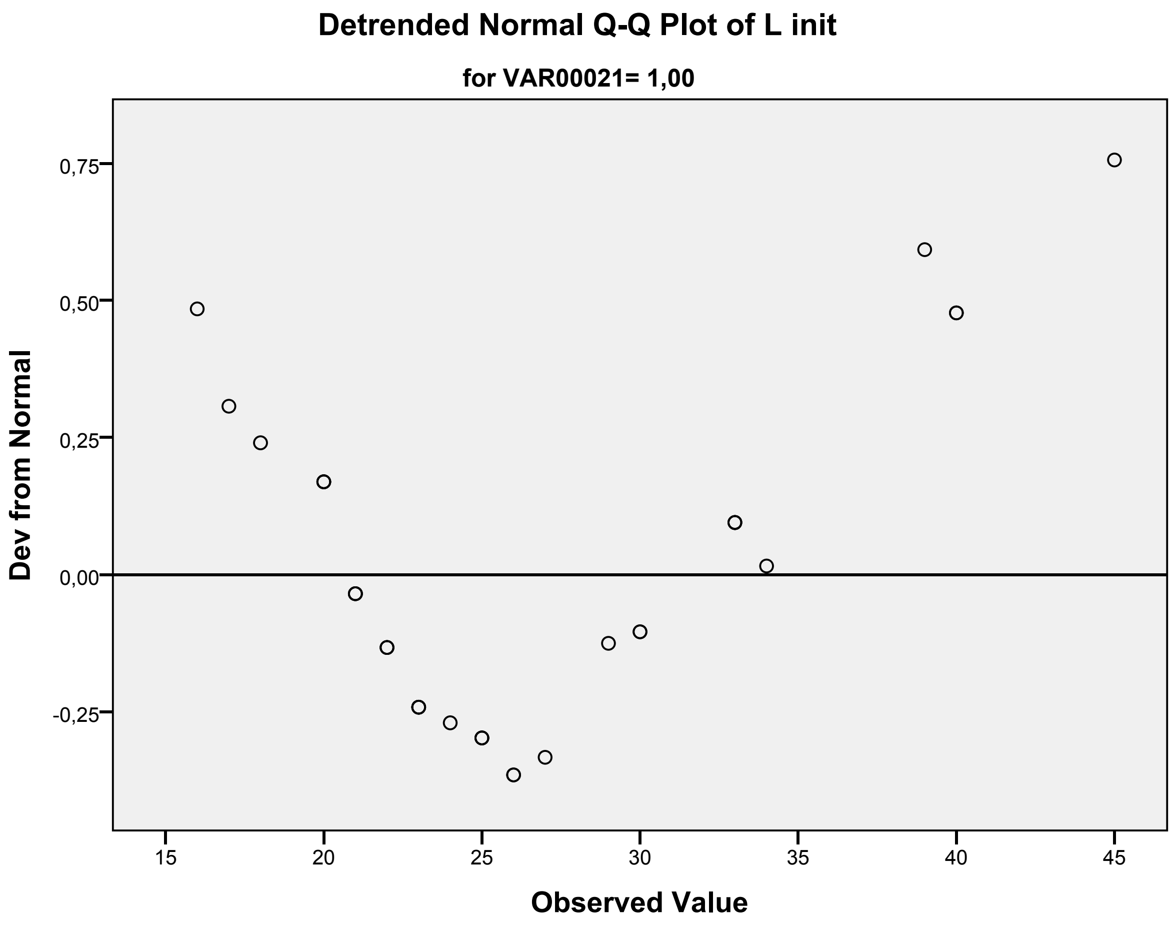

Detrended Normal Q-Q Plot of L init
for VAR00021= 1,00
Dev from Normal
0,75
0,50
0,25
0,00
-0,25
15
20
25
30
35
40
45
Observed Value

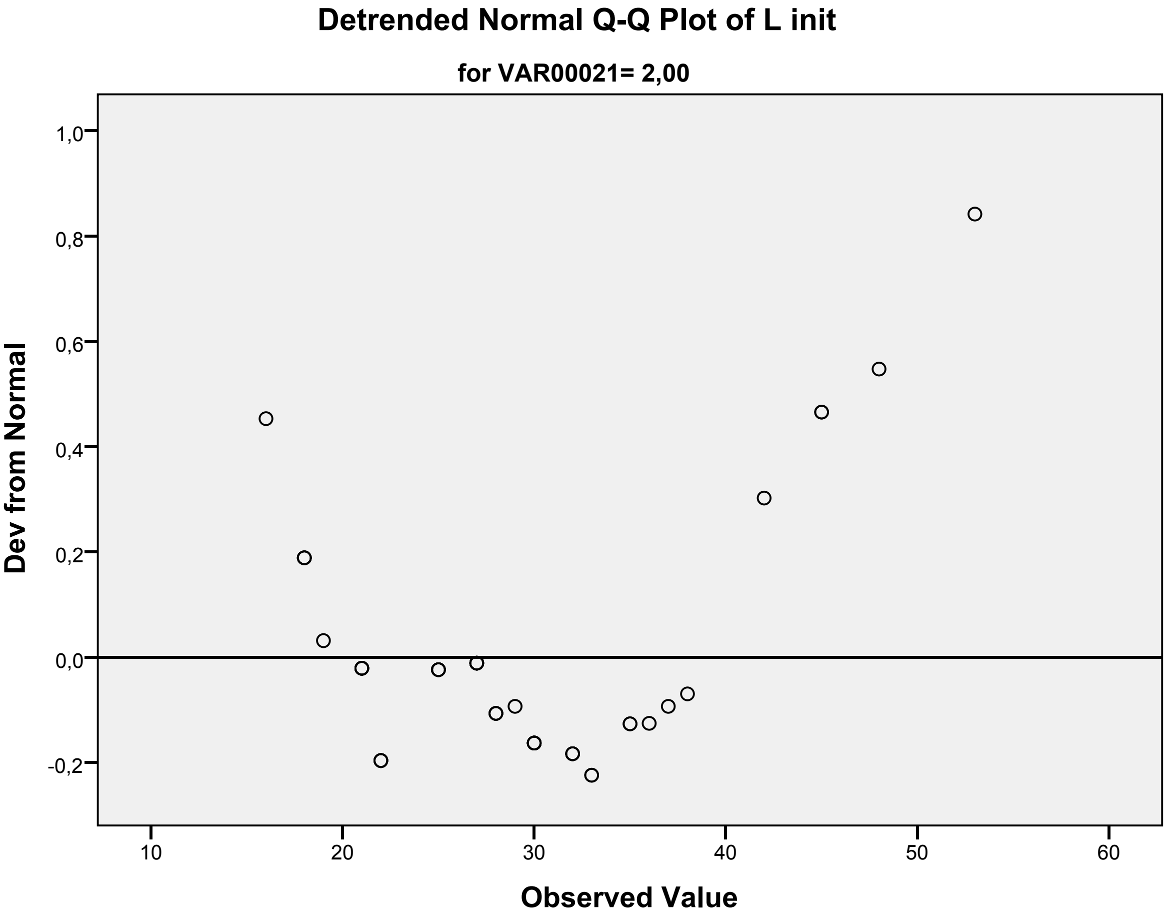

Detrended Normal Q-Q Plot of L init
for VAR00021= 2,00
Dev from Normal
1,0
0,8
0,6
0,4
0,2
0,0
-0,2
10
20
30
40
50
60
Observed Value

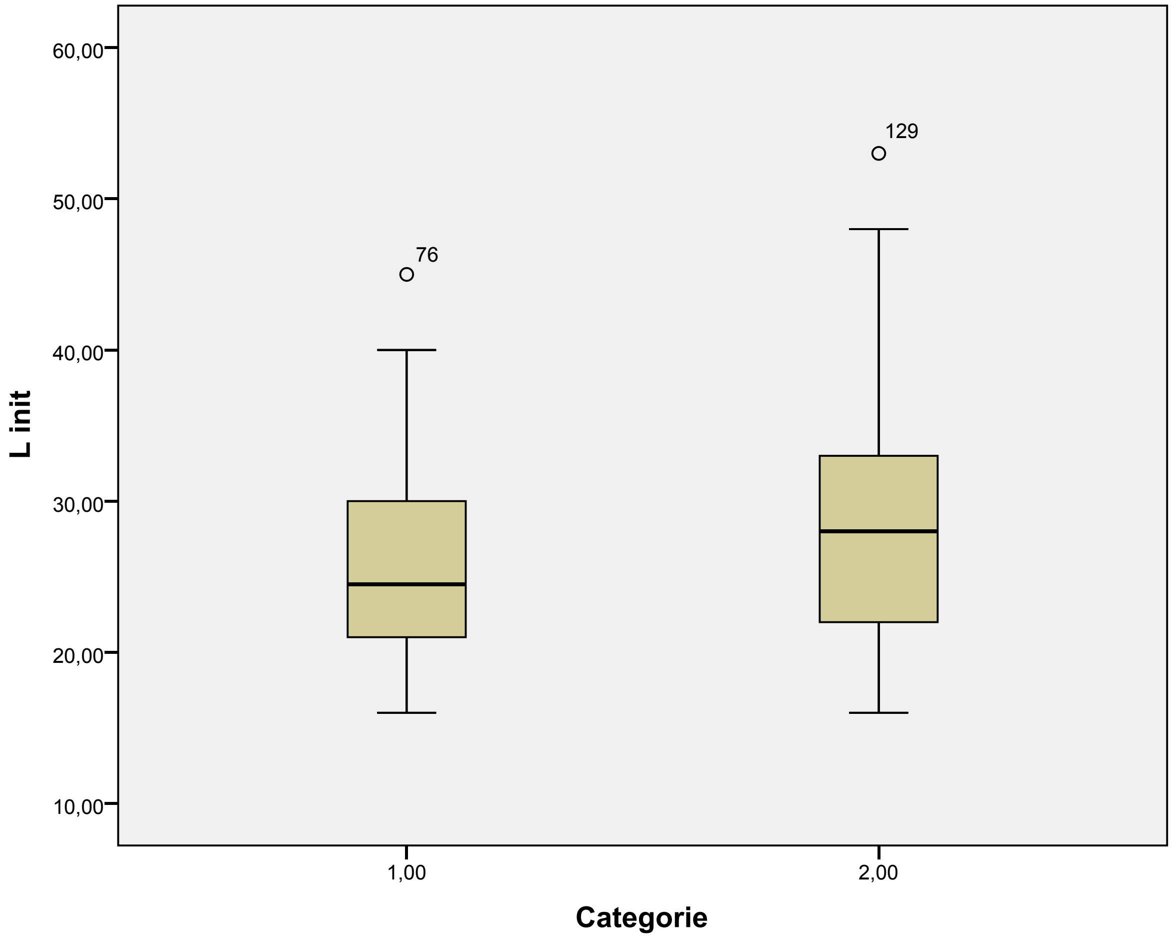

T in-brace

Stem-and-Leaf Plots

```
T in-brace Stem-and-Leaf Plot for
VAR00021= 1,00

 Frequency    Stem &  Leaf

     1,00        0 .  4
     3,00        0 .  578
     8,00        1 .  00113334
    13,00        1 .  5556788888999
     3,00        2 .  002
     4,00        2 .  5788
     1,00        3 .  1
     3,00 Extremes    (>=34)

 Stem width:     10,00
 Each leaf:       1 case(s)
```

```
T in-brace Stem-and-Leaf Plot for
VAR00021= 2,00

 Frequency    Stem &  Leaf

     1,00        0 .  0
     3,00        0 .  333
     6,00        0 .  444445
     3,00        0 .  667
     9,00        0 .  888888999
     2,00        1 .  11
     4,00        1 .  2222
     3,00        1 .  444
     1,00        1 .  6
     9,00        1 .  888889999
     1,00        2 .  1
      ,00        2 .
      ,00        2 .
     1,00        2 .  7
     1,00        2 .  8
      ,00        3 .
      ,00        3 .
     2,00        3 .  44

 Stem width:     10,00
 Each leaf:       1 case(s)
```

Normal Q-Q Plots

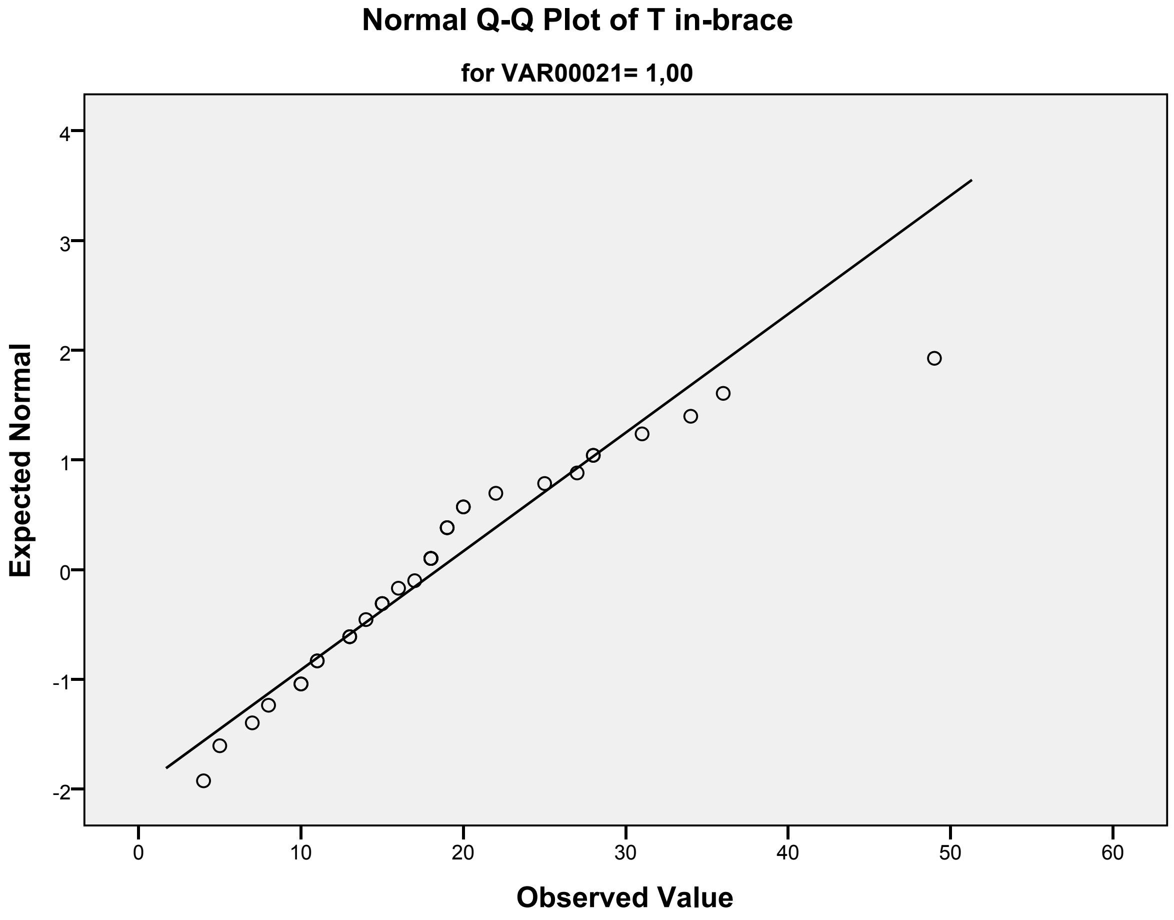
Normal Q-Q Plot of T in-brace
for VAR00021= 1,00
Expected Normal
Observed Value
4
3
2
1
0
-1
-2
0
10
20
30
40
50
60

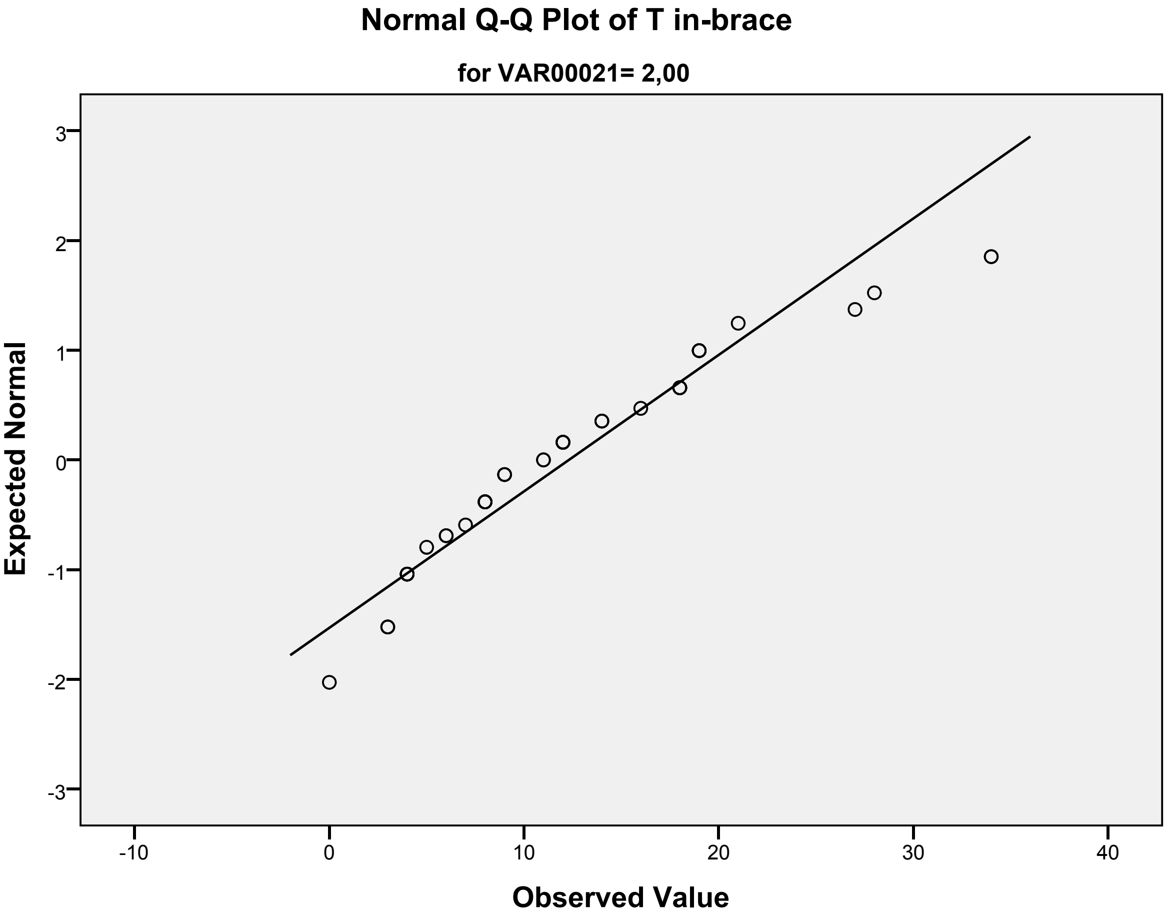

Detrended Normal Q-Q Plots

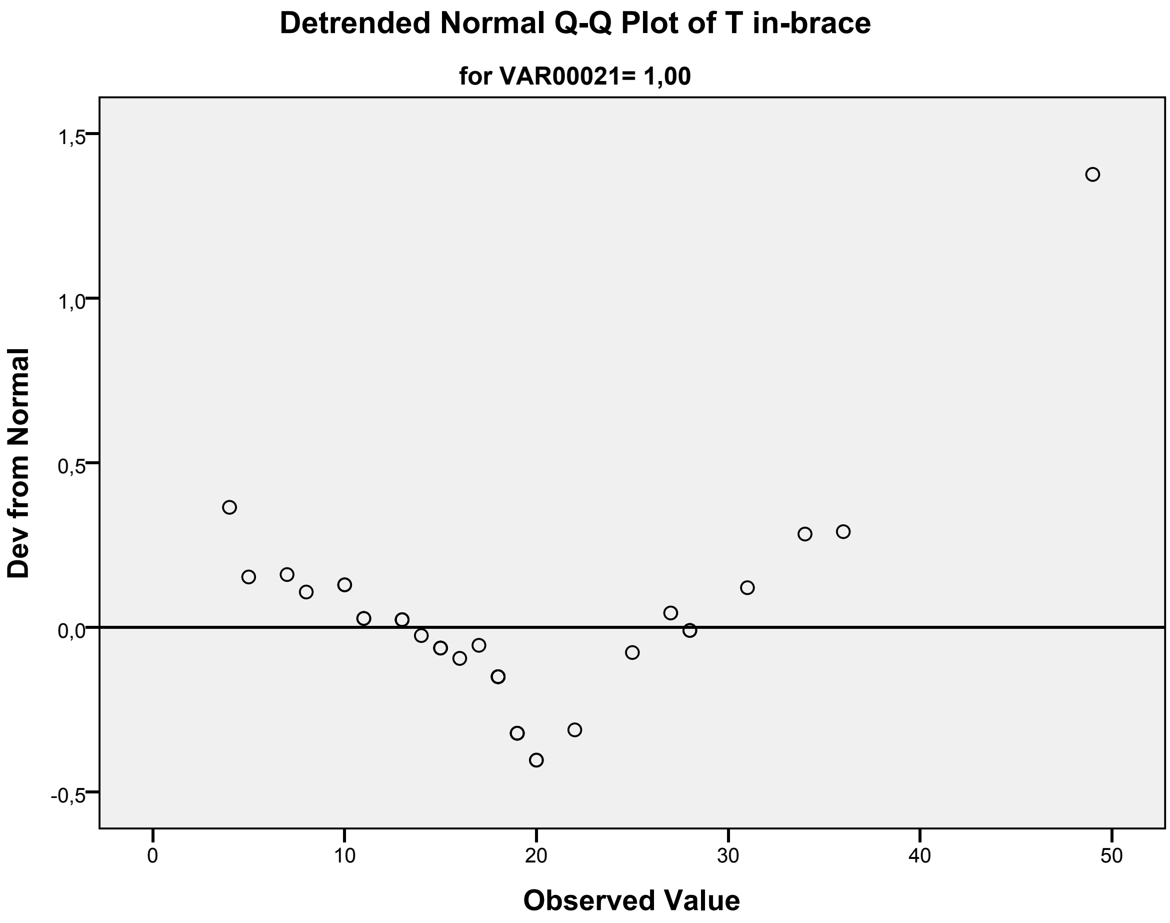
Detrended Normal Q-Q Plot of T in-brace
for VAR00021= 1,00
Dev from Normal
1,5
1,0
0,5
0,0
-0,5
0
10
20
30
40
50
Observed Value

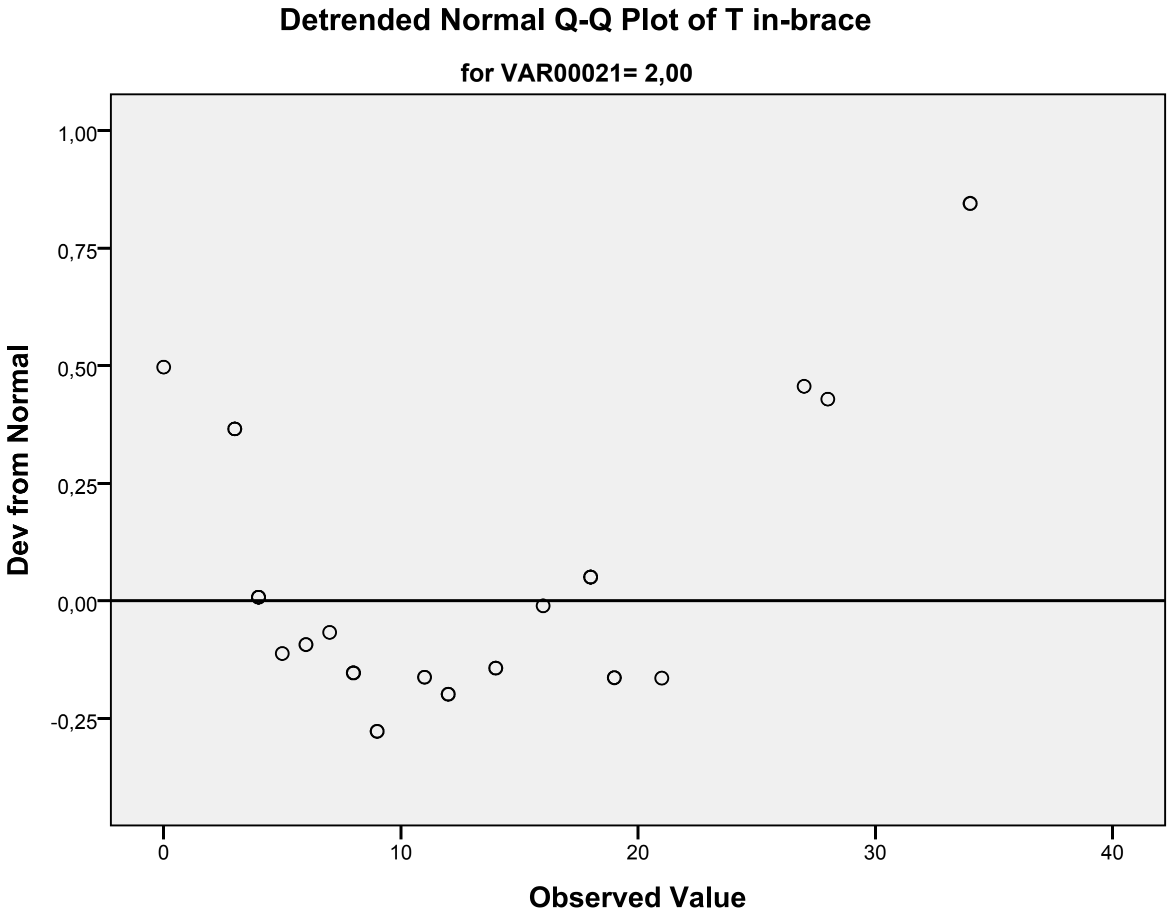
Detrended Normal Q-Q Plot of T in-brace
for VAR00021= 2,00
Dev from Normal
1,00
0,75
0,50
0,25
0,00
-0,25
0
10
20
30
40
Observed Value

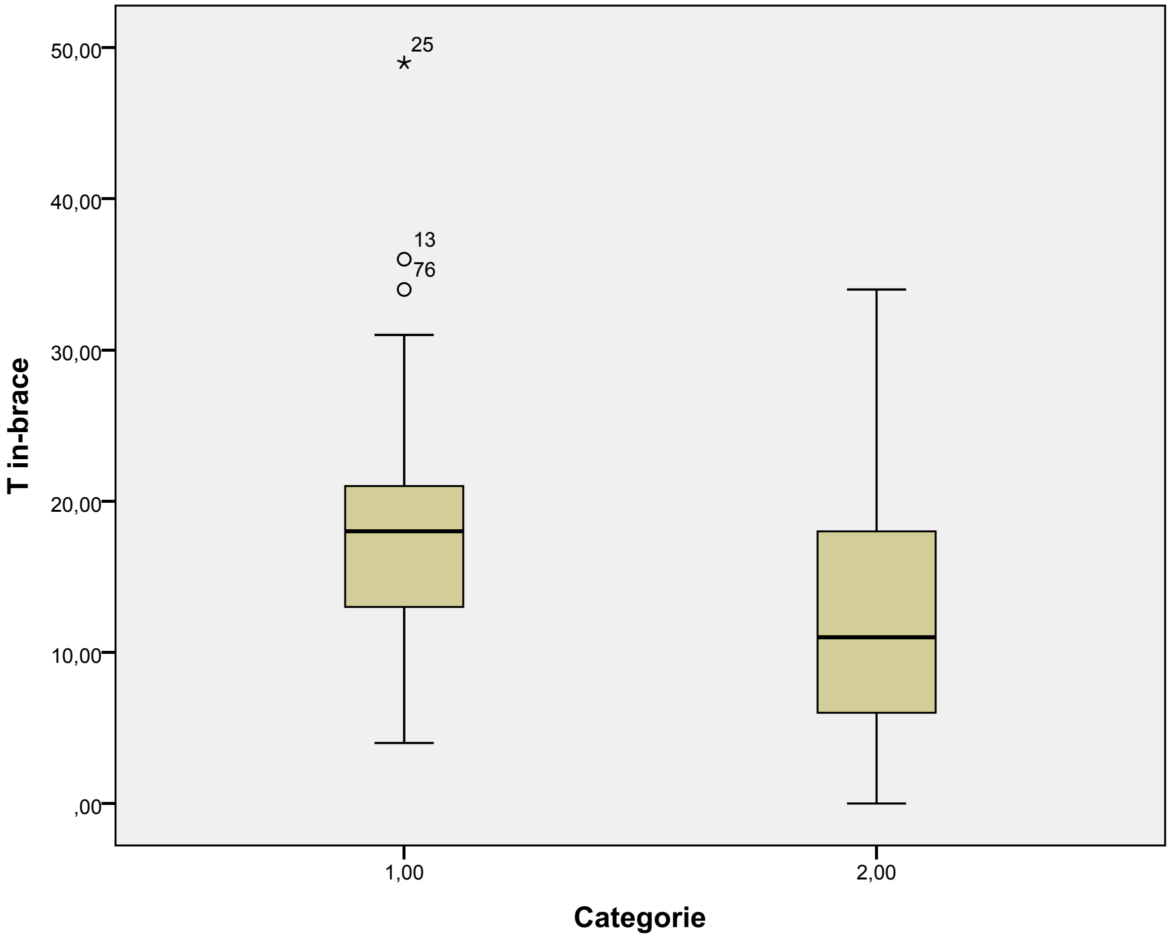

Lin-brace

Stem-and-Leaf Plots

```
Lin-brace Stem-and-Leaf Plot for
VAR00021= 1,00

 Frequency    Stem &  Leaf

     1,00        0 .  4
     9,00        0 .  556667789
    12,00        1 .  000122334444
     9,00        1 .  677889999
     2,00        2 .  04
     2,00        2 .  69
     1,00 Extremes    (>=35)

 Stem width:     10,00
 Each leaf:       1 case(s)
```

```
Lin-brace Stem-and-Leaf Plot for
VAR00021= 2,00

 Frequency    Stem &  Leaf

     5,00        0 .  00001
     7,00        0 .  2233333
     2,00        0 .  45
     5,00        0 .  66677
     5,00        0 .  88899
     4,00        1 .  0011
     1,00        1 .  3
     6,00        1 .  455555
     1,00        1 .  6
      ,00        1 .
     2,00        2 .  00
     4,00        2 .  2233
     2,00        2 .  45
     1,00        2 .  7
      ,00        2 .
     1,00        3 .  0

 Stem width:     10,00
 Each leaf:       1 case(s)
```

Normal Q-Q Plots

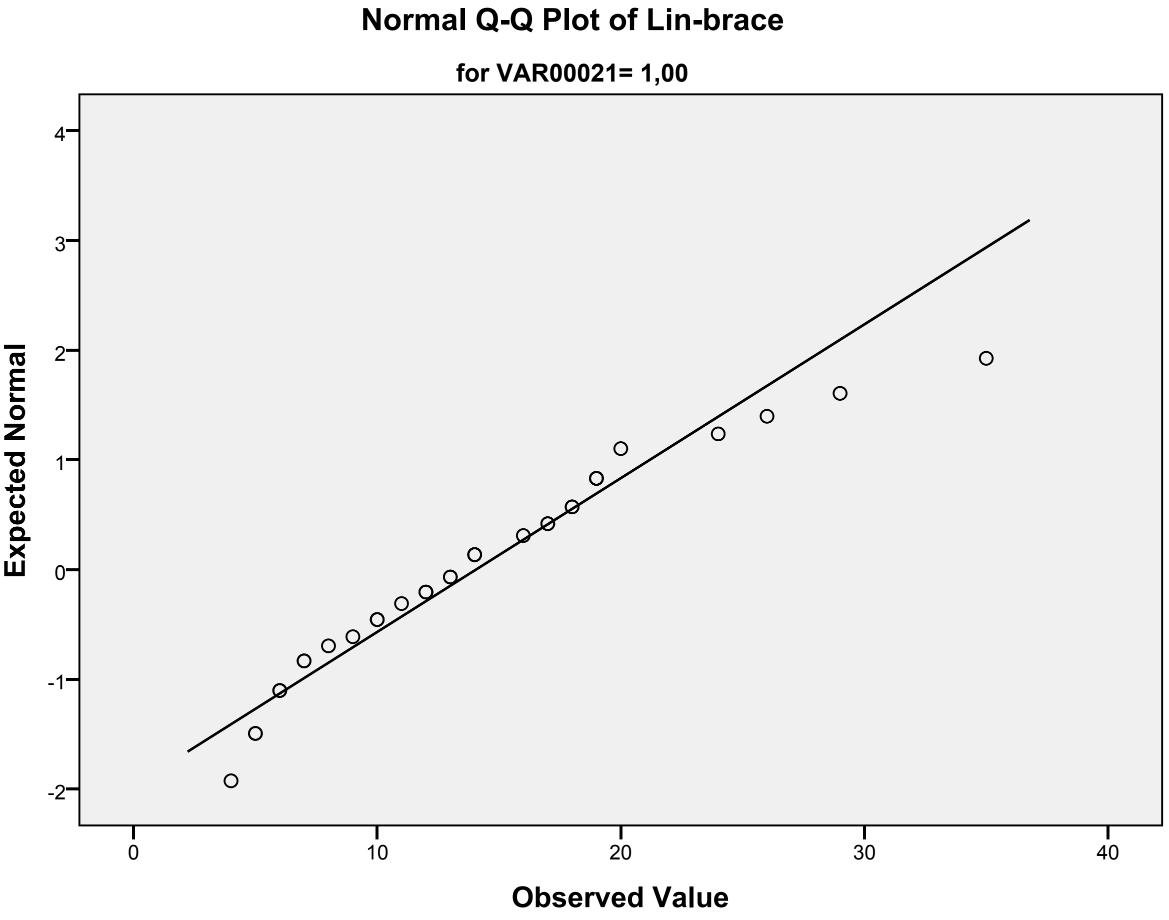
Normal Q-Q Plot of Lin-brace
for VAR00021= 1,00
Expected Normal
4
3
2
1
0
-1
-2
0
10
20
30
40
Observed Value

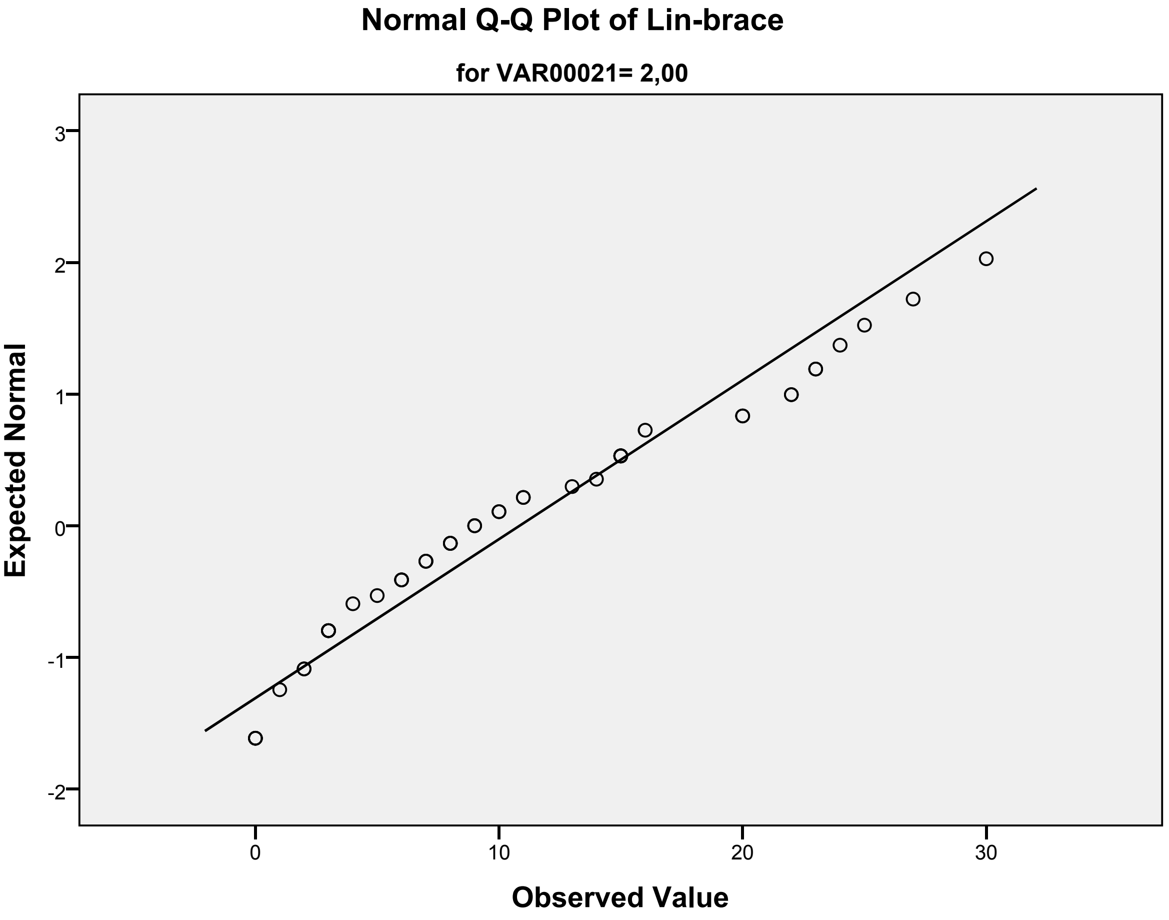

Detrended Normal Q-Q Plots

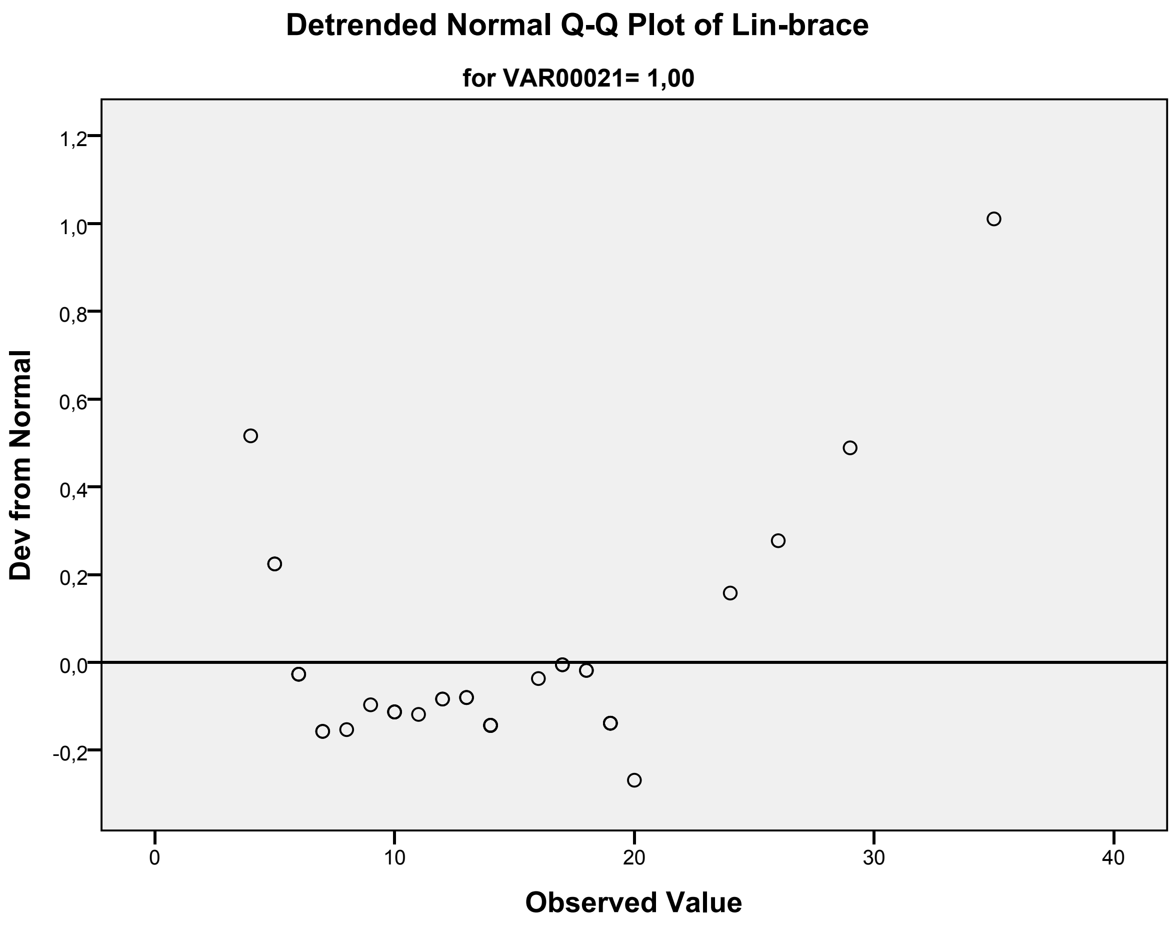
Detrended Normal Q-Q Plot of Lin-brace
for VAR00021= 1,00
Dev from Normal
1,2
1,0
0,8
0,6
0,4
0,2
0,0
-0,2
0
10
20
30
40
Observed Value

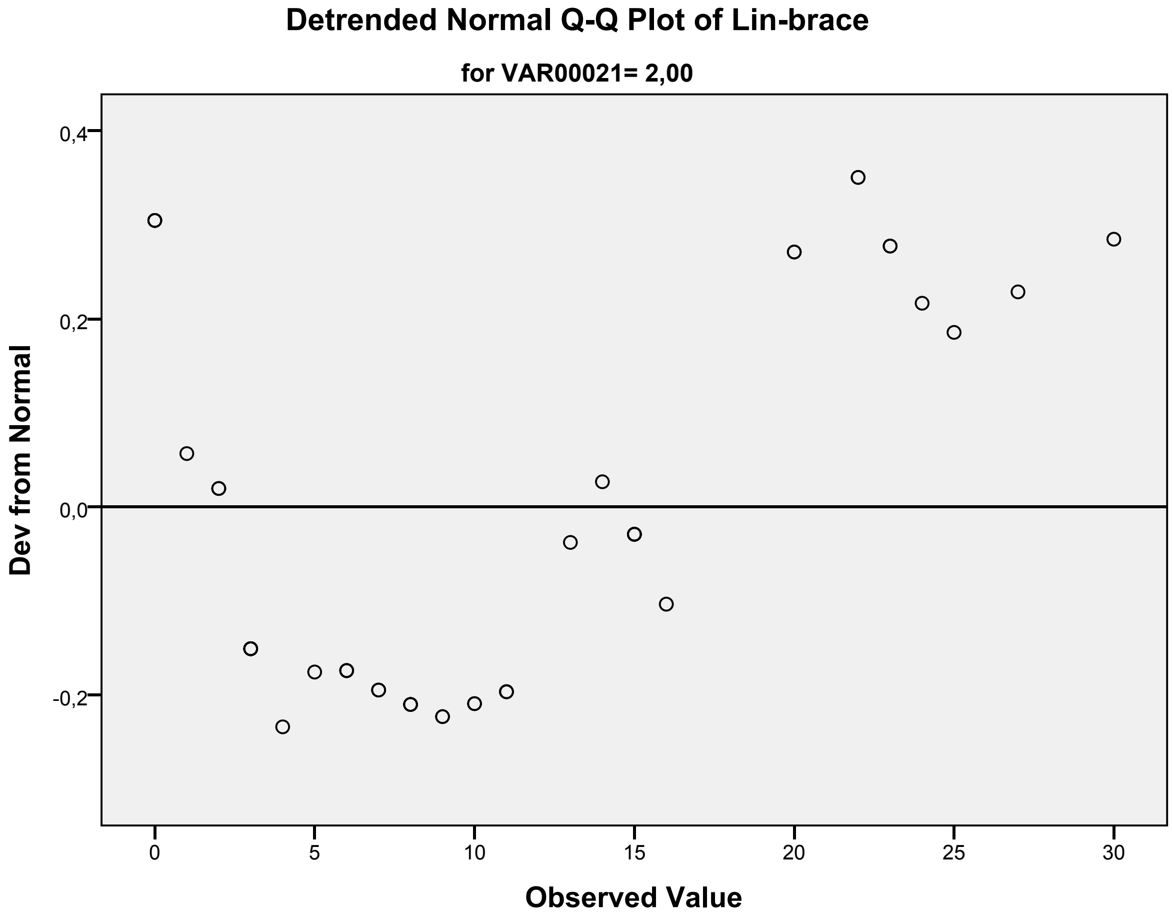
Detrended Normal Q-Q Plot of Lin-brace
for VAR00021= 2,00
Dev from Normal
0,4
0,2
0,0
-0,2
0
5
10
15
20
25
30
Observed Value

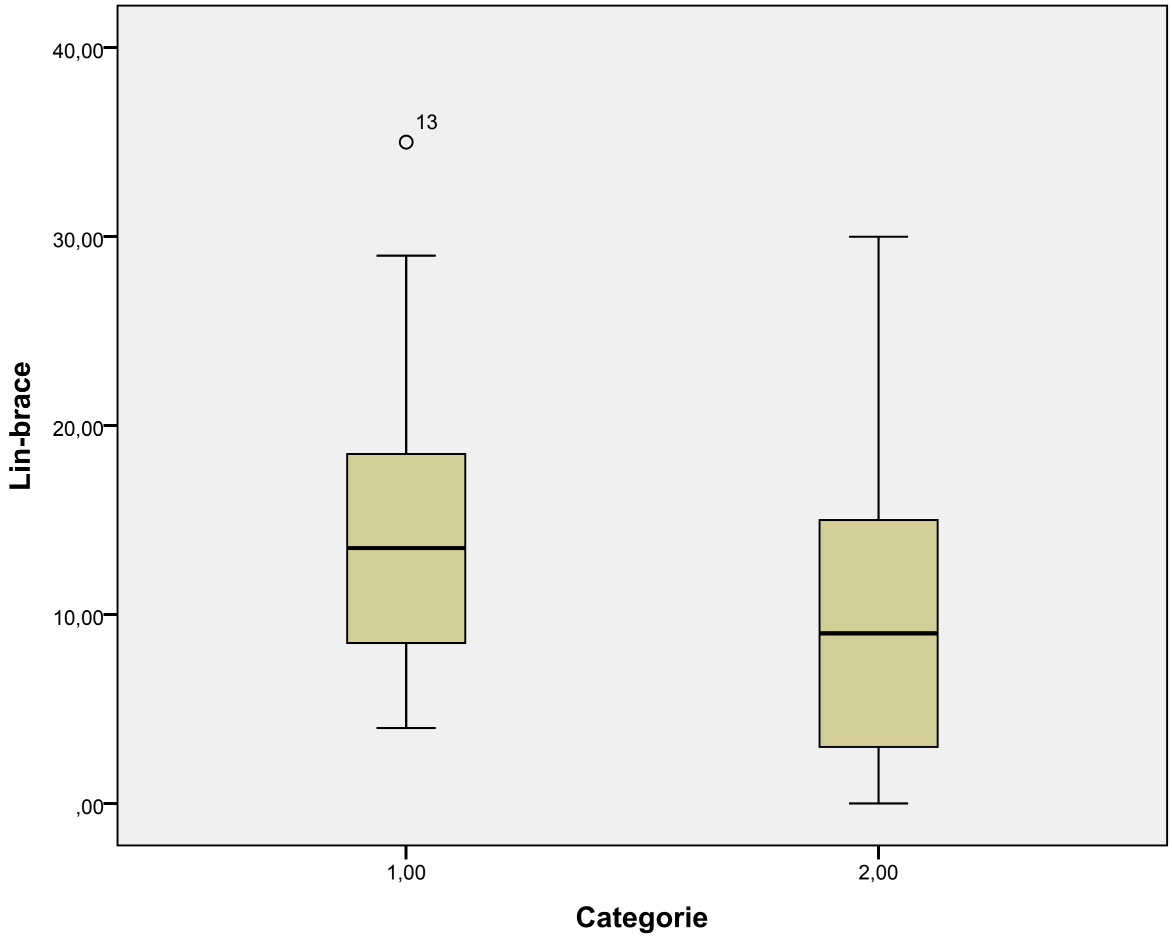

T 6 months

Stem-and-Leaf Plots

```
T 6 months Stem-and-Leaf Plot for
VAR00021= 1,00

 Frequency    Stem &  Leaf

     4,00        1 .  0024
    10,00        1 .  5567899999
     5,00        2 .  12444
     8,00        2 .  55578889
     3,00        3 .  013
     3,00        3 .  678
     3,00 Extremes    (>=52)

 Stem width:     10,00
 Each leaf:       1 case(s)
```

```
T 6 months Stem-and-Leaf Plot for
VAR00021= 2,00

 Frequency    Stem &  Leaf

     1,00        0 .  2
     4,00        0 .  7899
     7,00        1 .  0022234
    12,00        1 .  666777888999
     8,00        2 .  00001133
     7,00        2 .  6688899
     1,00        3 .  2
     2,00        3 .  99
     2,00        4 .  11
     1,00        4 .  5
     1,00 Extremes    (>=52)

 Stem width:     10,00
 Each leaf:       1 case(s)
```

Normal Q-Q Plots

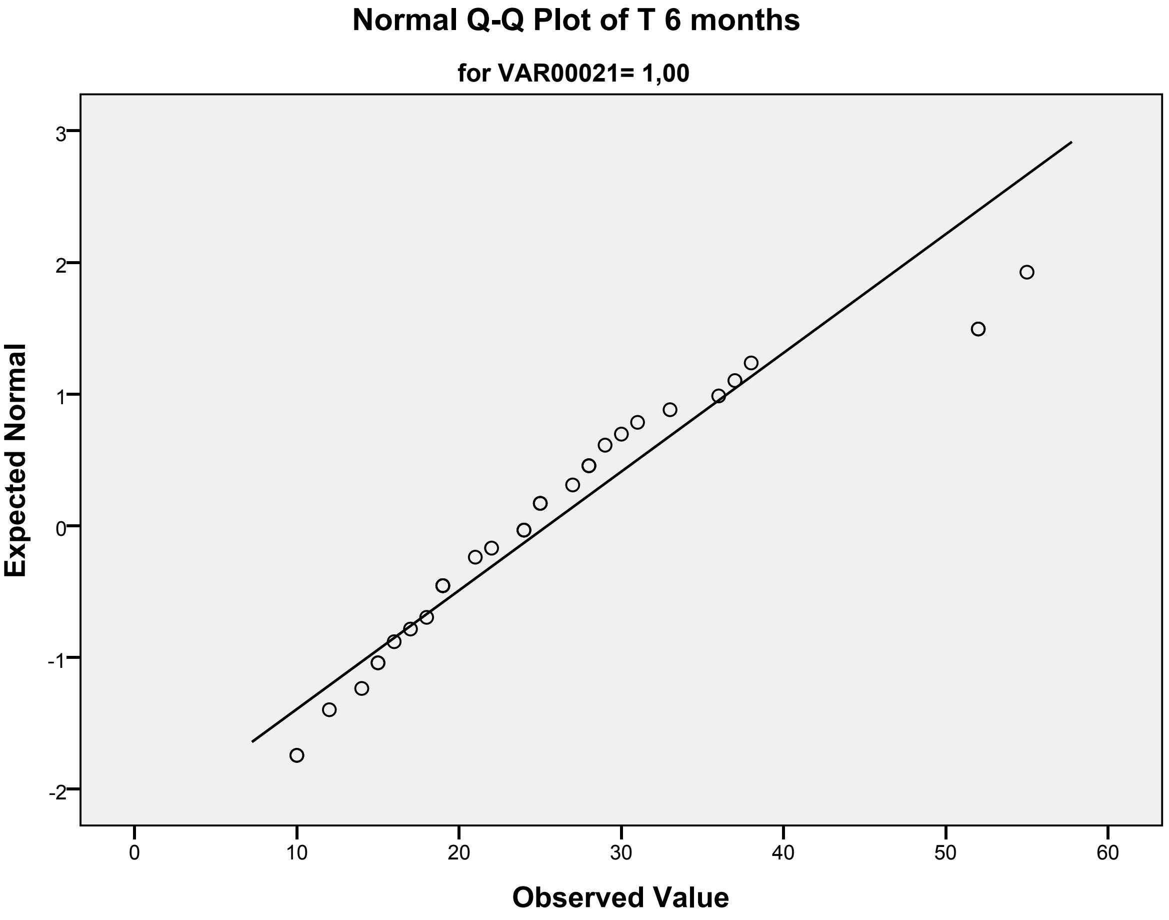
Normal Q-Q Plot of T 6 months
for VAR00021= 1,00
Expected Normal
3
2
1
0
-1
-2
0
10
20
30
40
50
60
Observed Value

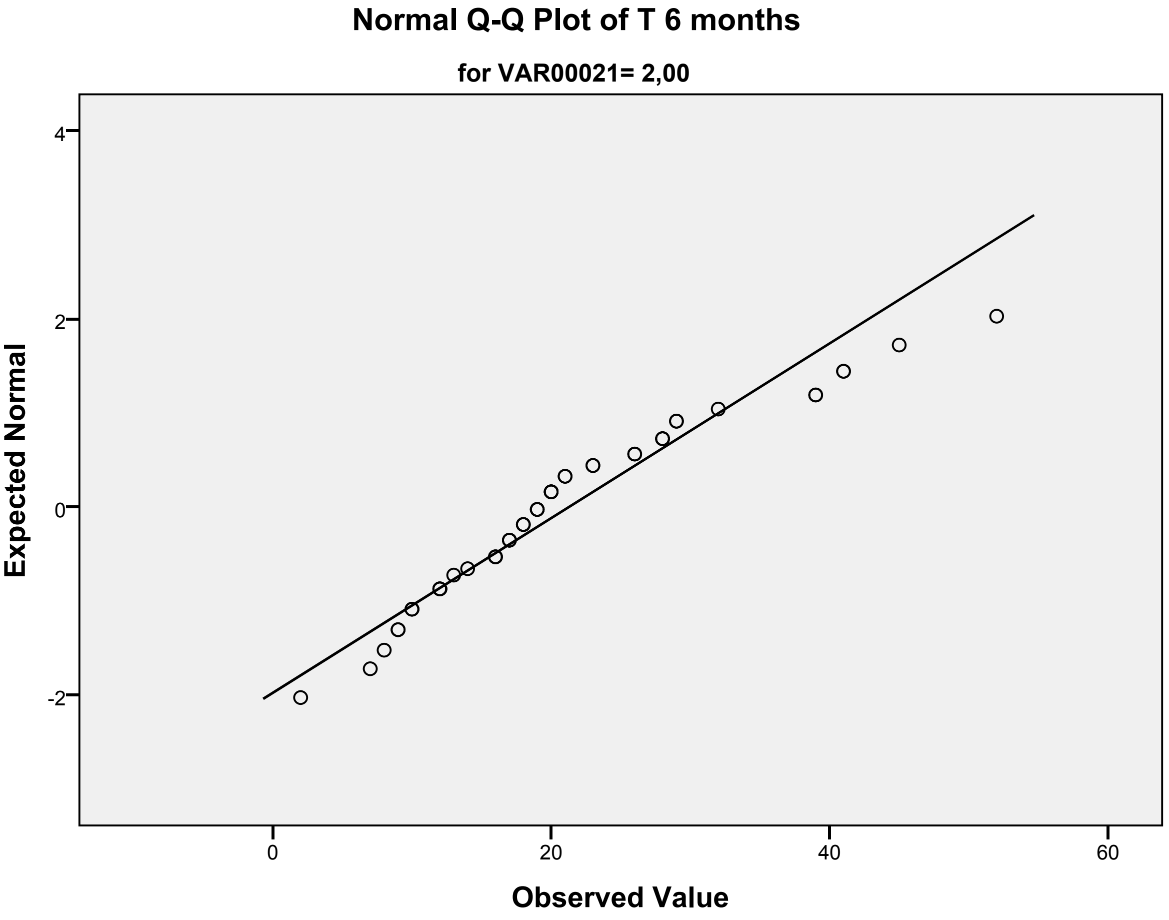

Detrended Normal Q-Q Plots

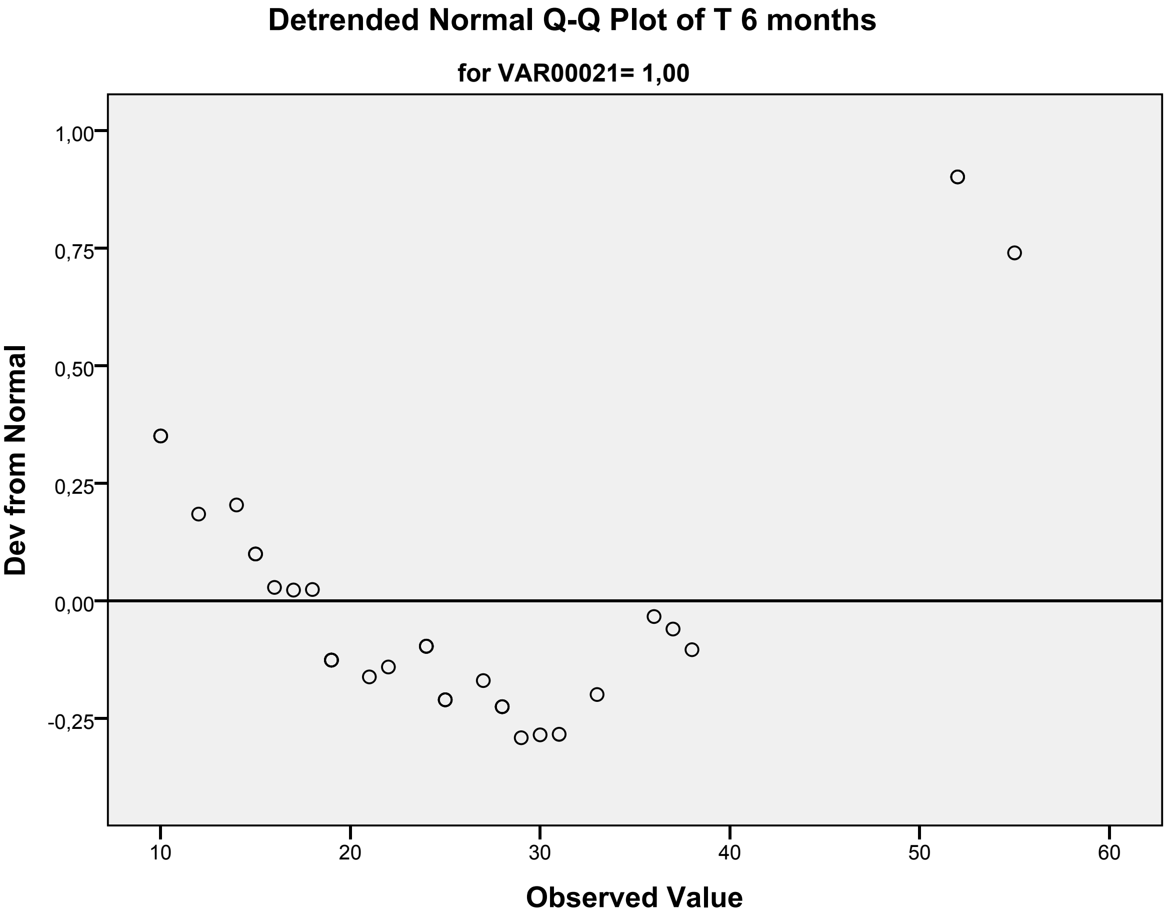

Detrended Normal Q-Q Plot of T 6 months
for VAR00021= 1,00
Dev from Normal
1,00
0,75
0,50
0,25
0,00
-0,25
10
20
30
40
50
60
Observed Value

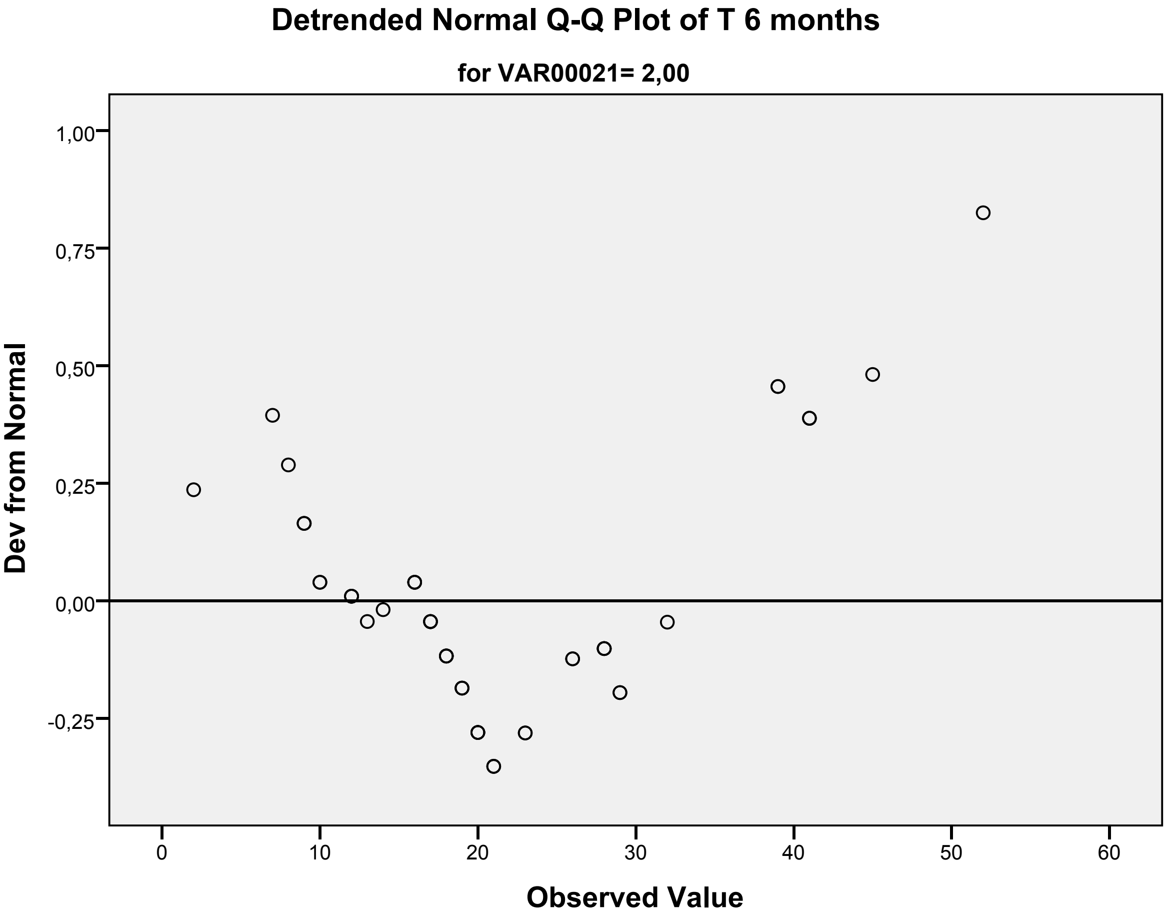

Detrended Normal Q-Q Plot of T 6 months
for VAR00021= 2,00
Dev from Normal
1,00
0,75
0,50
0,25
0,00
-0,25
0
10
20
30
40
50
60
Observed Value

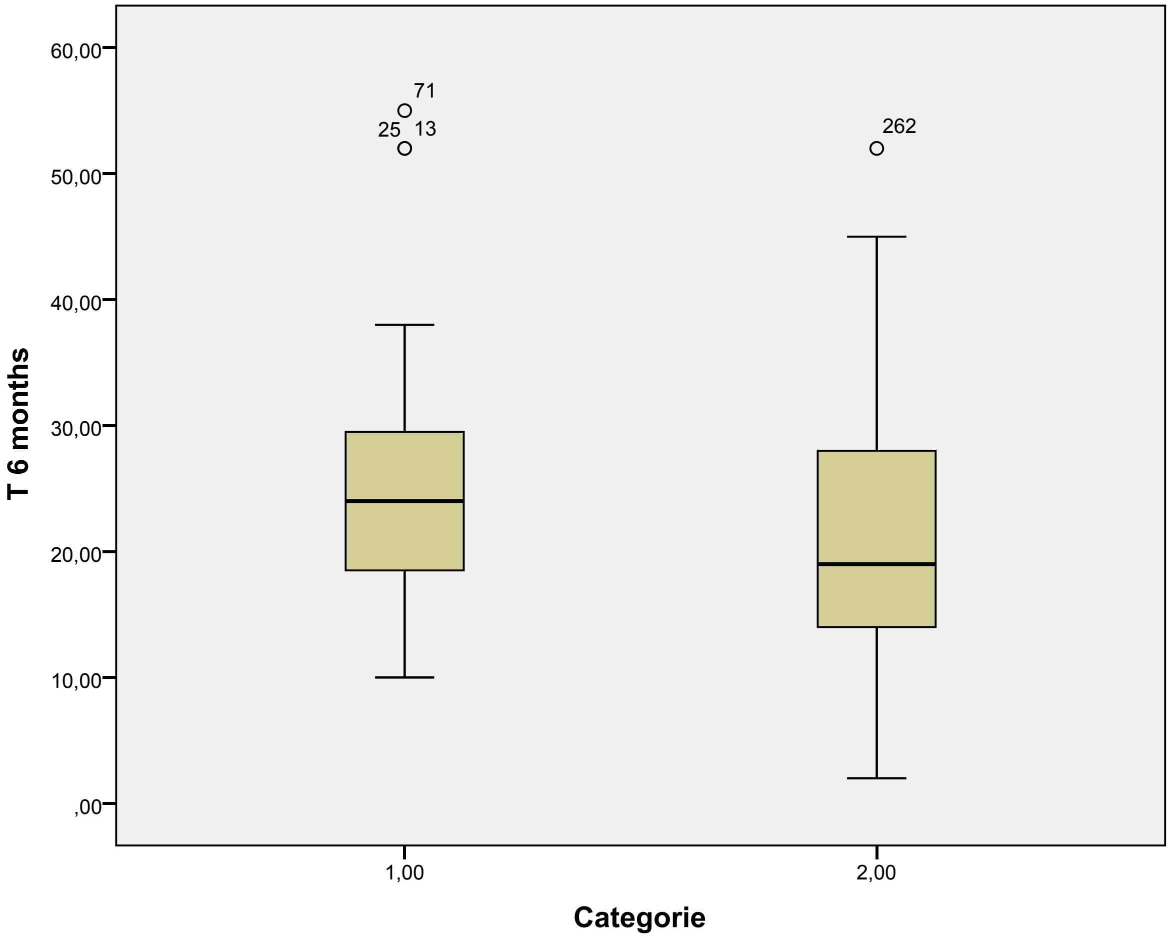

L 6 months

Stem-and-Leaf Plots

```
L 6 months Stem-and-Leaf Plot for
VAR00021= 1,00

 Frequency    Stem &  Leaf

     2,00        0 .  99
     6,00        1 .  000234
    11,00        1 .  55567778899
     8,00        2 .  00112233
     5,00        2 .  56679
     2,00        3 .  23
     2,00 Extremes    (>=40)

 Stem width:     10,00
 Each leaf:       1 case(s)
```

```
L 6 months Stem-and-Leaf Plot for
VAR00021= 2,00

 Frequency    Stem &  Leaf

     1,00        0 .  3
     4,00        0 .  6999
    10,00        1 .  0012223333
    13,00        1 .  5566777888889
     4,00        2 .  0001
     6,00        2 .  555689
     5,00        3 .  02234
     2,00        3 .  69
     1,00        4 .  0

 Stem width:     10,00
 Each leaf:       1 case(s)
```

Normal Q-Q Plots

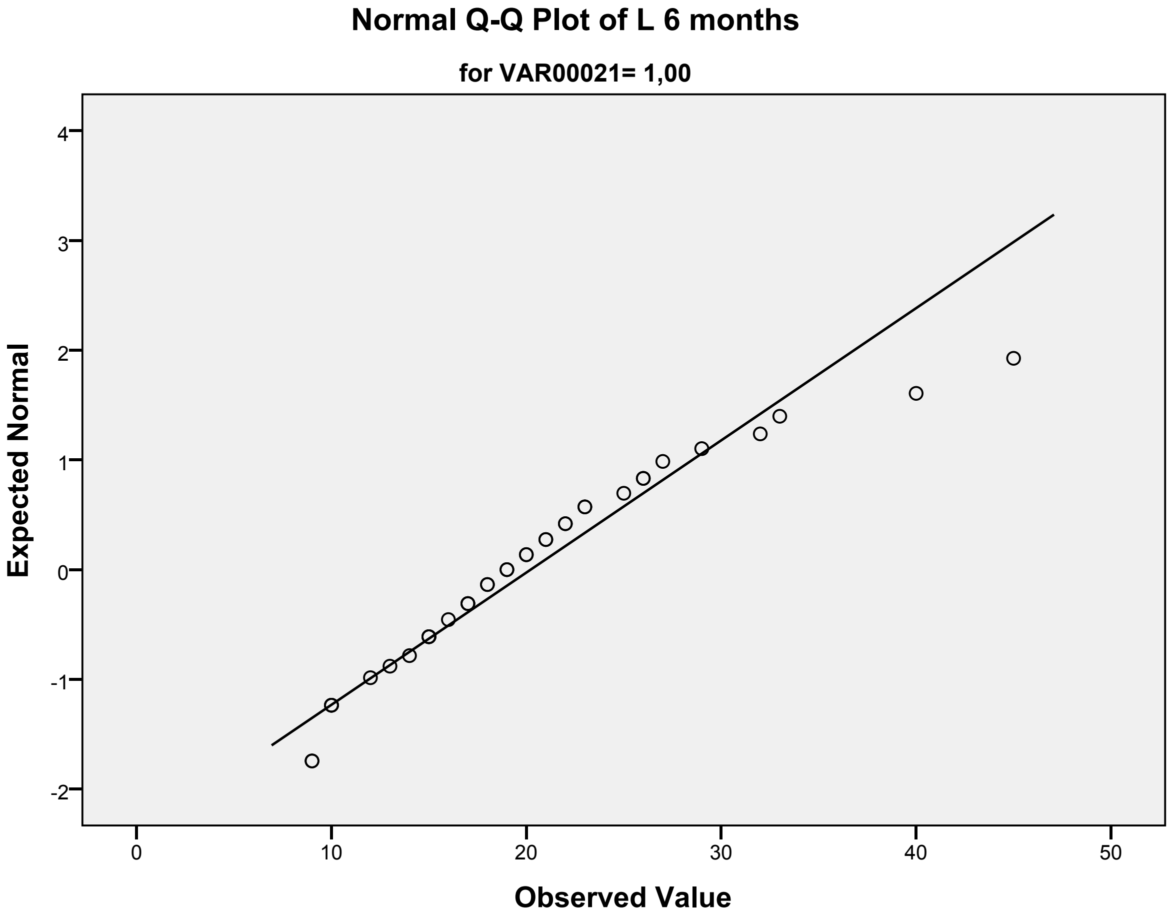
Normal Q-Q Plot of L 6 months
for VAR00021= 1,00
Expected Normal
4
3
2
1
0
-1
-2
0
10
20
30
40
50
Observed Value

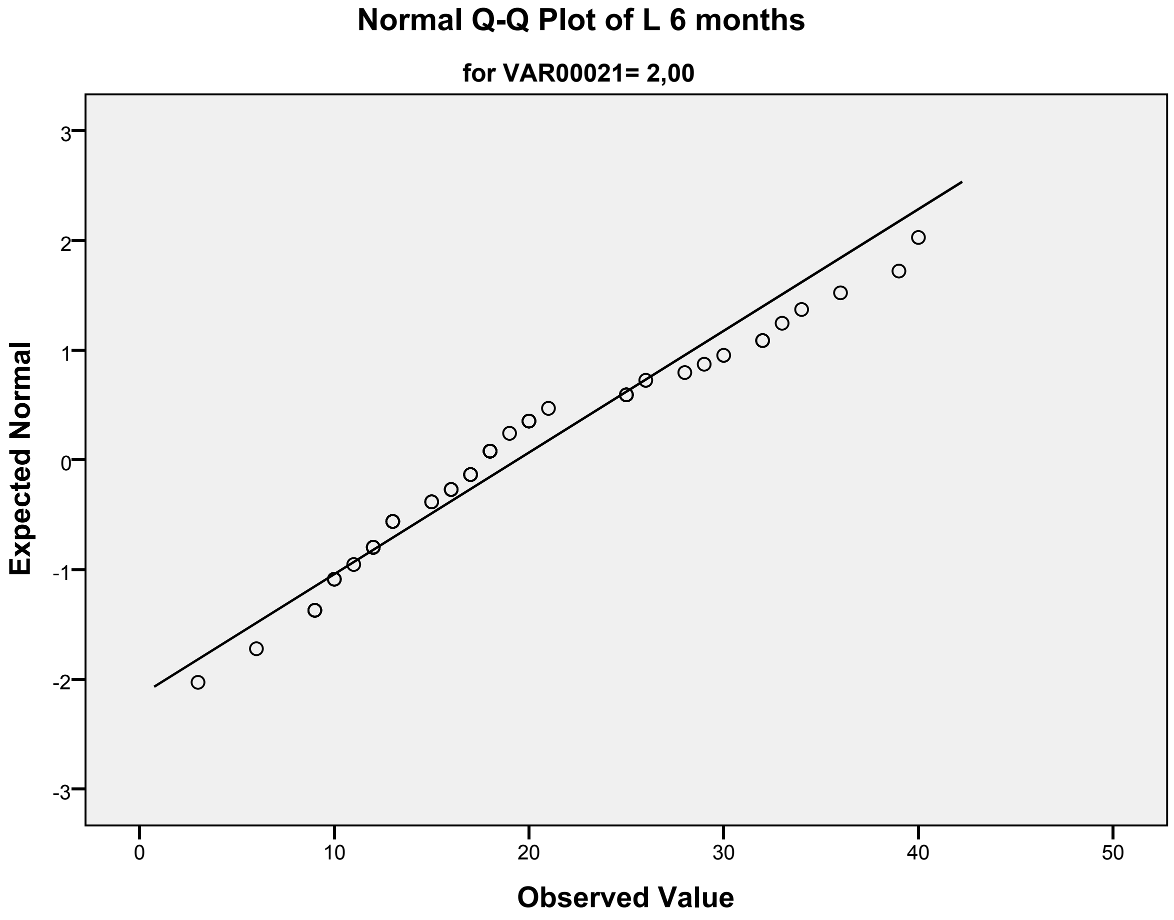

Detrended Normal Q-Q Plots

Detrended Normal Q-Q Plot of L 6 months

for VAR00021= 1,00

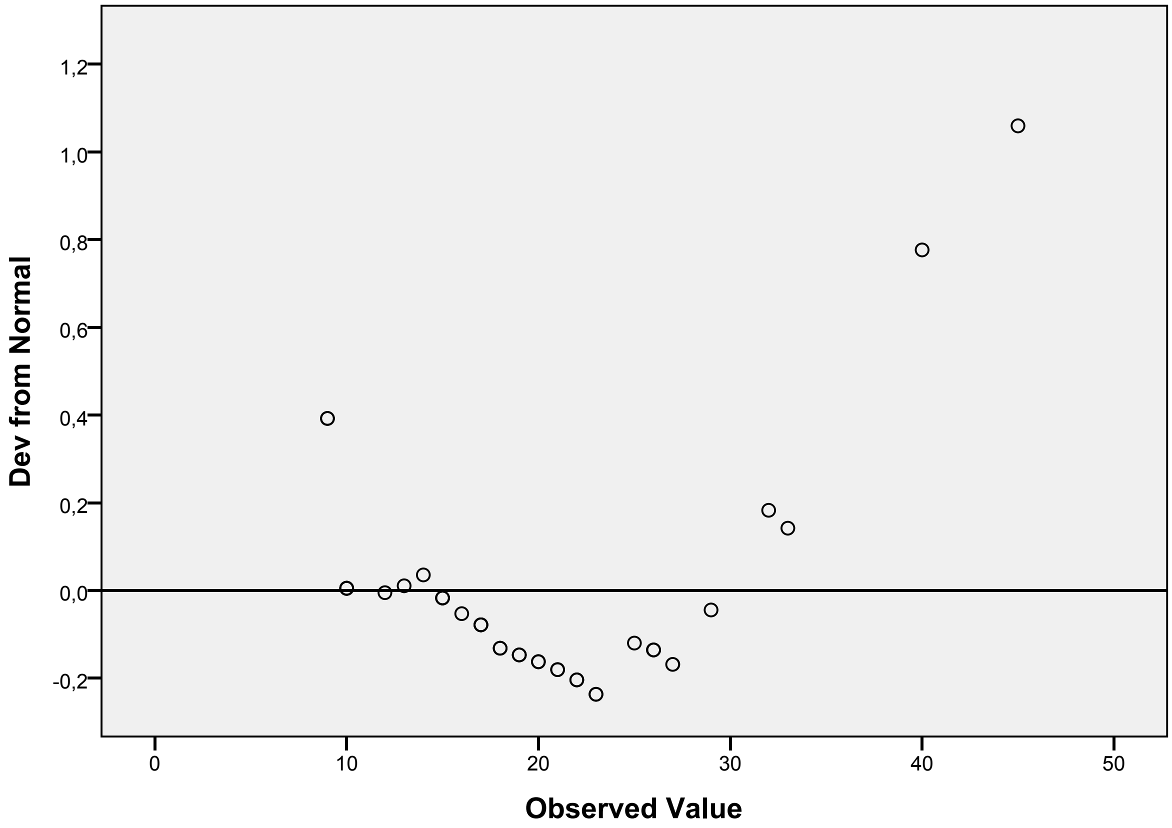

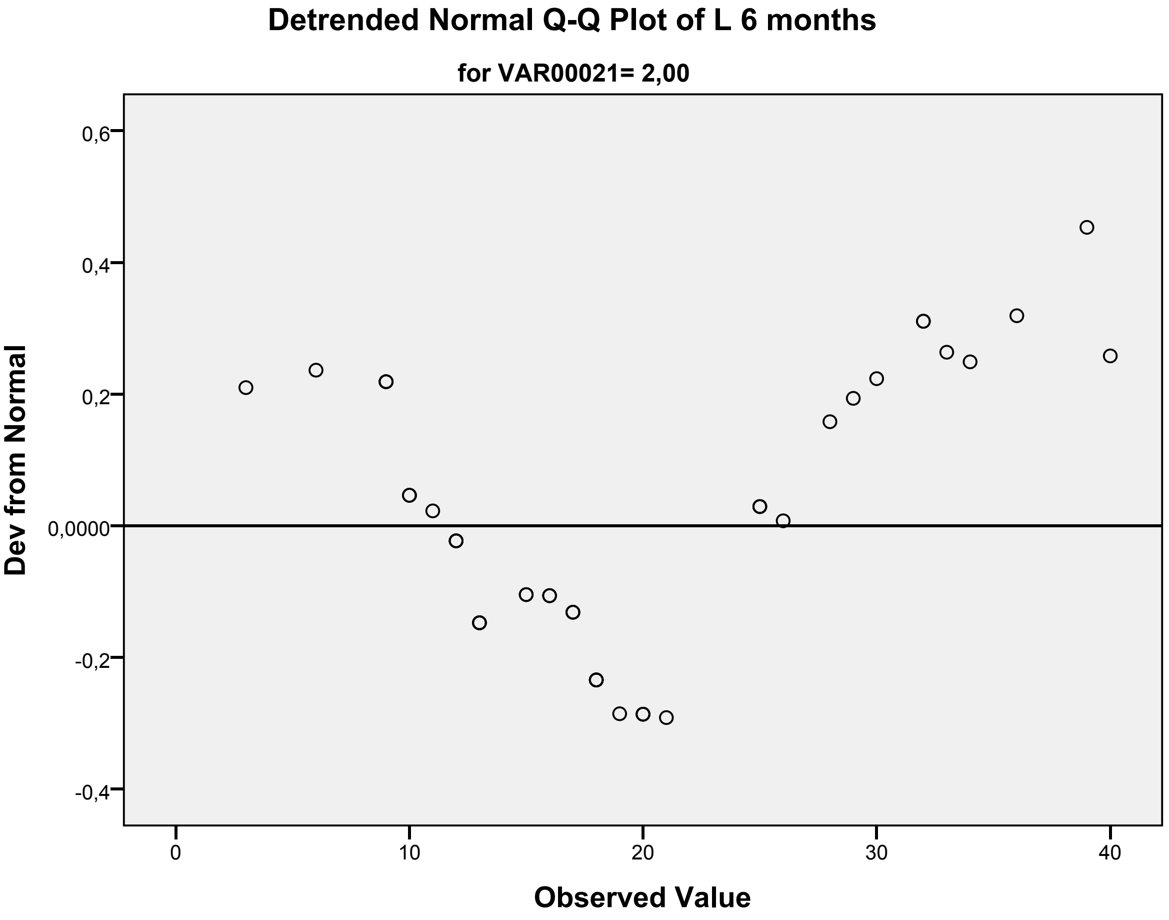
Detrended Normal Q-Q Plot of L 6 months
for VAR00021= 2,00
Dev from Normal
0,6
0,4
0,2
0,0000
-0,2
-0,4
0
10
20
30
40
Observed Value

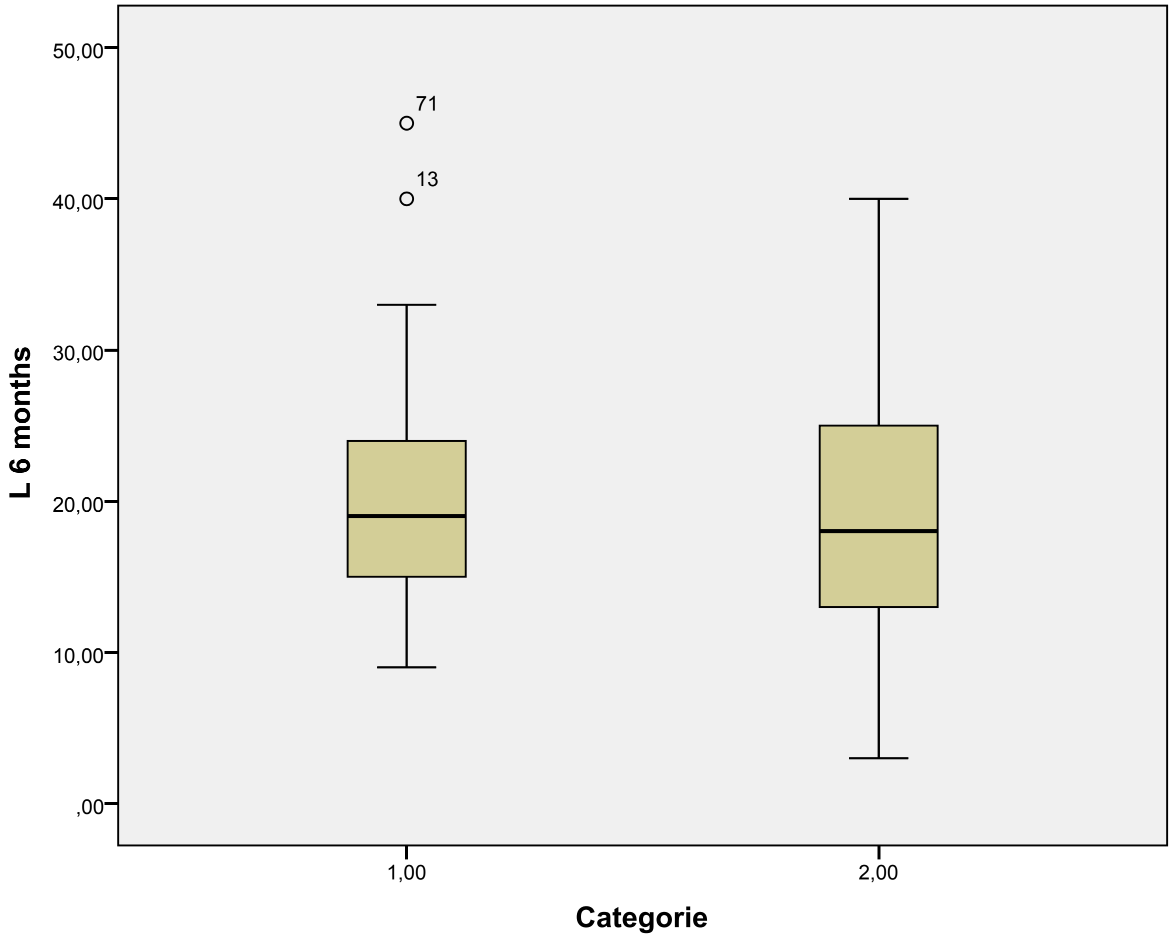

T 1 year

Stem-and-Leaf Plots

```
T 1 year Stem-and-Leaf Plot for
VAR00021= 1,00

 Frequency    Stem &  Leaf

     1,00        1 .  4
     8,00        1 .  56677889
     7,00        2 .  0002234
     7,00        2 .  5777899
     5,00        3 .  00113
     1,00        3 .  6
     3,00        4 .  013
     1,00        4 .  7
     3,00 Extremes    (>=52)

 Stem width:     10,00
 Each leaf:       1 case(s)
```

```
T 1 year Stem-and-Leaf Plot for
VAR00021= 2,00

 Frequency    Stem &  Leaf

      ,00        0 .
     4,00        0 .  5777
     5,00        1 .  01234
    11,00        1 .  55678899999
    10,00        2 .  0000012223
     5,00        2 .  58889
     6,00        3 .  111344
     3,00        3 .  799
     2,00        4 .  13

 Stem width:     10,00
 Each leaf:       1 case(s)
```

Normal Q-Q Plots

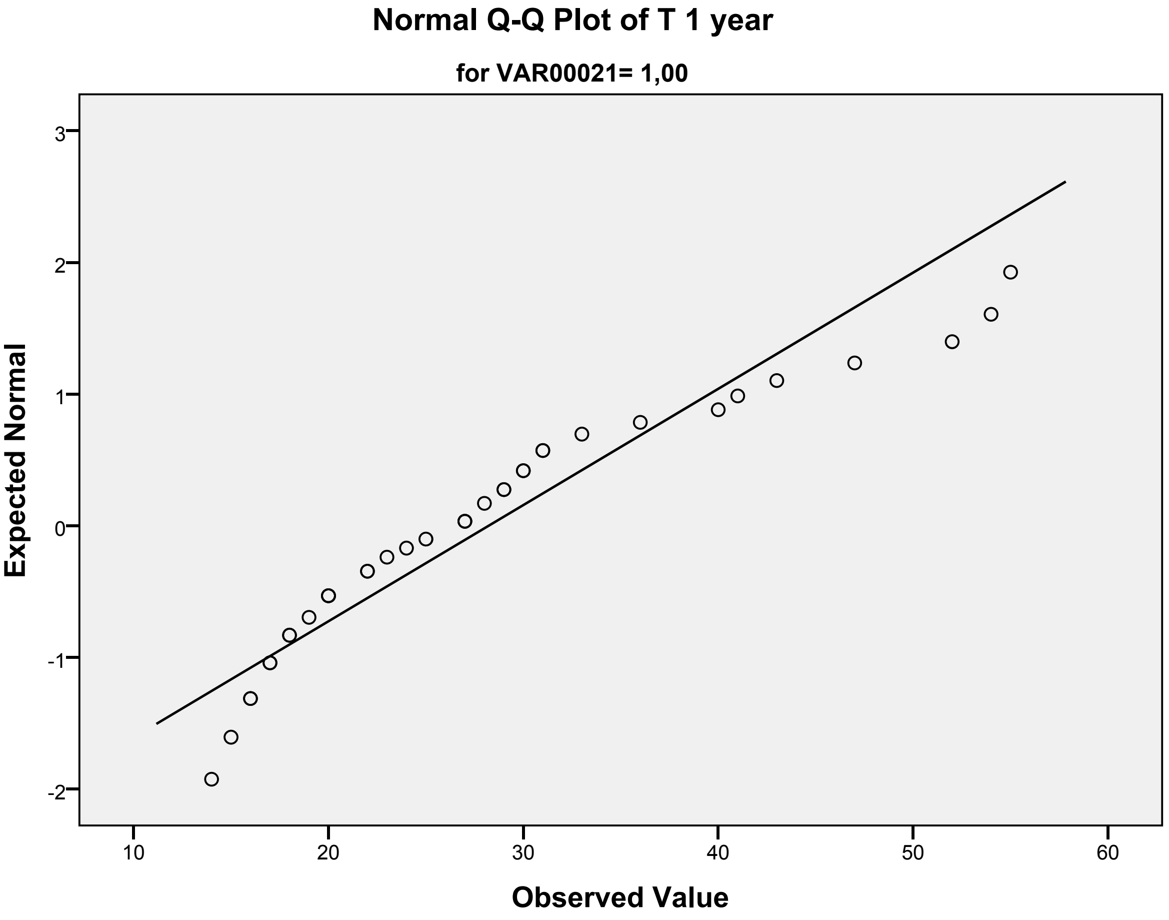
Normal Q-Q Plot of T 1 year
for VAR00021= 1,00
Expected Normal
Observed Value
3
2
1
0
-1
-2
10
20
30
40
50
60

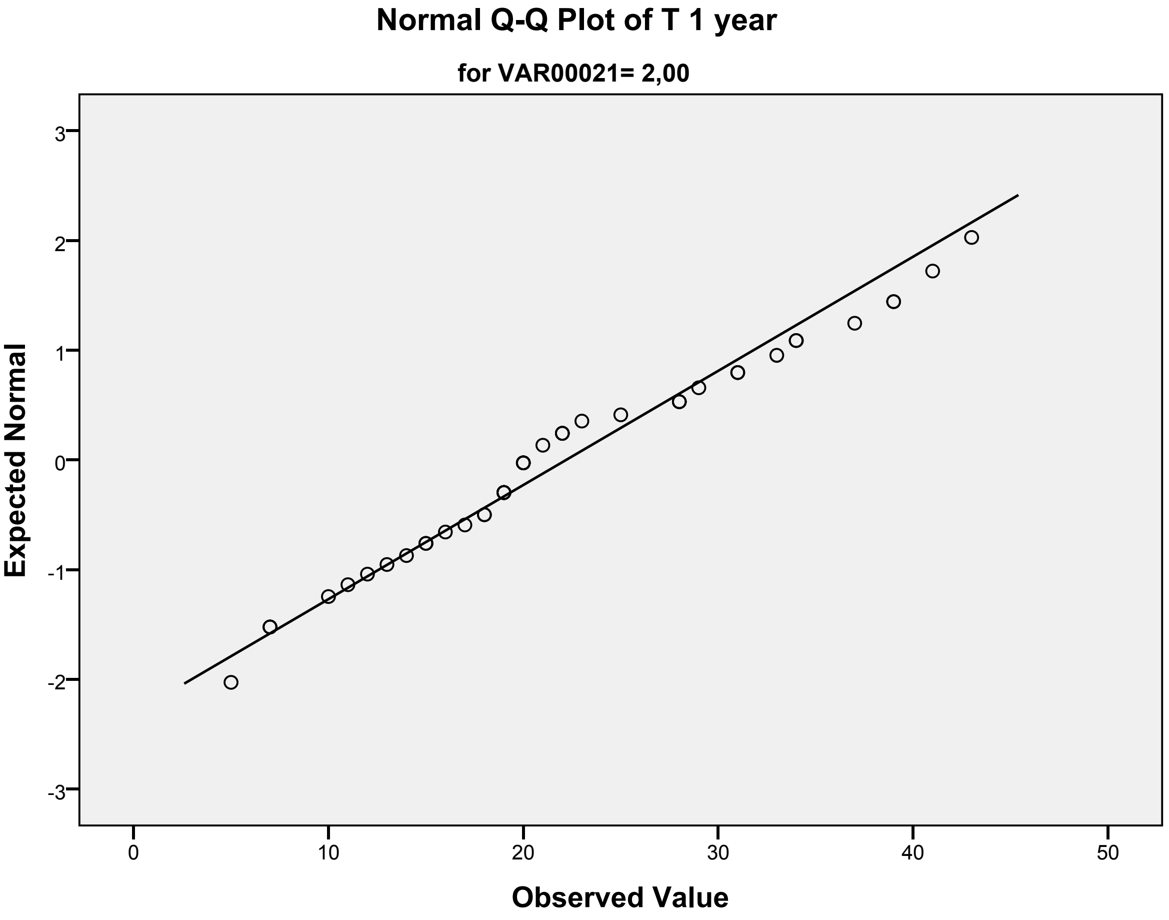

Detrended Normal Q-Q Plots

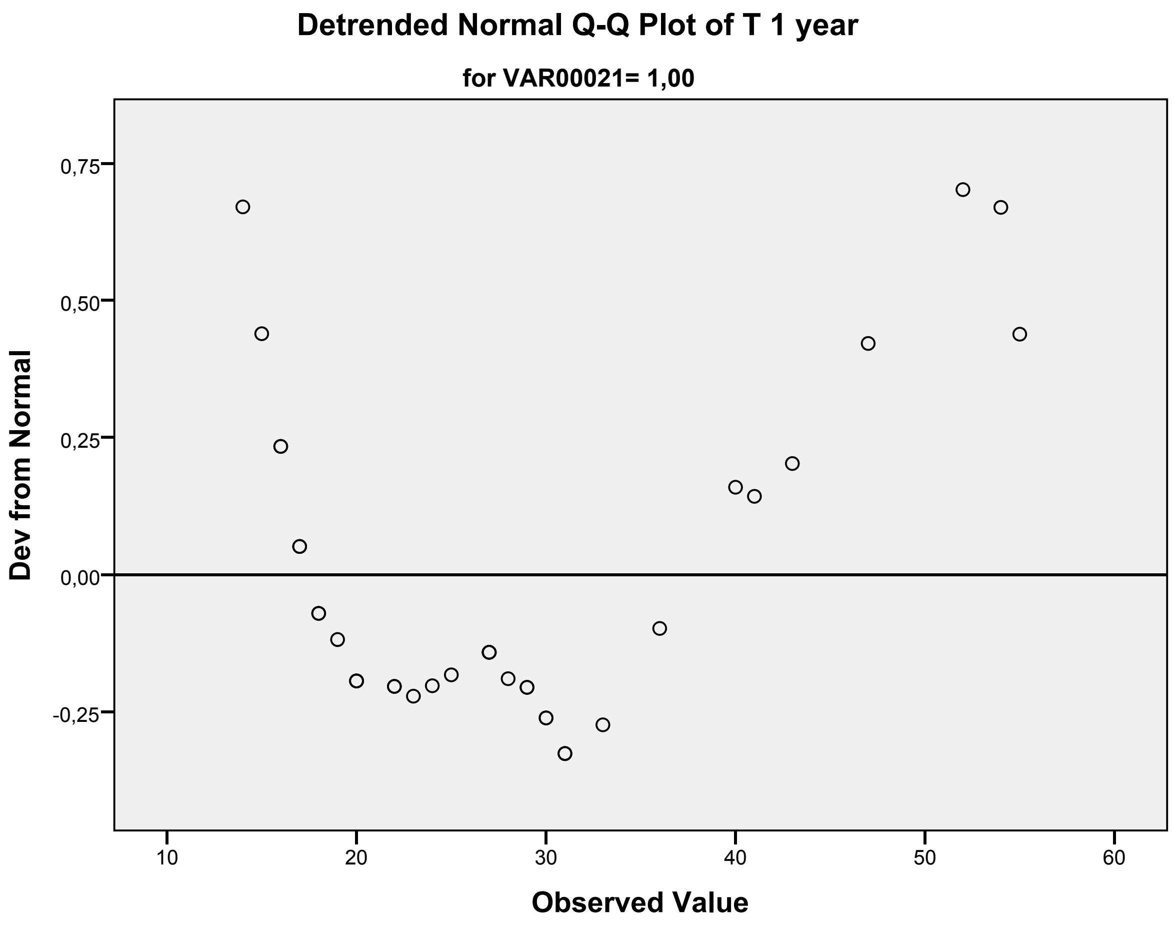
Detrended Normal Q-Q Plot of T 1 year
for VAR00021= 1,00
Dev from Normal
0,75
0,50
0,25
0,00
-0,25
10
20
30
40
50
60
Observed Value

Detrended Normal Q-Q Plot of T 1 year

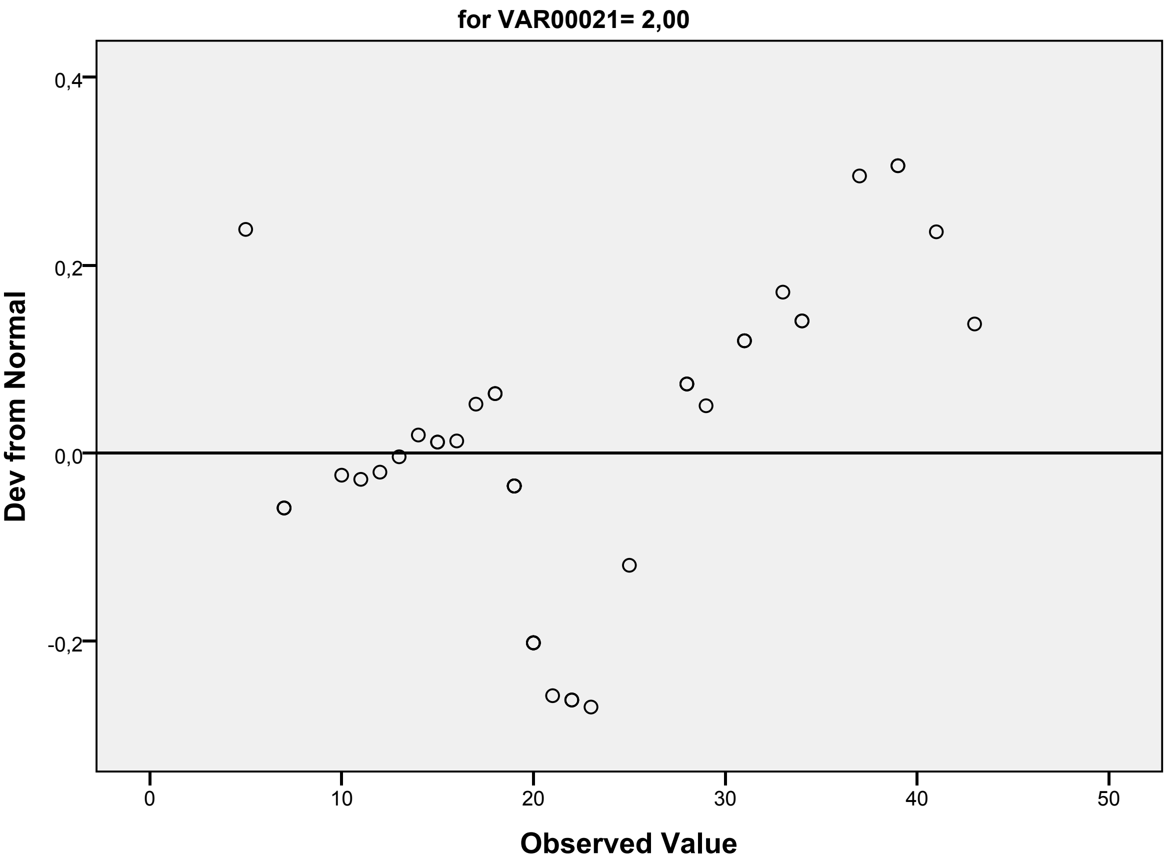

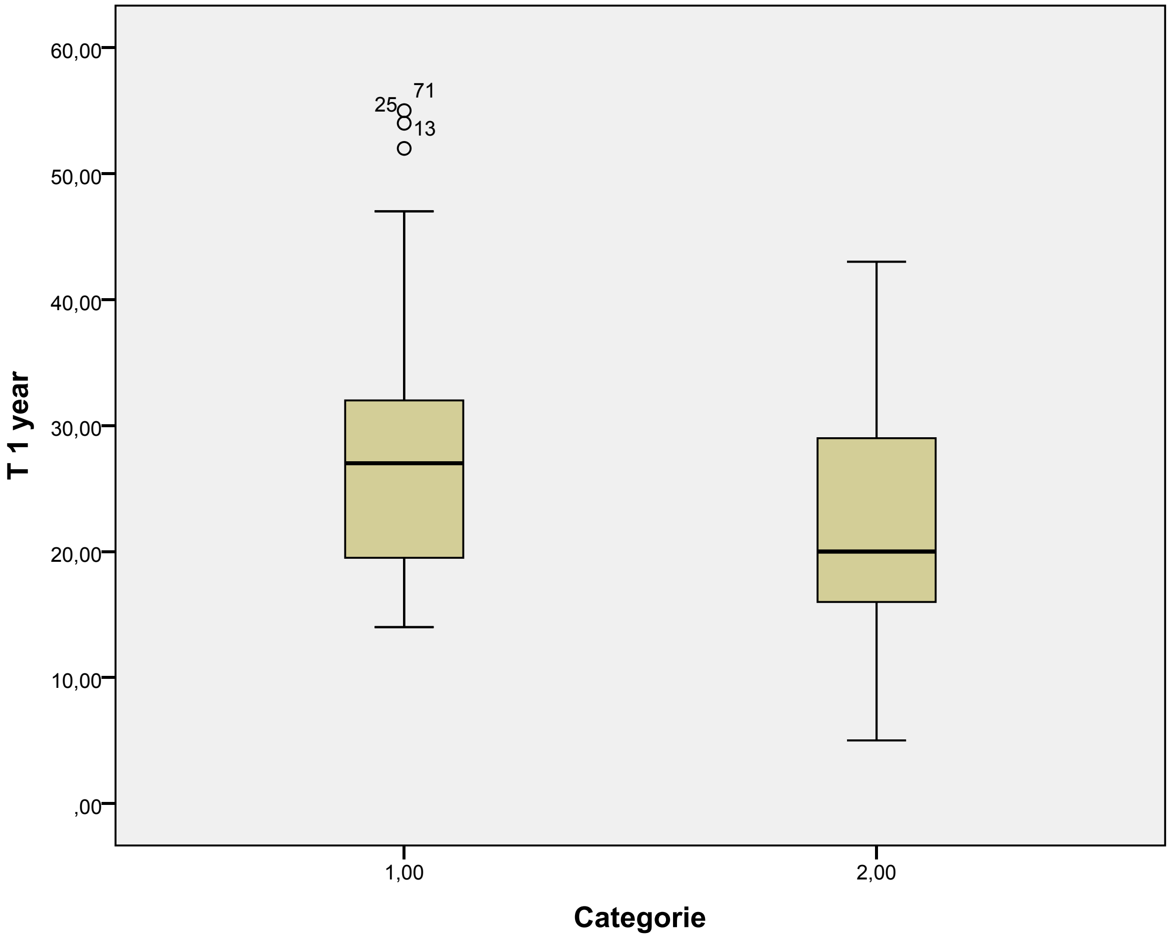

L 1 year

Stem-and-Leaf Plots

```
L 1 year Stem-and-Leaf Plot for
VAR00021= 1,00

 Frequency    Stem &  Leaf

     6,00        1 .  023444
     8,00        1 .  55566779
    12,00        2 .  000011223444
     5,00        2 .  56888
     3,00        3 .  003
     2,00 Extremes    (>=45)

 Stem width:     10,00
 Each leaf:       1 case(s)
```

```
L 1 year Stem-and-Leaf Plot for
VAR00021= 2,00

 Frequency    Stem &  Leaf

     1,00        0 .  2
     2,00        0 .  68
     7,00        1 .  0000112
    16,00        1 .  5555566777788889
     6,00        2 .  000123
     5,00        2 .  56899
     5,00        3 .  11234
     2,00        3 .  55
     2,00        4 .  00

 Stem width:     10,00
 Each leaf:       1 case(s)
```

Normal Q-Q Plots

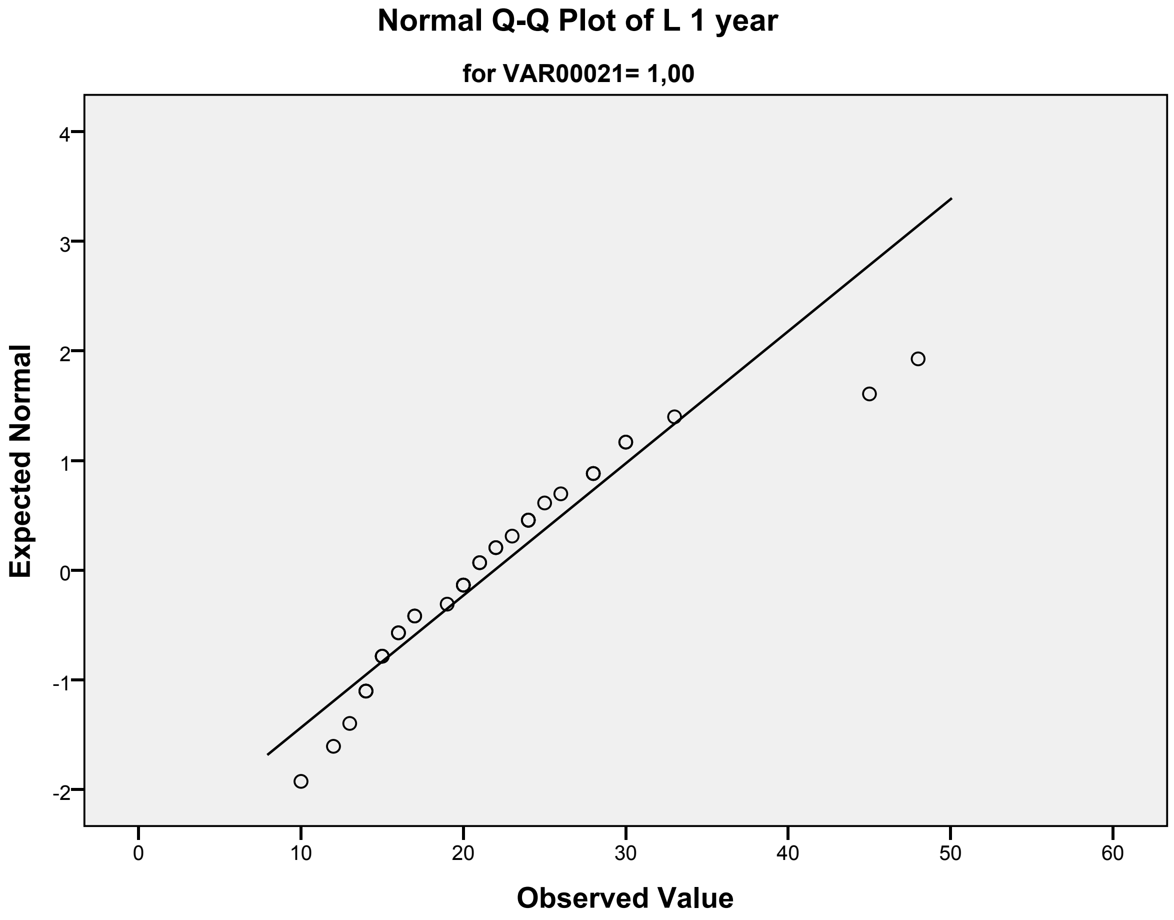

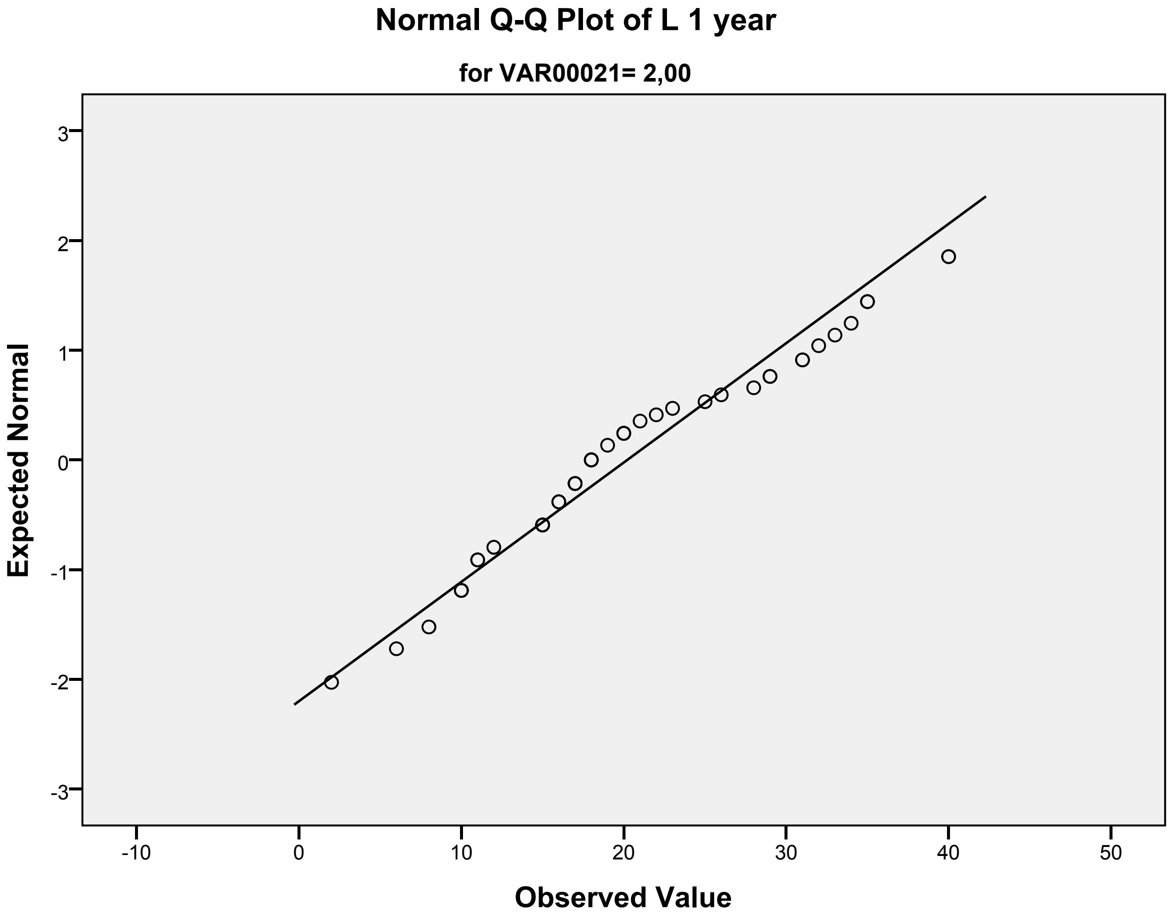

Detrended Normal Q-Q Plots

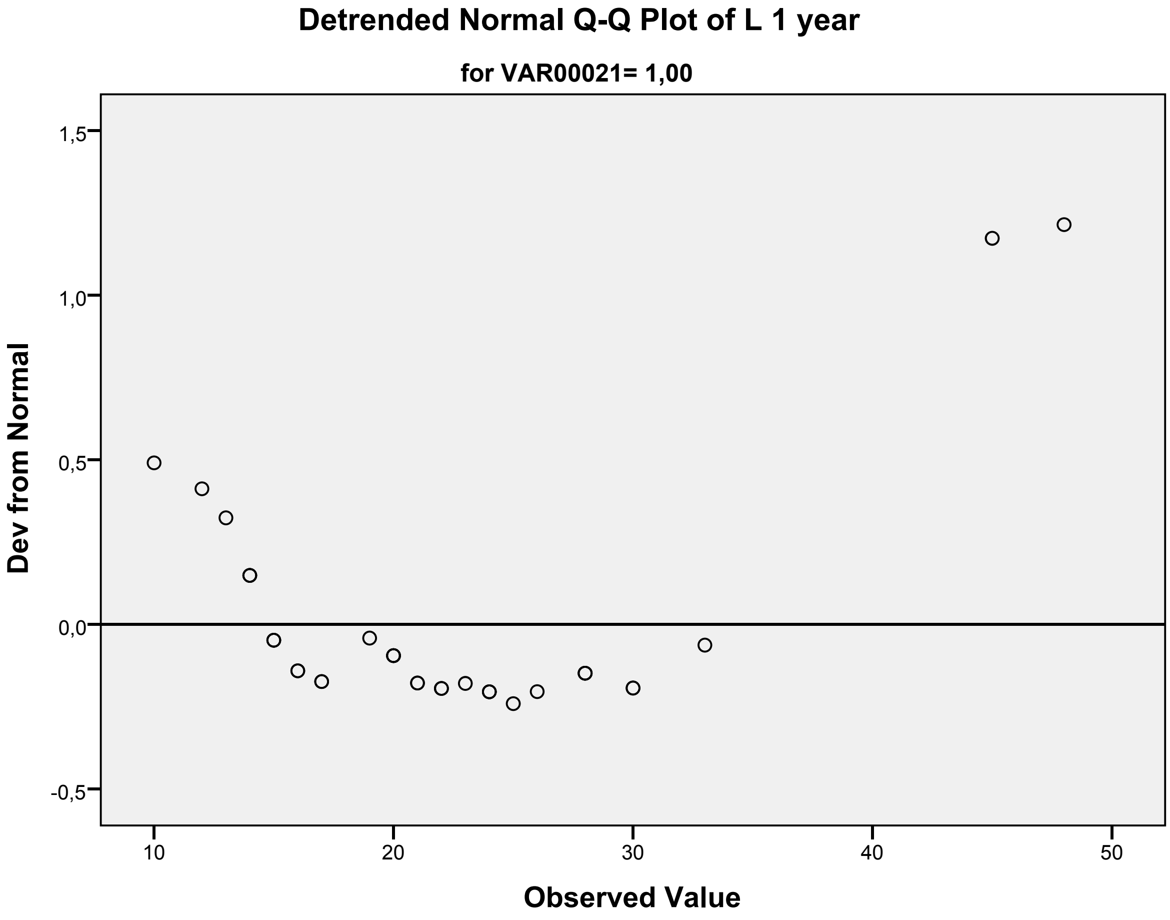
Detrended Normal Q-Q Plot of L 1 year
for VAR00021= 1,00
Dev from Normal
1,5
1,0
0,5
0,0
-0,5
10
20
30
40
50
Observed Value

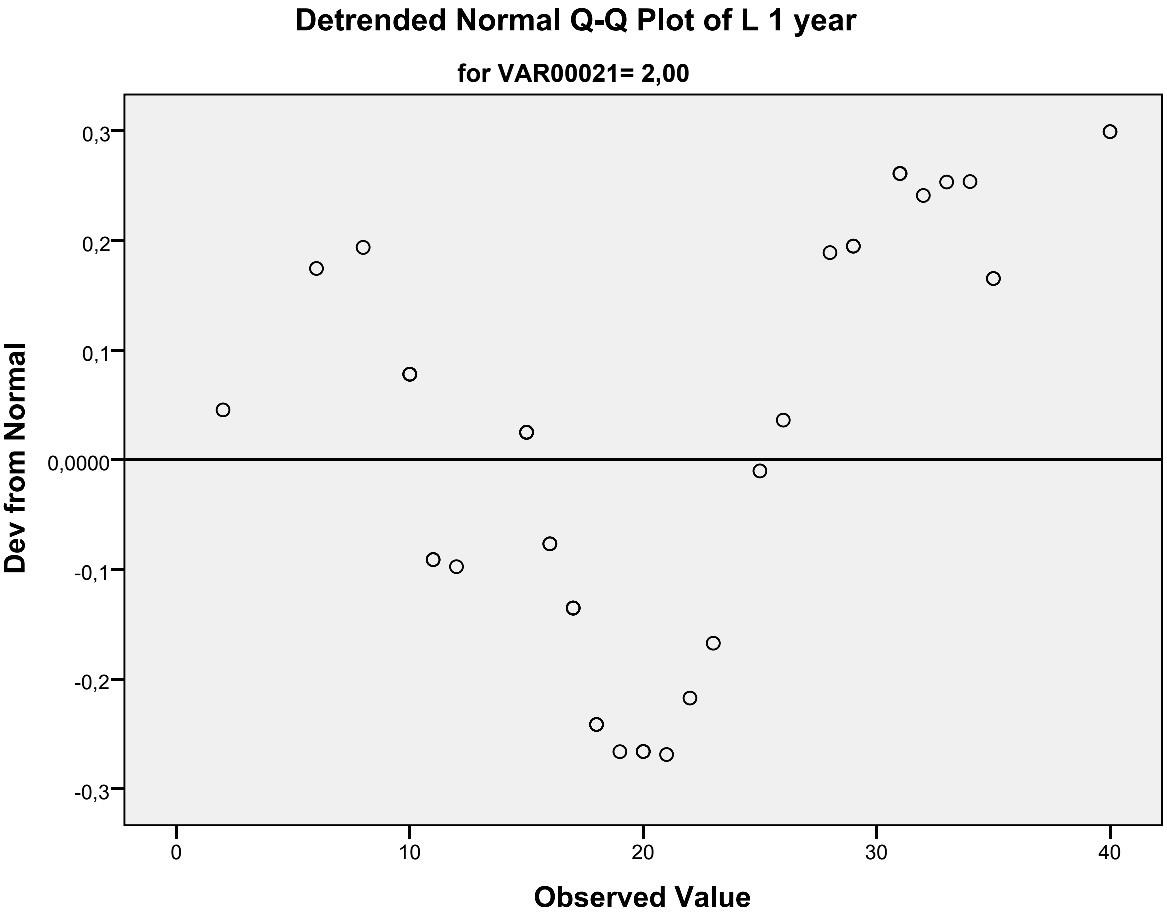
Detrended Normal Q-Q Plot of L 1 year
for VAR00021= 2,00
Dev from Normal
0,3
0,2
0,1
0,0000
-0,1
-0,2
-0,3
0
10
20
30
40
Observed Value

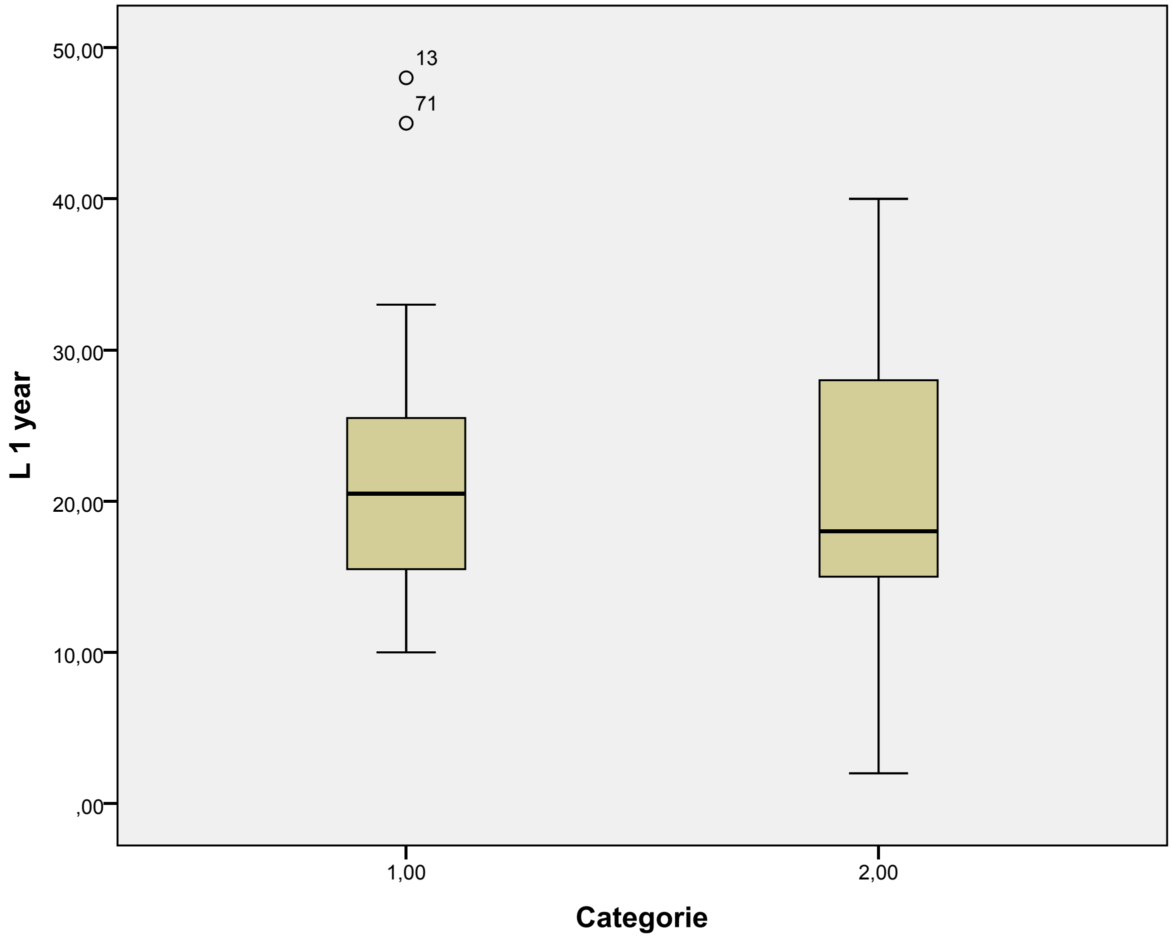
50,00
40,00
30,00
20,00
10,00
,00
13
71
L 1 year
1,00
2,00
Categorie
