# Supplementary material for: The new Lyon ARTbrace versus the historical Lyon brace: a prospective case series of 148 consecutive scoliosis with short time results after 1 year compared with a historical retrospective case series of 100 consecutive scoliosis; SOSORT award 2015 winner
Source: Scoliosis. 2015 Aug 19;10:26. doi: 10.1186/s13013-015-0047-6 (PMC4545553; doi:10.1186/s13013-015-0047-6)
Supplement: Additional file 3: — SPSS v 20: t-test comparing means of old Lyon and new Lyon ARTbrace. (PDF 398 kb) [file 13013_2015_47_MOESM3_ESM.pdf]

```
DESCRIPTIVES VARIABLES=VAR00006 VAR00007 VAR00010 VAR00011 VAR00012 VAR00013 VAR00014 VAR000
  /STATISTICS=MEAN STDDEV VARIANCE MIN MAX KURTOSIS.
```

Descriptives

Notes

Output Created		12-JUL-2015 11:23:27
Comments		
Input	Data	D:\SOSORT\2015 Katowice\SOSORT AWARD\6 months clinical.sav
	Active Dataset	Ensemble_de_données0
	Filter	<none>
	Weight	<none>
	Split File	<none>
	N of Rows in Working Data File	242
Missing Value Handling	Definition of Missing	User defined missing values are treated as missing.
	Cases Used	All non-missing data are used.
Syntax		DESCRIPTIVES VARIABLES=VAR00006 VAR00007 VAR00010 VAR00011 VAR00012 VAR00013 VAR00014 VAR00015 /STATISTICS=MEAN STDDEV VARIANCE MIN MAX KURTOSIS.
Resources	Processor Time	00:00:00,00
	Elapsed Time	00:00:00,01

```
[Ensemble_de_données0] D:\SOSORT\2015 Katowice\SOSORT AWARD\6 months clin
ical.sav
```

Descriptive Statistics

	N	Minimum	Maximum	Mean	Std. Deviation	Variance
	Statistic	Statistic	Statistic	Statistic	Statistic	Statistic
TrhT0	178	5	50	23,49	9,060	82,082
TbuT0	178	1	25	10,10	4,018	16,143
LrhT0	156	2	35	16,95	7,622	58,088
LbuT0	156	0	21	7,50	3,539	12,523
TrhT2	178	0	40	13,08	8,081	65,309
TbuT2	178	0	25	6,35	3,887	15,111
LrhT2	156	0	35	6,21	5,568	31,003
LbuT2	156	0	15	2,86	2,937	8,625
Valid N (listwise)	94					

Descriptive Statistics

	Kurtosis	
	Statistic	Std. Error
TrhT0	,006	,362
TbuT0	,752	,362
LrhT0	-,526	,386
LbuT0	,579	,386
TrhT2	1,008	,362
TbuT2	2,409	,362
LrhT2	4,896	,386
LbuT2	2,066	,386
Valid N (listwise)		

```
T-TEST GROUPS=VAR00002(1 2)
  /MISSING=ANALYSIS
  /VARIABLES=VAR00006 VAR00007 VAR00010 VAR00011 VAR00012 VAR00013 VAR00014 VAR00015
  /CRITERIA=CI(.95).
```

T-Test

Notes

Output Created		12-JUL-2015 11:24:18
Comments		
Input	Data	D:\SOSORT\2015 Katowice\SOSORT AWARD\6 months clinical.sav
	Active Dataset	Ensemble_de_données0
	Filter	<none>
	Weight	<none>
	Split File	<none>
	N of Rows in Working Data File	242
Missing Value Handling	Definition of Missing	User defined missing values are treated as missing.
	Cases Used	Statistics for each analysis are based on the cases with no missing or out-of-range data for any variable in the analysis.
Syntax		T-TEST GROUPS=VAR00002(1 2) /MISSING=ANALYSIS /VARIABLES=VAR00006 VAR00007 VAR00010 VAR00011 VAR00012 VAR00013 VAR00014 VAR00015 /CRITERIA=CI(.95).
Resources	Processor Time	00:00:00,02
	Elapsed Time	00:00:00,01

[Ensemble_de_données0] D:\SOSORT\2015 Katowice\SOSORT AWARD\6 months clinical.sav

Group Statistics

	Group	N	Mean	Std. Deviation	Std. Error Mean
TrhT0	1	77	23,56	8,608	,981
	2	101	23,44	9,432	,939
TbuT0	1	77	10,55	3,858	,440
	2	101	9,75	4,122	,410
LrhT0	1	51	16,41	7,365	1,031
	2	105	17,21	7,764	,758
LbuT0	1	51	7,47	3,378	,473
	2	105	7,51	3,630	,354
TrhT2	1	77	16,70	8,464	,965
	2	101	10,33	6,588	,656
TbuT2	1	77	7,95	4,049	,461
	2	101	5,14	3,293	,328
LrhT2	1	51	9,41	6,258	,876
	2	105	4,65	4,459	,435
LbuT2	1	51	4,51	3,215	,450
	2	105	2,06	2,429	,237

Independent Samples Test

		Levene's Test for Equality of Variances		t-test for Equality of Means	
		F	Sig.	t	df
TrhT0	Equal variances assumed	1,025	,313	,089	176
	Equal variances not assumed			,090	170,345
TbuT0	Equal variances assumed	,338	,562	1,307	176
	Equal variances not assumed			1,319	168,722
LrhT0	Equal variances assumed	,848	,358	-,612	154
	Equal variances not assumed			-,623	103,979
LbuT0	Equal variances assumed	,223	,637	-,072	154
	Equal variances not assumed			-,074	105,795
TrhT2	Equal variances assumed	3,154	,077	5,651	176
	Equal variances not assumed			5,466	139,764

Independent Samples Test

		t-test for Equality of Means			
		Sig. (2-tailed)	Mean Difference	Std. Error Difference	95% Confidence ... Lower
TrhT0	Equal variances assumed	,929	,123	1,375	-2,590
	Equal variances not assumed	,928	,123	1,358	-2,557
TbuT0	Equal variances assumed	,193	,793	,607	-,404
	Equal variances not assumed	,189	,793	,601	-,394
LrhT0	Equal variances assumed	,541	-,798	1,303	-3,373
	Equal variances not assumed	,534	-,798	1,280	-3,336
LbuT0	Equal variances assumed	,943	-,044	,606	-1,241
	Equal variances not assumed	,941	-,044	,591	-1,215
TrhT2	Equal variances assumed	,000	6,375	1,128	4,148
	Equal variances not assumed	,000	6,375	1,166	4,069

Independent Samples Test

		t-test for Equality of ...
		95% Confidence ...
		Upper
TrhT0	Equal variances assumed	2,835
	Equal variances not assumed	2,803
TbuT0	Equal variances assumed	1,990
	Equal variances not assumed	1,980
LrhT0	Equal variances assumed	1,777
	Equal variances not assumed	1,740
LbuT0	Equal variances assumed	1,153
	Equal variances not assumed	1,128
TrhT2	Equal variances assumed	8,601
	Equal variances not assumed	8,680

Independent Samples Test

		Levene's Test for Equality of Variances		t-test for Equality of Means	
		F	Sig.	t	df
TbuT2	Equal variances assumed	,573	,450	5,104	176
	Equal variances not assumed			4,965	144,114
LrhT2	Equal variances assumed	4,211	,042	5,459	154
	Equal variances not assumed			4,869	75,492
LbuT2	Equal variances assumed	6,595	,011	5,304	154
	Equal variances not assumed			4,821	78,668

Independent Samples Test

		t-test for Equality of Means			
					95% Confidence ...
		Sig. (2-tailed)	Mean Difference	Std. Error Difference	Lower
TbuT2	Equal variances assumed	,000	2,809	,550	1,723
	Equal variances not assumed	,000	2,809	,566	1,691
LrhT2	Equal variances assumed	,000	4,764	,873	3,040
	Equal variances not assumed	,000	4,764	,978	2,815
LbuT2	Equal variances assumed	,000	2,453	,462	1,539
	Equal variances not assumed	,000	2,453	,509	1,440

Independent Samples Test

		t-test for Equality of ...
		95% Confidence ...
		Upper
TbuT2	Equal variances assumed	3,896
	Equal variances not assumed	3,928
LrhT2	Equal variances assumed	6,488
	Equal variances not assumed	6,713
LbuT2	Equal variances assumed	3,366
	Equal variances not assumed	3,465

```
T-TEST GROUPS=VAR00021(1 2)
  /MISSING=ANALYSIS
  /VARIABLES=VAR00005 VAR00008 VAR00009 VAR00010 VAR00013 VAR00014 VAR0001
7 VAR00018
  /CRITERIA=CI(.95).
```

T-Test

[Ensemble_de_données1] D:\Bibliothèques\Documents\ARTbrace matched pair control.sav

Group Statistics

	Categorie	N	Mean	Std. Deviation	Std. Error Mean
T init	1,00	76	31,1447	9,62179	1,10369
	2,00	108	30,0370	8,30148	,79881
L init	1,00	59	26,6949	7,72201	1,00532
	2,00	87	27,8276	7,50536	,80466
T in-brace	1,00	76	16,9605	9,35442	1,07303
	2,00	108	11,2593	8,65796	,83311
Lin-brace	1,00	59	12,3220	7,93604	1,03318
	2,00	87	6,6437	8,74972	,93807
T 6 months	1,00	76	23,9605	10,95012	1,25606
	2,00	108	20,2500	10,77651	1,03697
L 6 months	1,00	59	18,8136	9,38722	1,22211
	2,00	87	15,5057	9,07575	,97302
T 1 year	1,00	76	26,9474	11,89386	1,36432
	2,00	108	21,4722	11,05505	1,06377
L 1 year	1,00	59	20,4068	10,01915	1,30438
	2,00	87	16,4023	9,37644	1,00526

Independent Samples Test

		Levene's Test for Equality of Variances		t-test for Equality of Means	
		F	Sig.	t	df
T init	Equal variances assumed	1,416	,236	,834	182
	Equal variances not assumed			,813	146,061
L init	Equal variances assumed	,049	,826	-,884	144
	Equal variances not assumed			-,880	122,274
T in-brace	Equal variances assumed	,039	,843	4,254	182
	Equal variances not assumed			4,197	153,563
Lin-brace	Equal variances assumed	,139	,710	3,993	144
	Equal variances not assumed			4,069	132,372
T 6 months	Equal variances assumed	,034	,854	2,284	182
	Equal variances not assumed			2,278	159,983
L 6 months	Equal variances assumed	,019	,891	2,131	144
	Equal variances not assumed			2,117	121,824
T 1 year	Equal variances assumed	,110	,741	3,205	182
	Equal variances not assumed			3,165	154,013
L 1 year	Equal variances assumed	,000	,986	2,463	144
	Equal variances not assumed			2,432	119,038

Independent Samples Test

		t-test for Equality of Means		
		Sig. (2-tailed)	Mean Difference	Std. Error Difference
T init	Equal variances assumed	,405	1,10770	1,32796
	Equal variances not assumed	,418	1,10770	1,36244
L init	Equal variances assumed	,378	-1,13267	1,28063
	Equal variances not assumed	,381	-1,13267	1,28769
T in-brace	Equal variances assumed	,000	5,70127	1,34025
	Equal variances not assumed	,000	5,70127	1,35848
Lin-brace	Equal variances assumed	,000	5,67836	1,42198
	Equal variances not assumed	,000	5,67836	1,39551
T 6 months	Equal variances assumed	,023	3,71053	1,62426
	Equal variances not assumed	,024	3,71053	1,62880
L 6 months	Equal variances assumed	,035	3,30781	1,55201
	Equal variances not assumed	,036	3,30781	1,56215
T 1 year	Equal variances assumed	,002	5,47515	1,70807
	Equal variances not assumed	,002	5,47515	1,73002
L 1 year	Equal variances assumed	,015	4,00448	1,62588
	Equal variances not assumed	,017	4,00448	1,64680

Independent Samples Test

		t-test for Equality of Means	
		95% Confidence Interval of the Difference	
		Lower	Upper
T init	Equal variances assumed	-1,51247	3,72787
	Equal variances not assumed	-1,58494	3,80034
L init	Equal variances assumed	-3,66394	1,39860
	Equal variances not assumed	-3,68172	1,41638
T in-brace	Equal variances assumed	3,05683	8,34570
	Equal variances not assumed	3,01755	8,38498
Lin-brace	Equal variances assumed	2,86771	8,48900
	Equal variances not assumed	2,91798	8,43874
T 6 months	Equal variances assumed	,50573	6,91533
	Equal variances not assumed	,49379	6,92726
L 6 months	Equal variances assumed	,24014	6,37548
	Equal variances not assumed	,21533	6,40030
T 1 year	Equal variances assumed	2,10497	8,84532
	Equal variances not assumed	2,05751	8,89278
L 1 year	Equal variances assumed	,79080	7,21816
	Equal variances not assumed	,74366	7,26531
